# Adapting and Evaluating an AI-Based Chatbot Through Patient and Stakeholder Engagement to Provide Information for Different Health Conditions: Master Protocol for an Adaptive Platform Trial (the MARVIN Chatbots Study)

**DOI:** 10.2196/54668

**Published:** 2024-02-13

**Authors:** Yuanchao Ma, Sofiane Achiche, Marie-Pascale Pomey, Jesseca Paquette, Nesrine Adjtoutah, Serge Vicente, Kim Engler, MARVIN chatbots Patient Expert Committee, Moustafa Laymouna, David Lessard, Benoît Lemire, Jamil Asselah, Rachel Therrien, Esli Osmanlliu, Ma'n H Zawati, Yann Joly, Bertrand Lebouché

**Affiliations:** 1 Department of Biomedical Engineering Polytechnique Montréal Montreal, QC Canada; 2 Centre for Outcomes Research & Evaluation Research Institute of the McGill University Health Centre Montreal, QC Canada; 3 Infectious Diseases and Immunity in Global Health Program Research Institute of McGill University Health Centre Montreal, QC Canada; 4 Chronic Viral Illness Service Division of Infectious Disease, Department of Medicine McGill University Health Centre Montreal, QC Canada; 5 Research Centre of the University of Montreal Hospital Centre Montreal, QC Canada; 6 Department of Health Policy, Management and Evaluation School of Public Health University of Montreal Montreal, QC Canada; 7 Centre of Excellence on Partnership with Patients and the Public Montreal, QC Canada; 8 Department of Family Medicine Faculty of Medicine and Health Sciences McGill University Montreal, QC Canada; 9 Department of Mathematics and Statistics University of Montreal Montreal, QC Canada; 10 See Acknowledgements; 11 Department of Medicine Division of Medical Oncology McGill University Health Centre Montreal, QC Canada; 12 Department of Pediatrics Montreal Children's Hospital McGill University Health Centre Montreal, QC Canada; 13 Centre of Genomics and Policy McGill University Montreal, QC Canada

**Keywords:** chatbot, master protocol, adaptive platform trial design, implementation science, telehealth, digital health, Canada, artificial intelligence, conversational agent, self-management, research ethics, patient and stakeholder engagement, co-construction, mobile phone

## Abstract

**Background:**

Artificial intelligence (AI)–based chatbots could help address some of the challenges patients face in acquiring information essential to their self-health management, including unreliable sources and overburdened health care professionals. Research to ensure the proper design, implementation, and uptake of chatbots is imperative. Inclusive digital health research and responsible AI integration into health care require active and sustained patient and stakeholder engagement, yet corresponding activities and guidance are limited for this purpose.

**Objective:**

In response, this manuscript presents a master protocol for the development, testing, and implementation of a chatbot family in partnership with stakeholders. This protocol aims to help efficiently translate an initial chatbot intervention (MARVIN) to multiple health domains and populations.

**Methods:**

The MARVIN chatbots study has an adaptive platform trial design consisting of multiple parallel individual chatbot substudies with four common objectives: (1) co-construct a tailored AI chatbot for a specific health care setting, (2) assess its usability with a small sample of participants, (3) measure implementation outcomes (usability, acceptability, appropriateness, adoption, and fidelity) within a large sample, and (4) evaluate the impact of patient and stakeholder partnerships on chatbot development. For objective 1, a needs assessment will be conducted within the setting, involving four 2-hour focus groups with 5 participants each. Then, a co-construction design committee will be formed with patient partners, health care professionals, and researchers who will participate in 6 workshops for chatbot development, testing, and improvement. For objective 2, a total of 30 participants will interact with the prototype for 3 weeks and assess its usability through a survey and 3 focus groups. Positive usability outcomes will lead to the initiation of objective 3, whereby the public will be able to access the chatbot for a 12-month real-world implementation study using web-based questionnaires to measure usability, acceptability, and appropriateness for 150 participants and meta-use data to inform adoption and fidelity. After each objective, for objective 4, focus groups will be conducted with the design committee to better understand their perspectives on the engagement process.

**Results:**

From July 2022 to October 2023, this master protocol led to four substudies conducted at the McGill University Health Centre or the Centre hospitalier de l’Université de Montréal (both in Montreal, Quebec, Canada): (1) MARVIN for HIV (large-scale implementation expected in mid-2024), (2) MARVIN-Pharma for community pharmacists providing HIV care (usability study planned for mid-2024), (3) MARVINA for breast cancer, and (4) MARVIN-CHAMP for pediatric infectious conditions (both in preparation, with development to begin in early 2024).

**Conclusions:**

This master protocol offers an approach to chatbot development in partnership with patients and health care professionals that includes a comprehensive assessment of implementation outcomes. It also contributes to best practice recommendations for patient and stakeholder engagement in digital health research.

**Trial Registration:**

ClinicalTrials.gov NCT05789901; https://classic.clinicaltrials.gov/ct2/show/NCT05789901

**International Registered Report Identifier (IRRID):**

PRR1-10.2196/54668

## Introduction

### Background

Self-management is key to patient health, and interventions to promote it are being increasingly implemented in the delivery of health care. Effective self-management involves multiple aspects, including problem-solving, decision-making, resource use, patient–health care professional partnership building, and taking action [1,2]. To improve self-management, patients often seek guidance from health care professionals, staff and volunteers from community organizations or peers, and a variety of internet resources [3,4]. However, the availability of these actors and resources can be inconsistent. Moreover, information encountered on the internet varies in quality [5-7]. From reliable but complex scientific articles to opinion blogs or outdated web pages, these sources do not always provide the clearest, most accurate, or most reassuring guidance. The importance of self-management has been further highlighted by the COVID-19 pandemic, which introduced additional barriers to accessing regular follow-up [8] and exposed individuals worldwide to an infodemic [9,10].

In addition to these challenges, frontline professionals are often confronted with complex questions from patients regarding treatment instructions, comorbidity management, side effects, and drug interactions. However, in the context of swiftly evolving knowledge and overwhelming workloads, health care professionals may not have sufficient expertise and time for certain questions [11,12]. Their responses may vary depending on their individual training, proficiency, and clinical experience. Rapidly obtaining accurate and clear health information and relaying it to patients can be a daunting challenge for professionals.

Safe and effective digital health interventions could help address the limitations of existing self-management or care support. A particularly promising avenue is the emergence of artificial intelligence (AI)–based chatbots—software applications that interact with users through simulated human text or voice conversations via smartphones or computers. Often harnessing AI to enable natural language interpretation and assist in decision-making, such tools possess the potential to revolutionize patient self-management and support [13]. They can be applied to diverse platforms to foster mutually beneficial outcomes for health systems and patients, including less time spent in hospitals, outpatient efficiency, and personalized treatment [9].

Successful implementation of an intervention (ie, positive implementation outcomes) is necessary to achieve desired changes in clinical or service outcomes [14]. Multiple studies have investigated the implementation of health-oriented chatbots, including in the areas of mental health support [15-17], problematic substance use treatment [18], cirrhosis patient education [19], and asthma self-management [20]. Nevertheless, such studies have typically focused on feasibility and usability in small-scale prototype implementations [21-23]. Digital health products are often dealt with through an “implement now, clinically validate later” ethos [24], as they encompass a large number of different technologies. Thus, there is no clear consensus on methods for assessing the clinical effectiveness of digital health interventions [24], and data on the impact of chatbot interventions on clinical outcomes are scarce [25]. Large user samples are necessary to gain more robust insights into chatbot implementation. In-depth studies including other valuable implementation (eg, fidelity, appropriateness, sustainability, and cost-effectiveness) and clinical (eg, safety, effectiveness, and efficacy) outcomes will also be critical for scaling up and long-term adoption of chatbot interventions.

Finally, very little engagement of stakeholders, including patients and health care professionals, has led to poor usability and low adoption of many digital health interventions [26]. In the context of chatbot development, a scoping review investigating patient engagement revealed limited involvement of patients and insufficient reporting of the relevant activities [27]. Among the 16 studies included, only 8 mentioned patient engagement, with just 3 offering adequate details on the methods and approach used. The authors also pointed out that future chatbot development would need to integrate multifaceted means of patient participation and document them thoroughly. Stakeholders should be engaged in defining research objectives and designing interventions tailored to their needs [28]. According to the patient-public partnership continuum proposed by the Montreal model [29], this inclusivity can be extended to “co-construction,” where patients and stakeholders are involved throughout the process. Meanwhile, to answer the many questions raised by the use of intelligent machines and ensure that AI develops in harmony with democracy, the responsible integration of AI necessitates a co-construction process [30]. Engaging end users in discussions about the challenges posed by AI and drawing on their lived experiences can reveal key aspects of digital health research that might otherwise be overlooked [31], thus better paving the way for success.

Since 2020, led by YM, SA, and B Lebouché, an innovative chatbot named *Minimal AntiRetroViral INterference* (now named *MARVIN*) has been in development for people with HIV. Through a co-design approach involving patients and stakeholders, MARVIN aims to facilitate antiretroviral therapy self-management. The authors’ team subsequently trialed the MARVIN chatbot among people with HIV and validated its usability and acceptability [32]. Given the initial success of MARVIN among people with HIV, we intend to build on this pilot study to increase MARVIN’s areas of specialization and continue to develop our algorithms to improve MARVIN’s intelligence, thereby expanding its reach and potential benefits to a broader audience.

### Aim and Objectives

Grounded in patient and stakeholder engagement strategies and implementation science, this protocol aims to describe the methods and tools necessary to efficiently develop the innovative MARVIN chatbot interventions across multiple health domains and populations and assess their implementation for robust and widespread use. The study’s primary objectives are to co-construct versions of MARVIN adapted for different health conditions and target populations (objective 1: development), assess their global usability (usability and acceptability) in a small participant sample context (objective 2: usability), and measure implementation outcomes (usability, acceptability, appropriateness, adoption, and fidelity) in the context of a large sample (objective 3: implementation) through a mixed methods approach in the respective setting of each chatbot. The secondary objective is to evaluate the impact of different stakeholder partnerships established for the aforementioned objectives on the development of the AI health care chatbots (objective 4: partnership evaluation).

## Methods

### Study Design

This multicenter study follows the CONSORT (Consolidated Standards of Reporting Trials) extension for pilot and feasibility trials [33], CONSORT-EHEALTH (Consolidated Standards of Reporting Trials of Electronic and Mobile Health Applications and Online Telehealth) [34], and CONSORT-AI (Consolidated Standards of Reporting Trials–Artificial Intelligence) [35] guidelines (Multimedia Appendices 1-3 [33]), as well as the Montréal Declaration for a Responsible Development of Artificial Intelligence [30] regarding the ethical conduct of research involving humans and AI.

The study is presented in the form of a master protocol, defined as a single overarching design developed to evaluate multiple hypotheses with the overall goal of increasing efficiency and establishing uniformity through the standardization of procedures in the development and evaluation of different interventions [36]. This master protocol will encompass multiple parallel individual chatbot projects sharing the MARVIN chatbot technology, subsequently referred to as “substudies.” The chatbot of each substudy is considered as a distinct intervention as it will integrate specific features or content tailored to the corresponding health care setting (health condition and target population). The chatbots will be implemented in these different populations without control groups.

To accommodate this, we used an adaptive platform trial design, which combines features of both basket trials (designed to test a single intervention in different populations) and platform trials (designed to test multiple interventions in the context of a single disease) [37]. As defined by the US Food and Drug Administration, an adaptive platform design is appropriate for our trial as it allows for flexibility in managing multiple interventions adapted to different populations while enabling the early removal of ineffective interventions and introduction of new interventions based on interim data [38].

**Figure 1:**
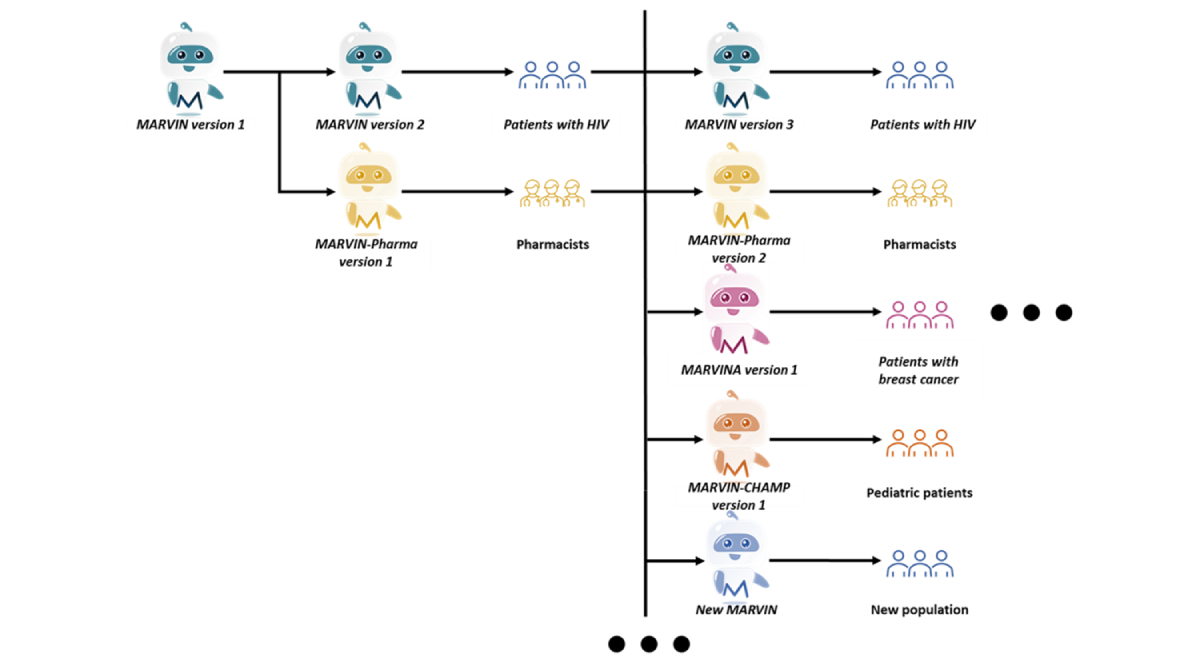
Adaptive platform trial design without control group.

**Figure 2:**
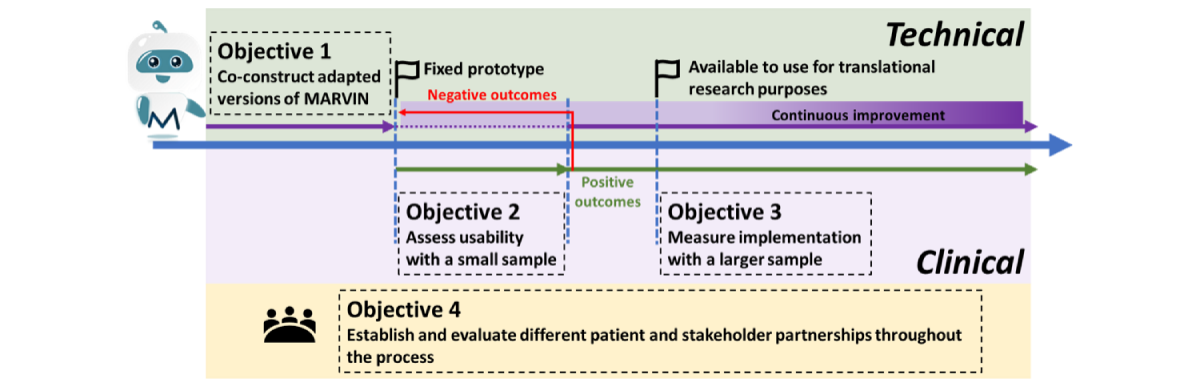
Study steps for each substudy.

As shown in the example in Figure 1, substudy arms targeting different conditions can be initiated at different time points. The substudies are independent of each other, and their processes are illustrated in Figure 2. Objectives 1 to 3 will be completed in a sequential manner, whereas objective 4 will be assessed throughout the process. A decision will be made on whether to continue with the same chatbot intervention version in objective 3 (implementation) based on the results of objective 2 (usability). In addition, there is no initial fixed duration or sample size for each substudy.

This master protocol outlines the common elements of the individual chatbot substudies in terms of objectives and processes. Concurrently, each substudy will have its own subprotocol describing more specific standardized operational structures; additional inclusion and exclusion criteria; recruitment and selection methods; and data collection, analysis, and management. All subprotocols will be managed as appendices to this master protocol and will be subject to further review by the research ethics board (REB) when they are ready. In addition, certain criteria of the master protocol may be redefined by the research team based on progress in each substudy and submitted as amendments to the REB for review for subsequent application to all substudies.

### Ethical Considerations

This study received approval from the McGill University Health Centre (MUHC) REB on August 9, 2023 (approval MP-37-2023-9333); the Centre hospitalier de l’Université de Montréal REB (approval MEO-37-2024-11732) on October 13, 2023; and the Polytechnique Montréal REB (approval CER-2324-29-D) on October 10, 2023. It is also registered in the ClinicalTrials.gov database (NCT05789901).

### Settings and Participants

This study will be conducted at the MUHC and the Centre hospitalier de l’Université de Montréal, both located in Montreal, Quebec, Canada. The study participants include patients and health care professionals, who will be the end users of the chatbots.

### Eligibility Criteria

The primary inclusion criteria for participants are as follows: (1) age of ≥18 years; (2) fluency in English or French; (3) ability to understand the requirements of study participation and provide oral and written informed consent before and during the implementation of the study; (4) access to a smartphone, tablet, or computer in a private environment; and (5) access to an internet connection or data plan on their device. Specific to objectives 2 (usability) and 3 (implementation), additional inclusion criteria are (6) acceptance of using or creating a personal Facebook (Meta Platforms) account, (7) acceptance of using a Facebook Messenger-based chatbot, and (8) acceptance of Facebook’s privacy and data security policies.

Participants may not take part if they (1) are affected by a cognitive deficit or medical instability that prevents them from participating in any aspect of the study and (2) self-report being insufficiently able to use the chatbot with the technical support provided. For objectives 2 (usability) and 3 (implementation), the patient partners involved in the co-construction design committee (defined in the following section) will not be able to participate in either phase.

### Recruitment and Sample Size Justification

Objectives 1, 2, and 3 will use convenience sampling to recruit participants through different channels, including clinics, patient foundations, community-based organizations, and professional associations [39]. For example, patient participants will be introduced to the study by their health care service provider (eg, physician, nurse, or social and community worker) during their visits. Informational materials such as study flyers and a video (Multimedia Appendix 4) showcasing the MARVIN chatbot will be disseminated via email, newsletters, or a website.

Participants will be required to consent before engaging in each objective. For objectives 1 (development) and 2 (usability), interested individuals can reach out to the study coordinator. Detailed study procedures, eligibility checks, and consent collection will be facilitated by the study coordinator for those expressing interest. Verbal consent may be adopted for participants who continue from objective 1 to objective 2. This will be obtained remotely through teleconference with an impartial witness to ensure compliance with all aspects of free and informed consent. Following the co-construction approach [30], a co-construction design committee consisting of researchers, health care professionals, and volunteer patient partners will be formed starting from objective 1 to carry out the subsequent development. The sample size for objective 1 is 20 participants to ensure saturation for the needs assessment focus groups [40], whereas 30 participants will be recruited to complete the corresponding usability test for objective 2. This sample size is common for pilot studies and satisfies the minimum size recommended in the literature [41,42].

For objective 3 (implementation), an optimized version of the chatbot will be accessible from the web around the clock, 7 days a week, for translational research. As currently the MARVIN chatbots will be only released in Canada, recruitment will be exclusive to the Canadian population, and participants will engage in an electronic consent process through the MARVIN chatbots. Before using the chatbot, individuals will be required to review and accept MARVIN’s privacy policy (Multimedia Appendix 5). Subsequently, they will be asked to review an electronic version of the information and consent form and then answer the following verification questions for eligibility criteria 1 to 3 via the chatbot: (1) *Are you at least 18 years old?* (2) *Are you comfortable using English or French while communicating with the chatbot?* (3) *Do you agree to participate in the study as described above?* The remaining criteria (4 to 8) will be met once users connect to the chatbot. Should participants agree to participate, their responses will be securely recorded in a separate encrypted database on the MARVIN cloud servers and synchronized to the electronic enrollment log. If users opt not to participate, no records will be kept. As a Facebook account will be a prerequisite for using the chatbot, no further measures will be taken to detect or prevent the possibility of multiple identities. The target sample size of 30 to 150 participants was obtained based on the usability, acceptability, and appropriateness outcomes, which are considered key outcomes for this objective. The analysis involves a 1-sided Student *t* test (1-tailed) evaluating whether the corresponding average attains a predetermined threshold. A power analysis for a 1-sample *t* test is then performed, with 80% power and a 5% significance level. Within the targeted sample size (30≤n≤150), small to moderate standardized effect sizes (0.2≤Cohen *d*≤0.5) are detectable in the total sample with the aforementioned statistical power.

Regarding objective 4 (partnership evaluation), upon completion of each objective, an email invitation will be sent to organize focus groups with stakeholders involved in the chatbot co-construction design committee.

The information and consent forms outline detailed study procedures, anticipated benefits, and potential risks. All template versions are available in Multimedia Appendix 6. Participants may withdraw from the study at any time after providing informed consent. This information will be recorded in the electronic enrollment log, and the related privacy management and protection measures will be detailed later. To protect the participants’ personal data and identity, no identifying information will appear in any manuscript or report from this study.

### Intervention: Status Quo of the MARVIN Chatbot

#### Overview

Running on Messenger 24/7 for free [43], MARVIN was created as a bilingual chatbot in both English and French trained to converse with people with HIV on the following self-management aspects: (1) guidance for antiretroviral therapy medication (with regard to time management, dosing, drug interactions, and medication reminders, among other things), (2) antiretroviral therapy management when traveling, and (3) common HIV-related knowledge (eg, symptoms, modes of transmission and prevention, and vaccination recommendations).

The team conducted the first pilot project (MUHC REB 2021-7191) in April 2021 with 28 people with HIV receiving treatment at the MUHC. The study results showed that MARVIN was tailored to patient requirements and was easy to use and approachable but that the chatbot’s comprehension had limits [32]. For example, if a patient asked a question that was outside the range of topics in the question bank or was worded differently, the chatbot did not always understand it. Nonetheless, considering the development phase, participants reported being satisfied with MARVIN and mentioned that they intended to use it. Thus, by talking to the on-call chatbot, people with HIV could obtain the information they needed for self-management.

#### AI Algorithms and Strategies

**Figure 3:**
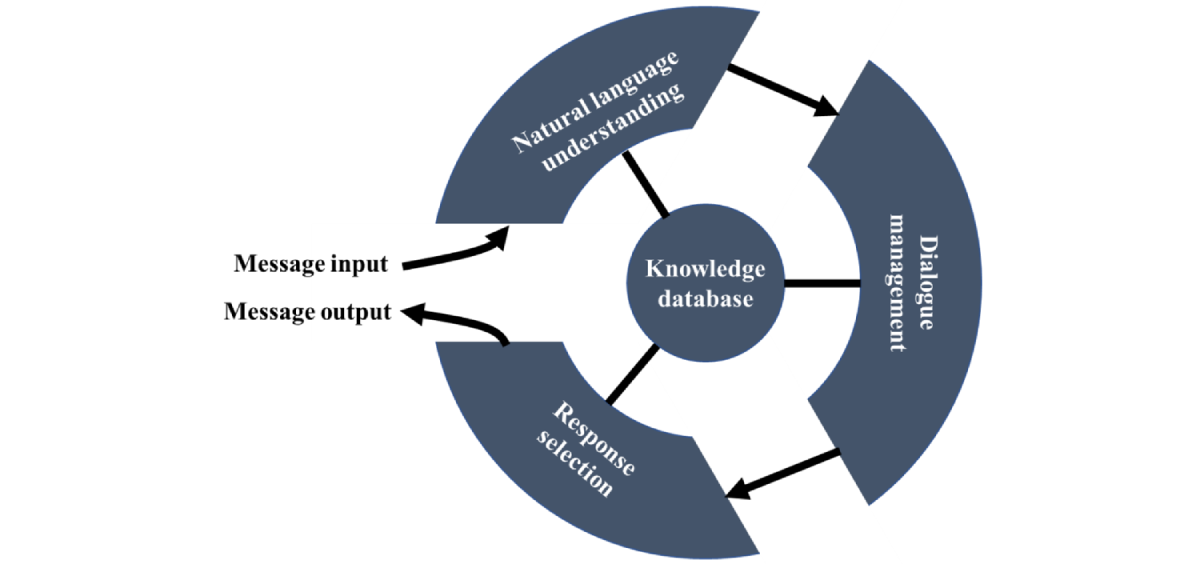
Operation process of MARVIN.

MARVIN is currently being developed using the Rasa platform (Rasa Technologies Inc), an open-source machine learning framework for automating text- and voice-based virtual assistants [44]. As shown in Figure 3, MARVIN’s architecture comprises 3 distinct modules: natural language understanding, dialogue management, and response selection. Together with a self-built knowledge database, these modules are used to process the message input and generate the message output.

**Figure 4:**
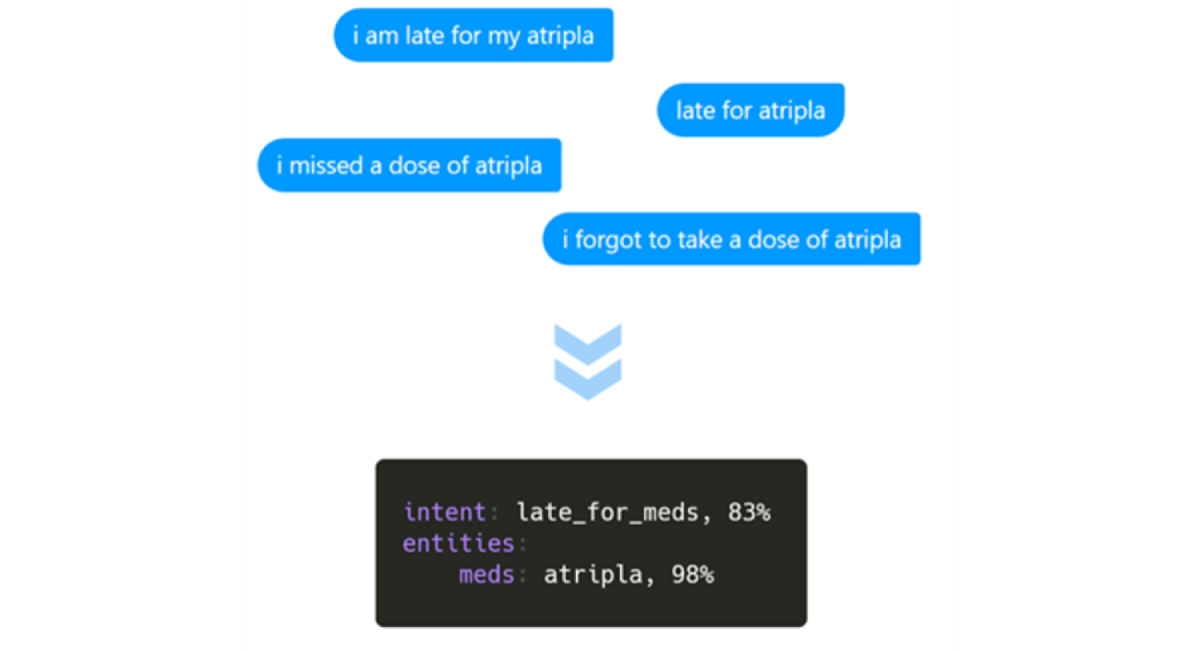
Example of text processing in MARVIN.

The acceptable input data include natural language in text form as well as some auxiliary expressions commonly used for chatting (eg, emojis such as the thumbs up, smiley face, and sad face). The initial natural language understanding module allows MARVIN to semantically process the input messages through different algorithms such as text preprocessing, entity extraction, and intent classification. The current training data set encompasses a corpus of >3000 questions covering >30 topics. Figure 4 shows an example of text processing. The entity extraction mechanism enables the chatbot to obtain structured information (eg, date, time, and medication name) from the input messages, whereas intent classification helps MARVIN discern the purpose of the information received.

**Figure 5:**
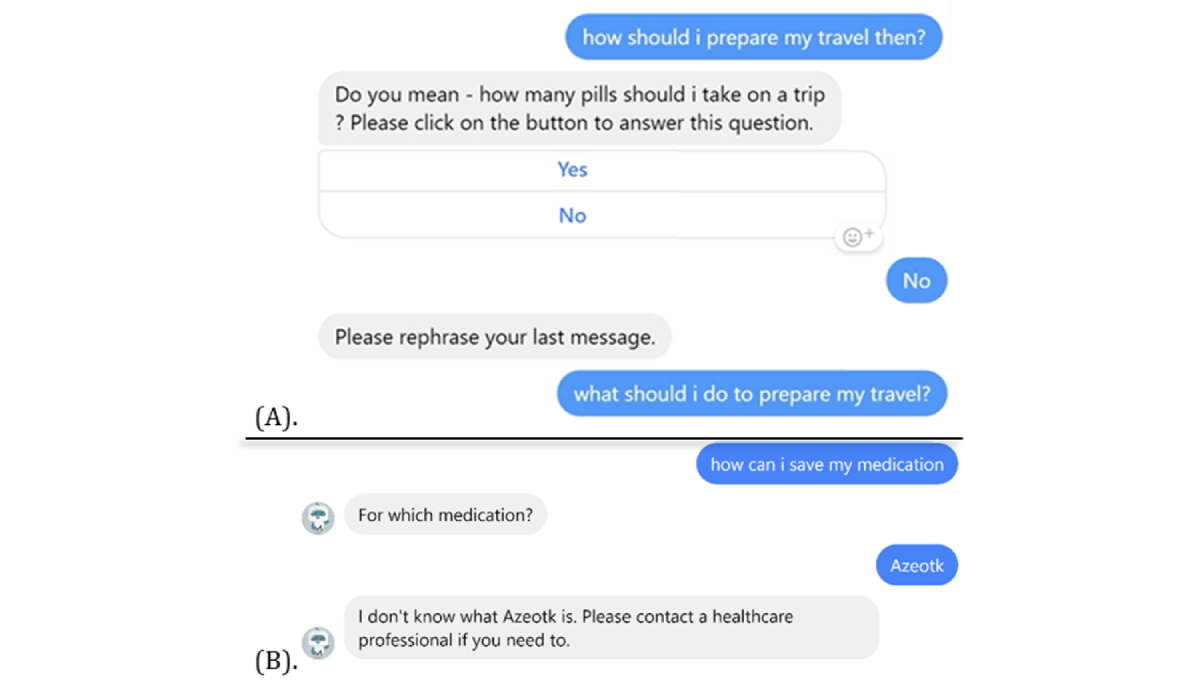
Examples of MARVIN handling bad inputs: (A) fallback policy; (B) redirection after 2 failed attempts.

In particular, MARVIN adopts the *FallbackClassifier* algorithm as a fail-safe measure for unprocessable input forms such as images, videos, and sounds. It is also activated when the confidence level of the anticipated intent falls below an established threshold. If the input remains incomprehensible after 2 attempts, the chatbot responds in a uniform manner: “I’m sorry, I can’t understand your question right now. Please contact a healthcare professional if you need to.” As illustrated in Figure 5, this approach reduces the probability of the chatbot taking incorrect actions when confronted with ambiguity.

The machine learning–driven conversation management determines MARVIN’s subsequent actions based on the input message and context in the conversation. A hybrid strategy of the *memoization policy* and *rule policy* was adopted [45]. The *memoization policy* remembers the decision trees from the training data and predicts the next action in conjunction with the derived intention: asking clarifying questions, providing tailored answers, and taking a fallback action. MARVIN can also remember and analyze a certain number of rounds of dialogue to support the prediction. Meanwhile, the *rule policy* handles pieces of conversation that should always follow the fixed behavior defined in the training data (eg, question: *What is ART?* answer: *It means AntiRetroviral Therapies*).

**Figure 6:**
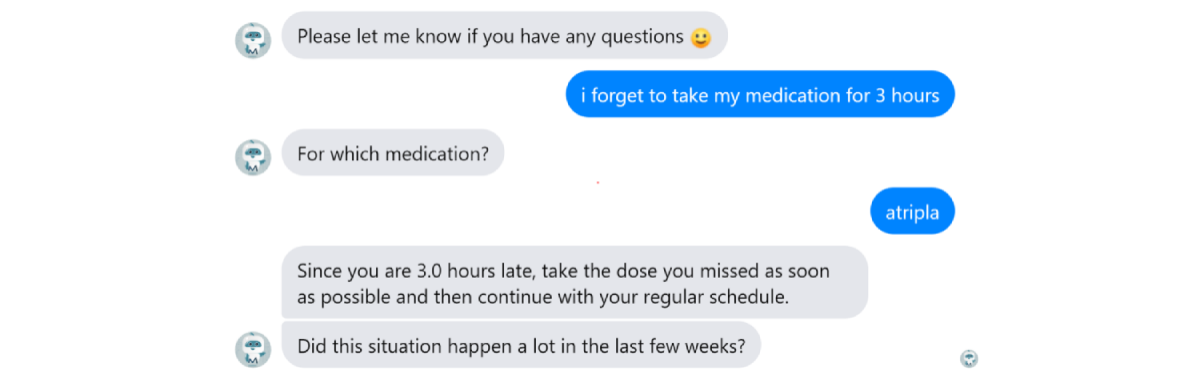
Conversation example with MARVIN.

The final response selection module enables MARVIN to select messages predefined by the health care team as output messages. All the data required for processing by the first 3 modules are stored in the knowledge database—after each decision is made in the processing module, MARVIN communicates with the database to compare, extract, or save the data. All data are selected, edited, and validated by the medical team and then processed and added to the database by the development team. Data types include the previously mentioned antiretroviral therapy management–related information, decision trees for different problem scenarios, and predefined answers. An example of response selection from predefined answers in MARVIN is shown in Figure 6.

#### Content Development and Improvement Process

To train MARVIN to understand different types of questions and provide relevant and accurate answers, we assembled a multidisciplinary team of 2 clinicians (ML and B Lebouché), 2 pharmacists (B Lemire and RT), 4 patients, 2 engineers (YM and SA), and multiple developers. Each group’s expertise played a crucial role in chatbot content development and continuous improvement: (1) health care professionals validated the medical accuracy of the answers and information given by the chatbot; (2) together with patient partners, they collaborated to identify common self-care challenges; (3) engineers then assisted in building decision models and preparing chatbot training data based on identified problems; and (4) finally, engineers and patients collaborated on testing and refining the chatbot based on feedback, completing the interdisciplinary cycle. This multidisciplinary team allowed us to ensure the accuracy and quality of the information provided as well as conduct holistic research and ongoing evaluation of the chatbot to promote its long-term usability.

#### The Interface

Messenger has 1.3 billion monthly active users worldwide exchanging 8 billion messages per day [46]. This indicates a robust familiarity with the Messenger interface among the population, simplifying their grasp of the platform’s specifications and interaction capabilities, ultimately favoring the uptake of the MARVIN chatbots. In addition, the versatility of Messenger, compatible across smartphones, tablets, and desktops, reduces the potential risk of participant exclusion because of hardware limitations. Nevertheless, the privacy concerns brought up with Facebook in the past may make people hesitant, which could be one of the potential barriers to the implementation of Messenger for health purposes. Indeed, other user interface options such as a stand-alone mobile app, a web page, or other third-party applications (eg, Telegram or Instagram) are under consideration. As the study proceeds and evolves, relevant changes will be implemented as necessary.

### Main Study Process

#### Overview

Multimedia Appendix 7 illustrates the entire study process.

#### Objective 1: Co-Construction of the MARVIN Chatbots

##### Overview

Objective 1 is intended to obtain a stabilized version adapted to the health care context for the subsequent objectives. Similar to the original MARVIN’s development, it will follow 3 steps: user needs assessment, knowledge database creation, and continuous improvement of the prototype.

The expected duration of participant involvement in objective 1 is 4 months.

##### Step 1: Needs Assessment

Four 2-hour focus groups will be organized with 5 participants each led by a trained interviewer. Participants will first be shown a demonstration of the current MARVIN describing the interface, dialogue process, and other instructions for use. Semistructured focus groups will then be conducted to identify use scenarios, topics related to integration, and user expectations and preferences for chatbots.

The co-construction design committee will then be formed and meet every 2 weeks to participate in design tasks. The team may also contact them via email with specific questions.

##### Step 2: Knowledge Database Creation

A knowledge database will be built based on the results of the needs assessment, which is the core of each new chatbot. This database will include a bank of different questions, plausible answers, and necessary conversation templates. Among the most important components of this knowledge database is a corpus of qualified and trusted answers. The challenge is to generate medically accurate information that is easy to understand and sufficiently colloquial while maintaining professionalism. These data will be collected from different sources and validated by the co-construction design committee for adoption to ensure that the chatbot can respond with appropriate output [29,30]. A total of three 2-hour co-construction workshops will be conducted.

##### Step 3: Testing, Validation, and Continuous Improvement

The team of engineers will work closely with the co-construction design committee through development workshops to test the prototype’s performance, especially its quality and safety. Participants will be invited to converse with the test prototype and complete an assessment. Such an approach will facilitate the continuous improvement of the prototype and the addition of new features (eg, user interface, questioning methods, and confidentiality measures) as necessary. Thus, each chatbot will evolve in real time during this step. A total of three 2-hour development workshops will be conducted.

It is important to note that, given the nature of software development engineering, steps 2 and 3 will be a continuous cyclic process. The research team will need to continually update the MARVIN chatbots based on feedback from the co-construction design committee on new requirements, improvement ideas, and technology updates.

##### Quantitative Data Collection and Analysis

During each development workshop, co-construction design committee members will perform a quick descriptive assessment of the tested prototype using the adapted Mobile App Rating Scale for health-related apps [47] (Multimedia Appendix 8).

The scale has 28 items (range 1-5) in 6 sections covering subjects including engagement, functionality, esthetics, information, subjective quality, and health-related quality. Item scores will be averaged to obtain a score for each section and an overall score. It has shown an excellent internal consistency (Cronbach α=.938) and interrater reliability (2-way mixed intraclass correlation coefficient=0.920, 95% CI 0.797-0.987) for the independent overall score ratings of 37 different digital health tools [47]. On the basis of developer recommendations, we will consider a successful prototype for testing to have an average score of at least 4 for the sections on information and health-related quality, as well as an overall average score of 4, as they are identified as key indicators of prototype safety.

##### Qualitative Data Collection and Analysis

Throughout the study, participants will have the option to take part in either English or French focus groups and workshops. All activities will be moderated by an experienced researcher with an assistant, digitally audio recorded, and manually transcribed verbatim for analysis while removing any nominal information provided to protect the participants’ identity. NVivo R1 (QSR International) will be used for qualitative data management.

Participants will also receive transcripts of the sessions in which they participated to ensure the trustworthiness of these data. Therefore, they will have the opportunity to challenge information that is perceived as incorrect. This will also allow them to verify that no information that could potentially identify them was inadvertently retained [48].

A focus group guide will be developed for each substudy considering its specific setting. Focus groups will be analyzed using an inductive thematic analysis approach to gather user recommendations (ie, topics to be addressed, expected conversational style, and desired features). Qualitative workshop data will contribute to thematic analysis in pursuit of objective 4.

#### Objective 2: Usability Assessment

##### Overview

Although a usability study among people with HIV has already been conducted [32], usability will be evaluated for other individual chatbot substudies following the same methodology. During this stage, no updates will be made to the chatbots unless (1) the chatbots are not available because of force majeure (eg, host server failure or algorithm dependency update), in which case the team will implement updates to ensure the proper conduct of the study; (2) the results indicate suboptimal usability, in which case we will update the version and repeat the usability study; or (3) new medical information emerges that could benefit participants or prevent harm. Any such event will be documented in detail.

The expected duration of participation for objective 2 is 1 month. At enrollment, 30 participants will be required to complete an initial sociodemographic questionnaire. They will be given a training session and a user guide with instructions on how to access MARVIN via Messenger and the topics of questions they can ask MARVIN. Participants will be enrolled for a 3-week period of interacting with MARVIN by having at least 20 conversations, the topics of which will be specified in each subprotocol. Quantitative data will be collected using a usability survey once their 20 conversations are recorded.

Participants will be free to complete the tasks at any time during this 3-week period. If the chatbot does not receive a message from them within a week of their most recent conversation, it will proactively send a reminder. There will be no active third-party human involvement in the entire testing process between the chatbot and participant user except in the case of user-initiated requests (eg, to solve unexpected bugs).

In week 4, a total of 3 focus groups with 5 randomly selected participants each will be conducted to explore MARVIN’s usability in greater depth and in the participants’ own words. In total, 3 focus groups can capture at least 80% of the themes, which is sufficient saturation for a usability study [49].

##### Quantitative Data Collection and Analysis

The initial questionnaire for objective 2 will gather fundamental sociodemographic information (eg, year of birth, preferred language, gender, and ethnic group identity) and digital technology use (Multimedia Appendix 8).

Descriptive statistics will be used to depict the sociodemographic characteristics and digital technology use of the participants. Continuous variables will be reported using measures such as minimum, maximum, mean, and SD. In the case of ordinal and nominal qualitative variables, we will report both counts and proportions.

Global usability will be collected using 2 validated scales: the *shorter version of the Usability Metric for User Experience* (*UMUX-Lite*) [50] and the *Acceptability E-scale* (*AES*) [51] (Multimedia Appendix 8).

Usability is defined in part as “the extent to which a product can be used to be effective, efficient, and provide users’ satisfaction within its defined goal” [52]. The UMUX-Lite is a 2-item questionnaire answered on a 7-point Likert scale that is deemed appropriate for use in the evaluation of health technology [53]. The items ask whether the chatbot meets user needs and about perceived ease of use.

Acceptability is related to how agreeable, palatable, or satisfactory an intervention is perceived to be by stakeholders and is also considered part of global usability [14]. The AES contains 6 items rated on different 5-point Likert scales. It is a validated measure of the acceptability and usability of computer-based interventions for health care populations. Items evaluate, for example, how easy and enjoyable the innovation is to use, how helpful it is, and whether the amount of time to engage with it is acceptable.

As continuous outcomes, both usability and acceptability will be summarized using the minimum, maximum, mean, and SD. The sample mean of each global usability outcome will be compared with its recommended usability thresholds—68/100 for the UMUX-Lite score and 24/30 for the AES score—using a Student *t* test. It will test the null hypothesis that the average UMUX-Lite score is ≤68 and that the average AES score is ≤24. A significance level of 5% will be adopted.

Four central subconstructs of the Technology Acceptance Model (TAM) framework will also be assessed as secondary end points via validated instruments to complement the data: (1) perceived ease of use, (2) perceived usefulness, (3) attitude toward use, and (4) behavioral intention to use the chatbots.

Perceived ease of use will be measured using the Single Ease Question [54], which is answered on a 7-point Likert scale.

Drawing on instruments by Chau and Hu [55] and Davis [56], perceived usefulness will be measured on a 7-point Likert scale with 4 items slightly adapted for relevance to chatbots. The final score will be the average of these items.

Attitude toward using chatbots will be measured using the net promoter score (NPS) [57], which is used as a measure of user satisfaction. A single question will be asked on an 11-point Likert scale. To calculate the NPS, 3 groups are created: promoters (score of 9-10), passives (score of 7-8), and detractors (score of 0-6). Subtracting the percentage of detractors from that of promoters provides the NPS (range −100 to 100). Positive scores, and especially those of >50%, are judged positively.

Finally, behavioral intention to use the chatbots will be assessed using a validated 2-item questionnaire [58] rated on a 7-point Likert scale averaged to produce a final score.

Predicted positive associations between subconstructs within the TAM framework will be evaluated using simple linear regressions considering the slope coefficient. Their significance will be tested using a Student *t* test.

All statistical analyses will be conducted using the R software (R Foundation for Statistical Computing) [59].

##### Qualitative Data Collection and Analysis

Participants’ experiences with MARVIN will be explored through a semistructured focus group on the main constructs of the TAM framework (ie, usefulness, ease of use, attitude, and behavioral intention to use), the analysis of which will help further explain the quantitative analysis. A focus group interview guide can be found in Multimedia Appendix 8.

A composite coding matrix based on the TAM and the nonadoption, abandonment, scale-up, spread, and sustainability (NASSS) framework will be favored for deductive content analysis [60]. The NASSS framework [61] was developed to support the implementation and scale-up of technological innovations in health care. It includes seven relevant domains: (1) the illness or condition, (2) the technology, (3) the value proposition, (4) the adopter system, (5) the organization, (6) the wider context, and (7) embedding and adaptation over time. Given that 5 to 7 are more focused on scaling up implementation, objective 2 will likely focus on the first 4 subdomains for analysis to illustrate associated barriers and facilitators of the early phases of implementation. All facilitators and barriers identified will be then subsequently matched to the subconstructs of the TAM, when possible, to understand their impact on global usability.

The content analysis involves 3 phases. In phase 1, preparation, the analyst will attempt to understand the entire data set through immersion in the data. In phase 2, organization, a composite coding matrix will be devised using the NASSS domains and the TAM subconstructs. The data will be coded and categorized using NVivo R1. Finally, in phase 3, reporting, the descriptive content of the categorization will be presented, addressing trustworthiness.

The saved transcripts of users’ conversations with the chatbots may also be submitted for content analysis to describe and better understand the nature of chatbot-participant interactions, such as conversation topic trends.

#### Objective 3: Implementation Assessment

##### Overview

For this objective, we will further assess the implementation outcomes of the MARVIN chatbots after they are deployed to the general population via Messenger. During this stage, the chatbots will be regularly updated to introduce new features or content, and their impact on implementation will be assessed. If the outcomes are negative, the corresponding version of the chatbot will be submitted for continuous improvement and evaluation.

We anticipate that the participation period will be 12 months and could be adjusted according to the health care context. Participants will receive a link to the same sociodemographic questionnaire as before (ie, via REDCap [Research Electronic Data Capture; Vanderbilt University] or Google Forms) directly through the chatbot at entry into the study (Multimedia Appendix 8). They will then be able to send messages to MARVIN whenever they wish. If the chatbot does not receive a message from the participant within a month of its most recent conversation with them, it will proactively ask the participant the following: “It’s been a month since our last conversation. How have you been?” The participant will be able to turn off this inquiry if they so wish.

Every 2 months, participants will receive a link to a questionnaire on implementation outcomes (Appendix 7). The chatbot will also ask 3 open-ended questions to collect information on the participant’s overall experience and suggestions for continuous improvement of the chatbot.

##### Quantitative Data Collection and Analysis

The implementation outcome questionnaire will assess usability, acceptability, and appropriateness using validated instruments. Fidelity and adoption will be summarized using descriptive statistics on chatbot use metadata.

Usability and acceptability will be measured for objective 3 (implementation) as they will be for objective 2 (usability) using the UMUX-Lite and AES questionnaires as both measures are dynamic and vary with experience. Thus, usability and acceptability ratings may be different for each stage of implementation [14,62].

Appropriateness relates to the relevance or compatibility of the innovation to address a particular issue or problem [14]. The compatibility of an IT innovation is the extent to which it is considered consistent with users’ values, needs, and past experiences [63]. The *Compatibility Subscale* is a validated tool that contains 3 items rated on a 7-point Likert scale (range 1-7) to assess how an IT innovation “fits” with the user’s work style [64]. An adapted version will be administered to health care professional participants. A minimum average score of 5.5 is set as the threshold for adequate compatibility. For patient participants, the *Intervention Appropriateness Measure* will be used [65]. It contains 4 items scored on a 5-point scale to assess an innovation’s suitability for a user. A mean score of at least 4 will indicate the appropriateness of the chatbot intervention for the patient population.

Fidelity is the degree to which the intervention is implemented as intended [14]. On the basis of the fidelity measures of digital health intervention implementation identified by Coorey et al [66], we will analyze the following metadata to comprehensively assess the chatbot fidelity: (1) intervention fidelity (the proportion of participants who continue to use the chatbot after a 1-month period), (2) frequency and duration (the monthly frequency with which participants use the chatbot and the average total duration of using the chatbot), (3) messages delivered (the total number of messages that participants interacted with in the chatbot over the course of the study period as well as the average number per participant), and (4) range of messages received (the frequency distribution of different conversational topics triggered by all participants).

Adoption, or uptake, is “the intention, initial decision, or action to try or employ an innovation or evidence-based practice” [14]. It will be measured using the proportion of monthly new users enrolled to all users in the study. The target will be 5% per month given that the median user growth rate for small-scale software services is 4.4% [67,68].

Statistically, a strategy similar to that of objective 2 will be adopted to describe the data. For usability, acceptability, and appropriateness, a Student *t* test will be used to test the null hypothesis that the average score is inferior or equal to the predetermined thresholds.

##### Qualitative Data Collection and Analysis

To better understand the implementation outcomes, participants will be invited to answer three open-ended questions: (1) What did you like most about using MARVIN? (2) What did you dislike about using MARVIN? (3) How would you improve MARVIN?

The content analysis of this material using the same coding matrix as in objective 2 will likely include the last 3 subdomains of the NASSS framework given the implementation stage at objective 3. This will help document and detail barriers to and facilitators of using the chatbots and their associated implementation outcomes as well as identify targets for continuous improvement.

As with objective 2 (usability), the saved transcripts of chatbot conversations may also be submitted for content analysis to characterize and better understand the nature of chatbot-participant interactions.

#### Objective 4: Evaluate the Impact of Different Stakeholder Partnerships

##### Overview

The reporting of patient and stakeholder engagement roles will follow the revised Guidance for Reporting Involvement of Patients and the Public [69].

##### Quantitative Data Collection and Analysis

Quantitative data on patient and stakeholder engagement activities, such as the number and length of actual workshops or focus groups conducted; the number of attendees; and the user requirements, design parameters, and improvement recommendations made by stakeholders during the workshops or focus groups, will be reported to illustrate the impact of patient and stakeholder engagement on the adaptation or development of the MARVIN chatbots.

##### Qualitative Data Collection and Analysis

Upon completion of each objective, focus groups will be conducted with stakeholders involved in the co-construction of the chatbots to identify the participants’ perspectives on target users’ involvement in research. A focus group guide will be developed for each substudy considering its specific setting. These data will be analyzed thematically along with workshop data from objective 1 to determine how potential end users (patient partners and health care professionals) were integrated into the research team, participated in the work, influenced decision-making, and contributed to chatbot adaptation or development.

### Data Management, Confidentiality, and Security

Only data relevant to this study outlined in this protocol will be collected by the research team. A comprehensive overview of the study’s data management strategy is presented in Table 1, including the collection of recruitment and study data. For objectives 1 (development), 2 (usability), and 4 (partnership evaluation), participants’ basic sociodemographic data and contact information will be recorded. For objective 3 (implementation), only participants’ Facebook account names will be gathered alongside their eligibility responses. Participants will be identified using alphanumeric codes. The link between these codes and the participants’ identities will be kept by the research team within a password-protected digital file safeguarded by the MUHC firewall. Access to these records will be exclusive to the research team.

The scope of the study data will include information from the web-based research questionnaire, qualitative data, transcripts of participant conversations with MARVIN, and MARVIN-related metadata. Data required for distinct study objectives through questionnaires will be collected using an appropriate collection tool (eg, Google Forms or REDCap) and subsequently extracted into a password-protected Microsoft Excel (Microsoft Corp) spreadsheet for analysis. Qualitative data, once collected and transcribed, will be password protected and deidentified during analysis. All study data will only be accessible to the research team.

**Figure 7:**
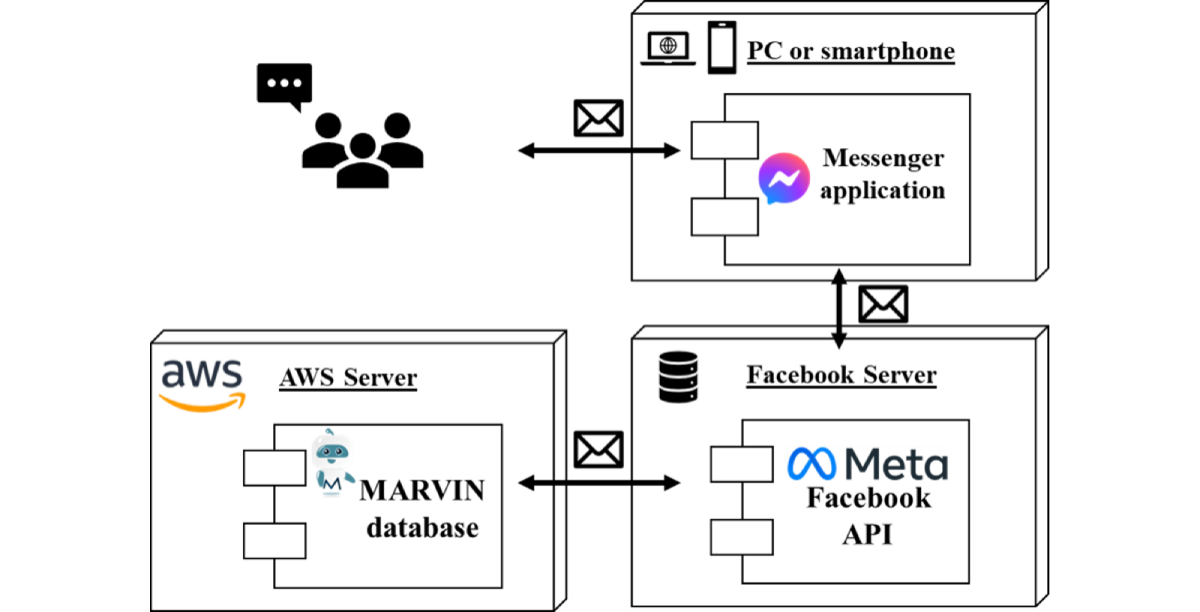
MARVIN chatbot—message data flow diagram. API: application programming interface; AWS: Amazon Web Services.

Regarding the technical cybersecurity aspects of the MARVIN chatbots, Figure 7 offers a visual representation of the data flow as participants engage with the chatbots. Throughout the study, participants will send messages to MARVIN chatbots via their personal devices. These messages will be relayed through Messenger’s application programming interface to the Amazon Web Services (AWS) cloud server, where the research team deployed the MARVIN service. Response messages from MARVIN chatbots will then be sent back to participants’ devices via the Messenger application programming interface. The entire communication process will be encrypted. The research team will be responsible for all of Facebook’s MARVIN chatbot accounts and MARVIN servers deployed on the AWS cloud server. Records of chatbot conversations will be stored on an encrypted AWS cloud server. These data will be anonymized by the research team and used exclusively for future model training to enhance chatbot performance for research and quality assurance purposes.

Conversations on Messenger will also be logged and stored on Messenger’s server, which is necessary for display on both the participant and chatbot interfaces. The data handling in this context adheres to Facebook’s policies [70-72]. If a participant withdraws from the study, the collected study data will be removed as well if the participant so wishes. In particular, in accordance with Messenger’s privacy policy, participants will be asked to delete the conversations with MARVIN from their personal accounts, and the research team will delete the conversations from MARVIN’s account. Thus, Facebook will stop storing these data as they are no longer required to provide their services and Meta Platforms products.

Note that all platforms involved, including Messenger, Google Forms, and REDCap, comply with the General Data Protection Regulation implemented by the European Union. It is recognized as the highest standard available, especially in terms of AI applications, equivalent to or surpassing the Personal Information Protection and Electronic Documents Act in Canada [73], where MARVIN is deployed. Access to each platform’s accounts will be exclusive to the research team and secured through 2-factor authentication.

**Table 1:** Study data management strategy.

		Objective 1	Objective 2	Objective 3	Objective 4
**Recruitment data**					
	Data type	Basic sociodemographic data and contact information	Basic sociodemographic data and contact information	Facebook account name, answers to eligibility questions, and web-based consent records	Basic sociodemographic data and contact information
	Protection method	Password-protected digital files	Password-protected digital files	Encrypted database on Amazon Web Services cloud server and synchronized to password-protected digital files	Password-protected digital files
	Storage location	MUHC^a^ internal storage	MUHC internal storage	Amazon Web Services and MUHC internal storage	MUHC internal storage
**Study data**					
	Data type	Conversation histories	Conversation histories Study questionnaire data Qualitative data	Conversation histories Study questionnaire data	Qualitative data
	Protection method	Encrypted database on Amazon Web Services cloud server and 2-factor–authenticated Facebook MARVIN account	Encrypted database on Amazon Web Services cloud server and 2-factor–authenticated Facebook MARVIN account 2-factor–authenticated Google Forms or REDCap^b^ (Vanderbilt University) accounts and password-protected digital files Password-protected digital files	Encrypted database on Amazon Web Services cloud server and 2-factor–authenticated Facebook MARVIN account 2-factor–authenticated Google Forms or REDCap accounts and password-protected digital files	Password-protected digital files
	Storage location	Amazon Web Services and Facebook	Amazon Web Services and Facebook Google Forms or REDCap and MUHC internal storage MUHC internal storage	Amazon Web Services and Facebook Google Forms or REDCap and MUHC internal storage	MUHC internal storage

^a^MUHC: McGill University Health Centre.

^b^REDCap: Research Electronic Data Capture.

### Governance Board

In view of the current ever shifting regulatory landscape surrounding health care AI applications, a governance board will be formed to help the research team obtain external perspectives; assess the ethical and technological issues that may arise from the MARVIN chatbots; and ensure prudent development, testing, and mitigation of associated risks. The board will also summarize best practices from the substudies to enhance future iterations.

Candidates for the governance board include expert patients as well as experts in the fields of AI, clinical science, ethics, legal affairs, and communications. Invitations will be sent by the research team via email. Annual assemblies will be held for reviewing study progress and exchanging insights. For specific inquiries, members of the governance board will remain reachable by the MARVIN research team via email summons.

### Anticipated Risks and Benefits

Participants in this study will not be exposed to direct physical risk while partaking in focus groups or interviews, conversing with chatbots via messaging, or completing web-based surveys as they will not receive any pharmaceutical or invasive medical interventions. Furthermore, the preceding pilot study did not reveal any known risks of participation.

However, potential indirect risks are worth considering. In the case of web-based recruitment and verbal consent acquisition, there is a risk of confidentiality breach. This risk can be exacerbated if participants use a personal email address to communicate with the research team. In response, researchers will only use institutional email addresses for correspondence purposes. Participants will also be advised to protect their pertinent personal electronic data.

When using the web-based chatbot, participants will use their personal Facebook account and may share details of their participation in the study. Vulnerability to security breaches (eg, device loss, inadvertent device exposure, phishing, and malware) may arise concerning participants’ Facebook accounts. To mitigate this risk, participants will be explicitly reminded during recruitment to secure relevant personal information. The MARVIN chatbots will similarly provide appropriate reminders in the electronic information and consent forms, such as “I recommend that you do not share study-related information with others unnecessarily.”

The time required to complete the questionnaires and participate in interviews or focus groups may be inconvenient and distressing for certain individuals. Others may also be uncomfortable answering specific questions or interacting with the chatbot. In situations in which questions are deemed sensitive, private, or distressing, participants are not obliged to respond. The research team will always be available to discuss participant concerns and refer them to appropriate resources, including teleconsultation with a mental health professional or other support service.

It is possible that the MARVIN chatbots will have difficulty understanding messages from participants during the study. In such instances, the chatbot will indicate that it cannot understand, as described previously in Figure 5, and suggest seeking help from a health care professional. There is also a small chance that the chatbot will provide erroneous advice. As an example, the chatbot might inform a patient who missed a 2-hour dose to stop taking the medication completely and consult a health care professional immediately. To minimize this potential risk, participants will be informed of the limitations of the chatbots during the consent process. In addition, participants will be prompted to report any perceived inaccuracies and their consequences to the research team in a timely manner. Weekly revisions of user chat logs will be conducted by the research team to ensure timely human intervention in the event of errors. The governance board for this study will consistently monitor and discuss these reports.

Finally, any other service-related risks (eg, chatbot or Facebook network service disruptions) will be communicated to each participant in a timely manner and properly documented by the study coordinator.

Participation in the study presents benefits, including early access to chatbot interventions with validated health care information. Participants will also be compensated appropriately upon completion of each study objective [74] in the form of a gift card or, exceptionally, a money transfer.

## Results

From July 2022 to October 2023, four substudies were established in conjunction with the completion of this master protocol:

1. The first study is a continuation of the original MARVIN for HIV self-management. This project has secured funding from the Fonds de recherche du Québec – Santé Réseau SIDA/Maladies Infectieuses (AIDS and Infectious Disease Network). A subprotocol targeting objective 3 (implementation) is currently being prepared and scheduled for REB submission in early 2024. Recruitment is scheduled to begin in mid-2024, and the related data analysis will begin in early 2025.
2. The second study is MARVIN-Pharma, a project to promote community pharmacists’ knowledge of HIV treatment, with its prototype to be completed by the end of 2023. A related manuscript based on a pharmacist needs assessment is in preparation. A subprotocol for objective 2 (usability) is being developed and is scheduled to be submitted for REB approval in early 2024, and recruitment is scheduled for mid-2024.
3. The third project is to develop the MARVINA chatbot for self-management of patients with breast cancer. This study is supported by funding from the MUHC Cedars Cancer Foundation. The subprotocol was approved by the MUHC REB on September 26, 2023 (approval MP-37-2024-9633). Recruitment for objective 1 (development) is expected to begin in early 2024.
4. The fourth study is to develop MARVIN-CHAMP, an accessible chatbot to assist in the management of pediatric patients with infectious conditions. A funding proposal for this project will be submitted to the Canadian Institutes of Health Research Spring 2024 Project Grant program. A subprotocol for objective 1 (development) is under development and scheduled to be submitted for REB approval in mid–October 2023. Recruitment for objective 1 (development) is expected to start in early 2024.

None of the funding sources had any role in the design of this study and will not be involved in the interpretation of the results or the decision to submit them for publication.

## Discussion

### Expected Findings

AI technologies have made phenomenal advances, but relevant clinical translation in key areas remains slow. To the best of our knowledge, this is the first known master protocol in digital health dedicated to implementing chatbot interventions across diverse health conditions and clinical settings. Before this, master protocols had been structured primarily for pharmaceutical intervention studies [75]. This master protocol shares key experimental components and operational processes while capitalizing on the similarities of the underlying IT infrastructures already in place. Coupled subsequently with thorough discussion and deliberation among intended users, developers, administrators, and regulators, the efficiency of creating and coordinating multiple studies of the same type has greatly improved. Although the upfront costs and planning time were significant, with this master protocol taking a year to complete from inception to REB approval, it will facilitate the generation of high-quality evidence essential for guiding medical practice. Through centralized management and shared governance, it also reduces development costs, enables broad decision-making, and allows patients to benefit earlier from advanced interventions.

The selected adaptive platform trial format, although designed initially for oncology and infectious disease drug development, has also been identified as being applicable to digital health interventions [76]. Its flexibility is a noteworthy advantage. Digital health interventions are now increasingly being applied to a wide range of conditions. The flexibility of the infrastructure facilitates the addition of substudies or necessary adjustments to each substudy, and health authorities, institutional review boards, and ethics committees will have a clear understanding of what changes are occurring across substudies [77]. Second, design features such as early termination of the trial or re-estimation of the sample size can avoid wasting resources, thus allowing for faster dissemination of research results to the communities that will benefit the most [38].

Patient and stakeholder contributions are integral to shaping this master protocol and associated materials. Their co-constructive engagement allows them to take a leading role in the ongoing digitization of health care and can help mitigate or even address the risks that chatbots face during implementation, such as those tied to trustworthiness, data privacy, and exacerbating inequalities in access to health care. Incorporating patient and stakeholder involvement strategies in developing master protocols, as suggested by Huml et al [78], can make them more successful for patients, providers, and sponsors. Patients and stakeholders will be invited to contribute on an ongoing basis to subsequent chatbot development, reporting of trial results, product marketing, and knowledge translation. Systematically documenting, investigating, and reporting this entire process will be beneficial in providing the scientific community with a clear and replicable model for responsible AI medical research.

Certain limitations need to be recognized. This master protocol focuses solely on the assessment of chatbot implementation outcomes, similar to master protocols typically prepared for pharmaceutical investigations that focus on “early exploratory development phases” [78]. Clinical and service-related outcomes are not included in this master protocol as there is no consensus on their assessment methods. Notably, <25% of AI-based digital health trials include patient-reported outcome measures as end points despite their widespread use in other health care trials [79]. Therefore, corresponding research efforts are necessary to develop relevant high-quality assessment metrics to foster the development and validation of user-centered chatbots. If the substudy decides to assess their clinical effectiveness, appropriate amendments will be made.

Large language model (LLM) technology is also not considered in this master protocol for the time being. The release of LLMs, represented by GPT-4, has been indeed impressive. Unfortunately, LLM-based chatbots exhibit limitations such as a lack of transparency, sharing of unverified health information, and poor interpretability [80]. These factors threaten the trustworthiness and security of chatbots, which are key to their successful implementation in health care. Although current state-of-the-art LLMs are, of course, embraced to help generate more diverse and personalized responses, trialing these models in clinical settings also remains a challenge owing to the lack of relevant regulatory compliance and the fact that the existing software-as-a-medical-device framework is not suited to such models [81]. Chatbots can be a safe tool to seek information if they are validated and approved by health care professionals and all personal data are properly secured through robust up-to-date privacy and security safeguards [82,83]. In line with this protocol, the team strongly believes in and adheres to a careful content validation and model fine-tuning strategy so as to maximize the accuracy, trustworthiness, and safety of the solutions being delivered for responsible AI innovation.

Finally, another limitation of the protocol is the use of convenience sampling, potentially introducing bias toward individuals more inclined to use digital health technologies. Furthermore, in the web-based direct recruitment process for objective 3 (implementation), all participants will be self-referred, which will make it challenging to assess whether the participants genuinely meet the inclusion criteria. In addition, the self-reported quantitative questionnaire may be susceptible to random participant responses. Outliers in the data will be checked, and appropriate statistical methods will be used to improve the quality of the final data.

### Conclusions

Overall, the development and adaptation of the MARVIN chatbots, co-constructed with those directly involved, hold the promise of fostering patient self-management and enhancing health care efficiency. This study shall provide a comprehensive examination of the implementation outcomes of innovative chatbot interventions tailored for patients and health care professionals. Moreover, it will contribute to the formulation of best practice recommendations for the co-constructive engagement of patients and stakeholders in digital health research. If properly applied, this master protocol has the potential to be sustained for years or even decades and allow innovations to be rapidly translated to clinical practice. Advances in methodology combined with the surge in AI will provide deeper evidence to achieve the goal of patient-partnered personalized medicine and, ultimately, help deliver the right interventions for the right patient at the right time.

## Appendix

Multimedia Appendix 1 CONSORT (Consolidated Standards of Reporting Trials) extension for pilot and feasibility trials checklist.

Multimedia Appendix 2 CONSORT-EHEALTH (Consolidated Standards of Reporting Trials of Electronic and Mobile Health Applications and Online Telehealth) V 1.6.1 checklist.

Multimedia Appendix 3 CONSORT-AI (Consolidated Standards of Reporting Trials–Artificial Intelligence) checklist.

Multimedia Appendix 4 Demonstration video—the MARVIN chatbots study.

Multimedia Appendix 5 Privacy policy—the MARVIN chatbots study.

Multimedia Appendix 6 Information and consent form.

Multimedia Appendix 7 Summary of study procedures for participants.

Multimedia Appendix 8 Study questionnaires and semistructured interview guide for the usability study.

## Abbreviations

AES: Acceptability E-scale
AI: artificial intelligence
AWS: Amazon Web Services
CONSORT: Consolidated Standards of Reporting Trials
CONSORT-AI: Consolidated Standards of Reporting Trials–Artificial Intelligence
CONSORT-EHEALTH: Consolidated Standards of Reporting Trials of Electronic and Mobile Health Applications and Online Telehealth
LLM: large language model
MUHC: McGill University Health Centre
NASSS: nonadoption, abandonment, scale-up, spread, and sustainability
NPS: net promoter score
REB: research ethics board
REDCap: Research Electronic Data Capture
TAM: Technology Acceptance Model
UMUX-Lite: shorter version of the Usability Metric for User Experience

## Data Availability

The data sets generated and analyzed for this study are available from the corresponding author (B Lebouché) upon reasonable request.
