# Supplementary material for: Adapting and Evaluating an AI-Based Chatbot Through Patient and Stakeholder Engagement to Provide Information for Different Health Conditions: Master Protocol for an Adaptive Platform Trial (the MARVIN Chatbots Study)
Source: JMIR Res Protoc. 2024 Feb 13;13:e54668. doi: 10.2196/54668 (PMC10900097; doi:10.2196/54668)
Supplement: Multimedia Appendix 5 [file resprot_v13i1e54668_app5.pdf]

Minimal AntiretRoViral INterference (MARVIN) Privacy Policy

[Following paragraph is the example for MARVIN HIV, to be adapted for other arms of the study.]

The MARVIN chatbot converses with users about HIV self-management, in both English and French. It offers guidance on antiretroviral therapy (ART) medication and ART management when travelling. Further, it provides general HIV-related information to users.

In providing this service, the MARVIN chatbot collects, uses, and discloses information, including personal information, according to the terms of this Privacy Policy. This policy describes the categories of personal information that the MARVIN Chatbot collects, and the purposes for which that information can be collected, used, and disclosed.

1. **Categories of information collected:**

Direct identifiers:

The direct identifiers of users, such as the name associated to their Meta Messenger account, are collected and replaced with a unique alphanumeric code. Direct identifiers are held secure and are only made available to the research team at the Research Institute of the McGill University Health Centre (RI-MUHC) that operates the MARVIN chatbot (hereinafter, the RI-MUHC research team).

Sign-up information:

MARVIN collects basic sign-up information from users to confirm their eligibility to use MARVIN, at the time that these users first send a message to the chatbot through Meta Messenger. This information confirms that users are aged 18 or above, are fluent in English and/or French, and agree to the conditions of use of the MARVIN chatbot, as well as user consent to research participation. This information is stored on an Amazon Web Service (AWS) cloud server, and held in encrypted form. It is also held on the internal storage of the RI-MUHC.

Conversation histories:

MARVIN collects conversation histories from its conversation with users through the Meta Messenger service. These communications are forwarded from Meta Messenger's Application Programming Interface (API) to an Amazon Web Service (AWS) cloud server, and held in an encrypted form. The RI-MUHC research team can access this data through MARVIN chatbot accounts that are two-factor authenticated.

Usage data:

Information about individual and aggregate patterns of chatbot use will be collected and used for multiple purposes. This information will help to assess the performance of the MARVIN chatbot. Statistics on short-term and long-term patterns of use help to assess whether users are experiencing challenges in using the platform, and to gauge success in retaining long-term users and onboarding new users.

2. **How this information is used:**

Information that MARVIN collects can be used for the following purposes:

a. To improve the functioning of MARVIN, related services, and related platforms, and to perform quality assurance.

b. To analyze the performance of MARVIN, related services, and related platforms, including through the generation of aggregate statistics or anonymised information that can be shared with the public or other third parties.

c. To perform research as described in the informed consent materials. The results of research can be shared with the public, including through press releases and academic publications. No personal information will be shared in such public-facing materials.

   It is possible that additional research activities not anticipated at this time will be performed using the information that MARVIN collects. These additional research efforts will be performed according to the contents of the existing informed consent to research participation, to an additional consent gathered through the MARVIN chatbot, or through an ethics waiver of informed consent requirement from a duly constituted Research Ethics Board (REB).

   Further information on the RI-MUHC research team's practices and procedures can be found here: [link to be prepared].

d. To communicate with users of MARVIN, and to better tailor the functioning of MARVIN, related services, and related platforms to the needs of specific users.

e. In certain circumstances, it might be required for MARVIN to use information to ensure compliance with legal obligations, such as information retention requirements, or court orders.

f. To ensure the security and appropriate functioning of the MARVIN chatbot, and to detect and prevent misuse of MARVIN, related services, and related platforms. This includes but is not limited to the implementation of proactive and reactive cybersecurity measures and audits directed to patterns of user behavior that are suggestive of platform misuse.

3. User rights in information

Applicable law, including but not limited to Canadian privacy and data protection legislation, may provide individuals with rights to access information about them, to request that their information be corrected, or to request the destruction or the withdrawal of the personal information that has been collected about them. In Quebec, the location of the Research Institute of the McGill University Health Centre, and the RI-MUHC servers that host the collected data, such rights are guaranteed in both the *Act Respecting Health and Social Services*, and the *Act Respecting Access to Documents Held by Public Bodies and the Protection of Personal Information*.

However, it might not be possible honor requests that pertain to information collected through MARVIN that has been irreversibly de-linked from individual Meta Messenger account identifiers, or that has otherwise been anonymised.

To make a request regarding the rights in information collected through MARVIN, please send us a request at this email address: marvin.cusm@gmail.com.

In addition, research participants using MARVIN can withdraw from research participation according to the procedures established in the informed consent materials provided to them at the time of study enrollment.

4. Disclosure of information

Information that MARVIN collects will not be disclosed to third parties except as provided for in this Privacy Policy.

Identifiable personal information can be shared with third-party service providers that help operate MARVIN, deliver related services, or implement related platforms. These service providers include, but are not limited to, cloud service providers, suppliers of software or analysis services, and trusted partners that provide cybersecurity services and related products. In all circumstances, third party service providers are contractually required to respect the conditions elaborated in this privacy policy, as well as the legal requirements applicable to them and to the RI-MUHC research team.

Research data, including identifiable personal information, can be shared with other teams of researchers according to the conditions established in the informed consent materials. It is possible that research data could be shared according to broader conditions if a more expansive research consent, or an ethics waiver of informed consent, were obtained in the future.

In certain circumstances, it might be required for the RI-MUHC to disclose information to ensure compliance with legal obligations, such as information retention requirements, or court orders.

The RI-MUHC research team may further disclose aggregated or anonymised information to the public or to third parties as part of press-releases, statistical information regarding platform use, or other communications.

5. Links to other websites and affiliate services

In using MARVIN, it is possible that users will be prompted to access and use the websites of affiliate services, or otherwise be directed to external webpages, such as Google Forms, REDCap, and others.

Affiliated services and webpages external to MARVIN are governed by their own Terms of Use and Privacy Policies. The conditions of use and privacy guarantees that such websites provide might differ from those that the RI-MUHC research team guarantees in the MARVIN Privacy Policy.

The MARVIN chatbot provides links to external, third-party websites and affiliate services to enhance the experience of users. This should not be construed as an endorsement of such websites and services. The RI-MUHC research team is not accountable for the collection, use, and disclosure of information on third-party websites. Users must assess the Privacy Policies and Terms of Use on external websites and affiliate services and decide whether or not to use them.

6. Conditions of platform use – Meta Platforms

The MARVIN chatbot and the RI-MUHC research team leverages the Meta Messenger app to communicate with users and collect information. The Terms and Conditions and Privacy Policy of the Meta Messenger app can be consulted through its website and on the app. The RI-MUHC research team does not determine the conditions according to which the Meta Messenger app uses identifiable personal information, nor does it hold itself accountable for such information use.

7. Security

The RI-MUHC research team, the RI-MUHC, and third-party service providers use state-of-the-art security measures in storing and communicating data. This includes in storing data on AWS cloud servers and on RI-MUHC servers. This includes the imposition of organisational, physical, and technological safeguards

in compliance with the requirements set out in the *Act Respecting Health and Social Services*, the *Act Respecting Access to Documents Held by Public Bodies and the Protection of Personal Information*, and other applicable laws and norms.

8. Changes to the Privacy Policy

The MARVIN Privacy Policy might be subject to change in the future. Changes to the privacy policy are made available here and can be consulted at any time.

If changes to the conditions of information use that are described in the informed consent materials, research protocol, and privacy policy are anticipated, the Research Ethics Board and other relevant RI-MUHC personnel will be consulted prior to the implementation of such changes.
