# Supplementary material for: Adapting and Evaluating an AI-Based Chatbot Through Patient and Stakeholder Engagement to Provide Information for Different Health Conditions: Master Protocol for an Adaptive Platform Trial (the MARVIN Chatbots Study)
Source: JMIR Res Protoc. 2024 Feb 13;13:e54668. doi: 10.2196/54668 (PMC10900097; doi:10.2196/54668)
Supplement: Multimedia Appendix 6 [file resprot_v13i1e54668_app6.pdf]

ICF Objective 1 - Model (English and French versions)

[Logo of the local institution]

INFORMED CONSENT FORM

**Research Study Title:**	[Title of the study's arm]<br>Developing or adapting the MARVIN chatbot
**Protocol number:**	[Local REB Protocol number]
**Researcher responsible for the research study:**	Bertrand Lebouché MD, PhD,<br>Center for Outcomes Research and Evaluation (CORE), Research Institute, McGill University Health Center (RI-MUHC)<br>Department of Family Medicine, Faculty of Medicine and Health Sciences, McGill University<br>Chronic Viral Illness Service (CVIS), Royal Victoria Hospital/MUHC- Glen site<br>1001 Decarie Blvd, Room D02.4017<br>Montreal, Quebec, H4A 3J1, Canada<br>bertrand.lebouche@mcgill.ca
**Co-Investigators/sites:**	Sofiane Achiche, Ph.D.<br>Department of Mechanical Engineering, Polytechnique Montréal<br><br>Yuanchao Ma, M.Sc.A<br>CORE, RI-MUHC<br>Department of Mechanical Engineering, Polytechnique Montréal<br><br>Rachel Therrien, M.Sc. Pharmacist<br>University of Montréal Hospital Centre (CHUM)<br><br>Marie-Pascale Pomey, M.D., Ph.D.<br>Research Centre of the University of Montréal Hospital Centre (CRCHUM)<br>Centre of Excellence on Partnership with Patients and the Public (CEPPP)<br>Department of Health Policy, Management and Evaluation, School of Public Health, University of Montreal<br><br>Esli Osmanlliu, M.D., M.Sc.

Department of Pediatrics, Montreal Children's Hospital, MUHC
CORE RI-MUHC

Kim Engler, Ph.D.
CORE RI-MUHC

Serge Vicente, Ph.D.
Department of Family Medicine, Faculty of Medicine and Health Sciences, McGill University
Department of Mathematics and Statistics, University of Montreal

Benoît Lemire, M.Sc. Pharmacist
CVIS-MUHC

David Lessard, Ph.D.
CORE RI-MUHC

Jamil Asselah, M.D.
Department of Oncology, Royal Victoria Hospital/MUHC

Sponsor: Research Institute of the McGill University Health Centre

Funding: FRSQ Sida Maladies Infectieuses
CIHR SPOR mentorship Chair in innovative clinical trials in HIV care
Unité de soutien SSA Québec
Cedar Cancer Foundation

INTRODUCTION

We are inviting you to take part in this research study because you are identified as a key stakeholder for the MARVIN chatbots project.

Before you accept to take part in this study and sign this Informed consent form, please take the time to read, understand and carefully examine the following information.

This form may contain words that you do not understand. We encourage you to ask any questions you may have of the researcher in charge of the project or of other staff involved in the research project and to ask them to explain any words or information that is not clear.

BACKGROUND

This study is part of a master protocol that aims to adapt and evaluate the MARVIN chatbot to different healthcare contexts. Chatbots are software applications that interact with users by simulating a human conversation through text or voice via smartphones or computers. Often harnessing the power of

artificial intelligence to enable natural language interpretation as well as aid decision-making, chatbots can constitute a safe tool for patients to seek verified information. The MARVIN chatbot was initially developed for people living with HIV (PWH) by healthcare providers, engineers, researchers, and people with HIV at the McGill University Health Centre. It is deployed on Meta Messenger (Facebook) and is available any time in both English and French. MARVIN is trained to converse with PWH on the following self-management aspects: 1) guidance for antiretroviral therapy (ART) medication (in regard to time management, dosing, interactions, medication reminders, etc.), 2) ART management when traveling, and 3) common HIV-related knowledge (e.g., symptoms, modes of transmission and prevention, vaccination recommendation, etc.). A pilot study was done to evaluate the global usability of MARVIN among PWH, and users reported that MARVIN was tailored to their needs and was easy to use. The success of MARVIN with PWH deepens the interest of developing other chatbots for different health conditions and for patients or healthcare professionals.

PURPOSE OF THE RESEARCH STUDY

The purpose of this study is to adapt the MARVIN chatbot for [arm's population]. The development process will stay similar to MARVIN, which includes three steps:

1. the users' needs assessment;
2. the creation of a question bank; and
3. the development of corresponding answers.

DESCRIPTION OF THE RESEARCH PROCEDURES

This research study will take place at the [Glen site of the McGill University Health Centre and/or Centre hospitalier de l'Université de Montréal].

1. Duration

Your participation in this research study can last up to 24 months.

2. Study Procedures

It is possible to participate in one or multiple steps:

<u>Step 1- Needs assessment</u>

Before developing the new chatbot, we need to evaluate if the populations under study believe they need such tools. Thus, if you participate in Step 1, you will partake in a focus group, interview and/or need an assessment questionnaire to assess the possible areas of utilization of the chatbot, the expectations, the subjects that could be relevant to integrate, etc. The focus groups and interviews will be led by a trained interviewer, will have a maximum duration of 2 hours, and will be recorded so that it can be transcribed. Your identity will be protected during these interviews. In case of an online focus group/interview, you can choose to show your name and/or your video image on the screen.

<u>Step 2 - Creation of a question bank</u>

For the chatbot to understand the questions asked by the user, and because the same question may be formulated in hundreds of ways, this requires us to collect a large number of different formulations of questions. If you participate in Step 2, you will be part of a design committee to create the questions. Three virtual meetings of 2 hours each are planned for this step. If there are additional activities, you will be contacted in advance via email/phone call. All activities will be recorded so that they can be

transcribed. Your identity will be protected during these interviews. In case of an online focus group/interview, you can choose to show your name and/or your video image on the screen.

Step 3 - Development of corresponding answers

A corpus of qualified and credible answers is another important component of this knowledge base to be created. If you participate in this step, you will be part of the design committee along with experts (e.g. physicians, pharmacists, etc.) to guarantee that the chatbot responds with the appropriate output. The challenge is to provide users with information that is easy to understand and sufficiently colloquial while maintaining its professionalism. Three virtual meetings of 2 hours each are planned for this step. If there are additional activities, you will be contacted in advance via email/phone call. All activities will be recorded so that they can be transcribed. Your identity will be protected during these interviews. In case of an online focus group/interview, you can choose to show your name and/or your video image on the screen. Participants could be asked to test a preliminary version of the chatbot on Meta Messenger in addition to the previously mentioned activities. Participants have the right to opt out of this pre-test.

ELIGIBILITY CRITERIA

To be eligible to participate, you must meet the following criteria:

(1) being 18 years or older
(2) being fluent in English and/or French
(3) being able to understand the requirements of study participation and provide electronic informed consent during the duration of the study
(4) having access to a smartphone, tablet, or computer at home/at workplace
(5) having access to an internet connection at home or data plan on their device

Exclusion criteria include:

(1) not meeting the inclusion criteria
(2) any reason, in the opinion of the investigator, which would make the candidate inappropriate for participation in an investigative study involving a chatbot (e.g., cognitive deficit)

PARTICIPANT'S RESPONSIBILITIES

If you wish to participate in this study, you will be asked to read, sign and date this consent form. To be sure that you are eligible to participate, a member of the research personnel who will verify whether or not you are eligible to participate. This screening could take place in person, by phone or by teleconference.

If you are selected to participate in a focus group, please refrain from discussing content of focus group with non-participants.

BENEFITS ASSOCIATED WITH THE RESEARCH STUDY

There is no direct benefit to you for participating in this research. The information obtained from you could, however, help improve the care of other patients living with [condition/disease] in the future. More specifically, this study could contribute to the use of a new clinical tool for [arm's population].

RISKS ASSOCIATED WITH THE RESEARCH STUDY

We do not foresee any risks associated with this study, and you are not at direct physical risk when participating in focus groups/interviews or conversing with chatbots via text messaging as you are not submitted to any pharmaceutical or invasive medical interventions. In addition, there were no known risks associated with participation in this type of study during the conduct of the pilot study (MUHC REB number: 2021-7191). However, there may be some potential risks.

With online recruitment, there may be risks of breach of confidentiality if you use your personal email address to communicate with a member of the study team. For this reason, researchers will communicate with you using institutional email addresses only. You are also advised to protect relevant personal electronic information.

For online use of the chatbot, you will use your personal Meta accounts to communicate with the chatbot. During use, you may share information about your participation in the study via Meta. There may be potential security breach of your MARVIN Meta account. To protect your personal information on Meta (Facebook), we advise you to 1) protect your login information including your email and your password; 2) log out of your Meta (Facebook) account after use when sharing your device with other people; 3) be vigilant against malicious software and suspicious links, even if they appear to come from a friend or a company you know. Additional security measures could be found at https://www.facebook.com/help/213481848684090 to keep your account secure. The MARVIN chatbots will also provide appropriate reminders (e.g., We recommend that you do not share information related to the study with others in unnecessary circumstances).

The time required to complete the questionnaire, participate in an interview or focus group may be inconvenient and stressful. It is also possible that you feel uncomfortable answering some questions. If you find the information or questions asked to be sensitive, private, or distressing, you do not have to answer those questions. The study team will be available to discuss your concerns and/or to refer you to appropriate resources.

Due to the nature of focus groups, it is impossible to guarantee complete confidentiality as other members of the group will be aware of your identity. However, all participants are instructed to keep what is said in the focus group confidential, as mentioned in the “Participant’s Responsibilities” section.

VOLUNTARY PARTICIPATION AND THE RIGHT TO WITHDRAW

Your participation in this study is voluntary. Therefore, you may refuse to participate. You may also withdraw from the ongoing project at any time, without giving any reason, by informing a member of the study team. Your decision not to participate in the study, or to withdraw from it, will have no impact on the quality of care and services to which you are otherwise entitled, or no bearing on your job or on any work-related evaluations or reports. You will be informed in a timely manner if any information becomes available that may impact your willingness to continue participating in this study.

The researcher or the Research Ethics Board may put an end to your participation without your consent. This may happen if new findings or information indicate that participation is no longer in your interest, if you do not follow study instructions, or if there are administrative reasons to terminate the project.

If you withdraw or are withdrawn from the study, you may also request that the data already collected about you be removed from the study. If the data has been anonymized or was always anonymous (i.e., does not contain any information that can be used to identify you), the data will continue to be used in the analysis of the study.

Nonetheless, conversations with chatbots on Meta can be completely deleted. Inform a member of the research team if this is your wish. The research team will therefore delete all data collected from MARVIN’s account and ask you to do the same on your side. Once both parties have deleted the data, Meta Messenger will also not store it, as it will no longer provide the service.

If the Meta account you have chosen to use to participate in this study is disabled by Meta in accordance with their user policies, you will not be able to continue having access to the chatbot.

CONFIDENTIALITY

During your participation in this study, the doctor in charge of the study and the research team will collect in a study file the information about you needed to meet the scientific objectives of the study.

In addition, participation in this study involves the use of Messenger application provided by Meta (Facebook). The use of Meta Messenger is mandatory to participate in the study. The use of the Meta Messenger involves the sharing of information about you. Part of the collection, processing, storage, and destruction of anonymized data is carried out by the company that provides the Meta Messenger application. This data will likely be saved in a cloud solution located outside of Canada (e.g., United States of America) and could be used by this company for a secondary use, such as business or marketing purposes. The research team, or [Name of the local institution] cannot guarantee the security (confidentiality, integrity, and availability) of this data. Assessing the risk to privacy involved in using the services of Meta is not part of the mandate of the Research Ethics Board of the [Name of the local institution]. You should therefore make sure that you understand the impact using this Meta Messenger will have on your privacy. If you would like more information, please further discuss it with the research team.

All study data collected during this research study (including personal information) will remain confidential to the extent provided by law. You will be identified by a code number only. The key to the code linking your name to your study file will be kept by the doctor in charge of this research study.

All audio-recordings will be transcribed (your words will be written down) in a de-identified fashion (i.e. your name will not appear in the transcripts). The audio-recordings will then be destroyed. It is possible that direct quotes of what you said will be presented in publications and/or conferences. However, precautions will be taken to ensure that it will not be possible to identify you.

The study investigator will use the study information collected about you for research purposes, only to reach the study goals as they are explained in this Information and Consent Document. Your study information will be kept by the investigator in charge of the study for 7 years from the date of publication.

The study information could be printed in medical journals or shared with other people at scientific

meetings, but it will be impossible to identify you.

For auditing purposes, the research study files which could include documents that may identify you may be examined by a person mandated by:

- A representative of the Research Ethics Board who may also contact you to ask about your experience as research participant;
- [Name of the local institution]

All these individuals and organizations will have access to your personal data, but they adhere to a confidentiality policy.

For your safety and to be able to reach you quickly, your family name, first name, coordinates and the date you started and ended the study will be kept for one year after the study ends in a separate list kept by the investigator in charge of the study or by the [Name of the local institution].

You have the right to look at your study file to check the information gathered about you and to correct it, if necessary, as long as the study investigator or [Name of the local institution] keeps this information. However, you may only have access to certain information once the study has ended.

FUNDING OF THE RESEARCH PROJECT

This study will be led by Dr. Bertrand Lebouché and conducted with internal funding. Be aware that study site doctors receive no direct financial compensation for enrolling you in this study.

CONFLICT OF INTERESTS

The researchers have no conflict of interest to declare.

COMPENSATION

For your time participating in this study, you be fairly compensated for each activity at 30$ CAD an hour. If you withdraw from the study (or are withdrawn) before it is completed, compensation will be proportional to the length of your participation.

SHARING STUDY RESULTS

If you wish, you will receive a summary of research results by email.

SHOULD YOU SUFFER ANY HARM

Should you suffer harm of any kind following any procedure related to the research study, you will receive all the care and services required by your state of health.

By agreeing to participate in this research project, you are not waiving any of your rights nor discharging the researcher in charge of the study or the institution, of their civil and professional responsibilities.

CONTACT INFORMATION

If you have questions or if you have a problem you think may be related to your participation in this research study, or if you would like to withdraw, you may communicate with the study doctor [Doctor in charge of the local institution, with their contact information].

For any question concerning your rights as a research participant taking part in this study or if you have comments, or wish to file a complaint, you may communicate with the [Complaints Commissioner of the local institution, with their contact information].

OVERVIEW OF ETHICAL ASPECTS OF THE RESEARCH

The Research Ethics Board of the [Name of the local institution] has given ethics approval to this research study and is responsible for its ongoing ethics oversight at all participating institutions in the health and social services network in Quebec.

DECLARATION OF CONSENT

Research Study Title: [Title of the study's arm]
Developing or adapting the MARVIN chatbot.

I have reviewed the Informed Consent form. Both the research study and the Informed Consent form were explained to me. My questions were answered, and I was given sufficient time to make a decision. After reflection, I consent to participate in this research study in accordance with the conditions stated above, including the use of all personal data collected.

1) I consent to participate in the step 1 (Needs assessment) of the study procedure:

   Yes ☐ No ☐

2) I consent to participate in the design committee (step 2 "Creation of a question bank" and step 3 "Development of corresponding answers"):

   Yes ☐ No ☐

3) I wish to receive a copy of the study results by email.

   Yes ☐ No ☐ If yes, please provide contact information: ___________________________

4) I authorize a member of the research study to communicate with me to see if I am interested in participating in other research studies.

   Yes ☐ No ☐ If yes, please provide contact information: ___________________________

____

Name of participant Signature Date

SIGNATURE OF PERSON OBTAINING CONSENT

I have explained the research study and the terms of this Informed Consent form to the research participant, and I answered all questions asked.

Name of the person obtaining consent	Signature	Date

[Logo de l'institution locale]

FORMULAIRE D’INFORMATION ET DE CONSENTEMENT

Titre de l’étude: [Titre du bras de l’étude]
Développer ou adapter le chatbot MARVIN

Numéro du protocole: [Numéro du protocole local du CER]

Chercheur responsable de l’étude:

Bertrand Lebouché MD, PhD,
Centre de recherche évaluative en santé (CRES)
Institut de recherche du Centre universitaire de santé McGill (IR-CUSM)
Département de médecine de famille, Faculté de médecine et des sciences de la santé, Université McGill
Service des maladies virales chroniques (SMVC), Hôpital Royal Victoria / Site Glen - CUSM
1001 boul. Decarie, salle D02.4017
Montréal, Qu.bec, H4A 3J1, Canada
bertrand.lebouche@mcgill.ca

Co-Investigateurs/sites:

Sofiane Achiche, Ph.D.
Département de génie mécanique, Polytechnique Montréal

Yuanchao Ma, M.Sc.A
CRES, IR-CUSM
Département de génie mécanique, Polytechnique Montréal

Rachel Therrien, M.Sc. Pharmacie
Centre hospitalier de l’Université de Montréal (CHUM)

Marie-Pascale Pomey, M.D., Ph.D.
Centre de recherche du Centre hospitalier de l’Université de Montréal (CRCHUM)
Centre d’excellence sur le partenariat avec les patients et le public (CEPPP)
Département de gestion, d'évaluation et de politique de santé, École de santé publique, Université de Montréal

Esli Osmanlliu, M.D., M.Sc.
Départment de pédiatrie, Hôpital de Montréal pour enfants, CUSM
CRES IR-CUSM

Kim Engler, Ph.D.
CRES IR-CUSM

Serge Vicente, Ph.D.
Département de médecine de famille, Faculté de médecine et des sciences de la santé, Université McGill
Département de mathématiques et statistique, Université de Montréal

Benoît Lemire, M.Sc. Pharmacist
Service des maladies virales chroniques (SMVC), CUSM

David Lessard, Ph.D.
CRES, IR-CUSM

Jamil Asselah, M.D.
Département d'oncologie, Hôpital Royal Victoria/CUSM

Sponsor: Institut de recherche du Centre universitaire de santé McGill

Financement: FRSQ Sida Maladies Infectieuses
IRSC Chaire de mentorat SPORT sur les essais cliniques novateurs dans le traitement du VIH
Unité de soutien SSA Québec
Fondation de cancer Cedar

INTRODUCTION

Nous vous invitons à participer à cette étude de recherche car vous êtes identifié comme une partie prenante clé du projet de chatbots MARVIN.

Avant d'accepter de participer à ce projet et de signer ce formulaire de d'information et de consentement, veuillez prendre le temps de lire, de comprendre et d'examiner attentivement les informations suivantes.

Ce formulaire peut contenir des mots que vous ne comprenez pas. Nous vous encourageons à poser toutes les questions que vous pourriez avoir au chercheur responsable du projet ou à d'autres membres du personnel impliqués dans le projet de recherche et à leur demander d'expliquer des mots ou des informations qui ne sont pas clairs.

CONTEXTE
Cette étude s’inscrit dans un protocole maître qui vise à adapter le chatbot MARVIN à différents contextes de soins de santé et à l’évaluer. Les chatbots sont des applications informatiques qui interagissent avec les utilisateurs en simulant une conversation humaine par messagerie texte ou par la voix via des téléphones intelligents ou des ordinateurs. En exploitant l’intelligence artificielle pour permettre l’interprétation du langage naturel ainsi que pour faciliter la prise de décision, les chatbots peuvent constituer un outil sécuritaire pour que les patients puissent chercher des informations fiables. Le chatbot MARVIN a été initialement développé pour les personnes vivant avec le VIH (PVVIH) par des professionnels de santé, des ingénieurs, des chercheurs et des PVVIH au Centre universitaire de santé McGill. Il est déployé sur Meta Messenger (Facebook) et est disponible à tout moment en anglais et en français. MARVIN est formé pour converser avec les PVVIH sur les aspects d’auto-gestion suivants : 1) des conseils sur la médication d’une thérapie antirétrovirale (ARV) (en ce qui concerne la gestion du temps, le dosage, les interactions, les rappels de médicaments, etc.;) 2) la gestion des ARV lors de voyages; et 3) les connaissances communes liées au VIH (p. ex. symptômes, modes de transmission et de prévention, recommandation de vaccination, etc.). Une étude pilote a été réalisée pour évaluer l’utilisabilité globale de MARVIN parmi les PVVIH, et les utilisateurs ont indiqué que MARVIN était adapté à leurs besoins et était facile à utiliser. Le succès de MARVIN avec PVVIH approfondit l’intérêt de développer d’autres chatbots pour différentes conditions de santé ou pour les patients ou les professionnels de la santé.

OBJECTIFS DE L’ÉTUDE
L’objectif de cette étude est d’adapter le chatbot MARVIN pour [la population du bras de l’étude]. Le processus de développement restera similaire à MARVIN, qui comprend trois étapes :

1. l’évaluation des besoins des utilisateurs;
2. la création d’une banque de questions; et
3. l’élaboration de réponses correspondantes.

DESCRIPTION DES PROCÉDURES DE L’ÉTUDE
Cette étude de recherche se déroulera au [site Glen du Centre universitaire de santé McGill et/ou Centre hospitalier de l'Université de Montréal].

1. Durée
Votre participation à cette étude de recherche peut durer jusqu'à 24 mois.

2. Procédure de l’étude
Il est possible de participer à une ou plusieurs étapes :

<u>Étape 1 - Évaluation des besoins</u>
Avant de développer le nouveau chatbot, nous devons évaluer si la population à l’étude croit avoir besoin de tels outils. Ainsi, si vous participez à l’étape 1, vous participerez à un groupe de discussion, à une entrevue et/ou un questionnaire d’évaluation des besoins pour évaluer les domaines possibles d’utilisation du chatbot, les attentes, les sujets qui pourraient être pertinents à intégrer, etc. Les groupes de discussion et les entrevues seront dirigés par un intervieweur formé, auront une durée maximale de 2 heures et seront enregistrés afin qu’ils puissent être transcrits. Votre identité sera protégée lors de ces entretiens. Dans le cas d’un groupe de discussion ou d’une entrevue en ligne, vous pouvez choisir

d’afficher votre nom et/ou votre image vidéo à l’écran.

Étape 2 - Création d’une banque de question
Pour que le chatbot comprenne les questions posées par l’utilisateur, et parce que la même question peut être formulée d’une centaine de façons, cela nous oblige à collecter un grand nombre de formulations différentes de questions. Si vous participez à l’étape 2, vous ferez partie d’un comité de conception pour créer les questions. Trois réunions virtuelles de 2 heures chacune sont prévues pour cette étape. S’il y a des activités supplémentaires, vous serez contacté à l’avance par courriel / appel téléphonique. Toutes les activités seront enregistrées afin qu’elles puissent être transcrites. Votre identité sera protégée lors de ces entretiens. Dans le cas d’un groupe de discussion ou d’une entrevue en ligne, vous pouvez choisir d’afficher votre nom et/ou votre image vidéo à l’écran.

Étape 3 - Élaboration des réponses correspondantes
Un corpus de réponses qualifiées et crédibles est un autre élément important de cette base de connaissances à créer. Si vous participez à cette étape, vous ferez partie du comité de conception avec des experts (i.e.: médecins, pharmaciens, etc.) pour garantir que le chatbot réponde avec la réponse appropriée. Le défi consiste à fournir aux utilisateurs des informations faciles à comprendre et suffisamment familières tout en conservant leur professionnalisme. Trois réunions virtuelles de 2 heures chacune sont prévues pour cette étape. S’il y a des activités supplémentaires, vous serez contacté à l’avance par courriel / appel téléphonique. Toutes les activités seront enregistrées afin qu’elles puissent être transcrites. Votre identité sera protégée lors de ces entretiens. Dans le cas d’un groupe de discussion ou d’une entrevue en ligne, vous pouvez choisir d’afficher votre nom et/ou votre image vidéo à l’écran. Les participants pourraient être invités à tester une version préliminaire du chatbot sur Meta Messenger en plus des activités mentionnées précédemment. Les participants ont le droit de se retirer de ce pré-test.

CRITÈRES D’ADMISSIBILITÉ
Pour être admissible à participer, vous devez satisfaire aux critères suivants :

(1) être âgé de 18 ans ou plus
(2) parler couramment le français et/ou l’anglais
(3) être en mesure de comprendre les exigences de la participation à l’étude et de fournir un consentement éclairé électronique pendant la durée de l’étude
(4) avoir accès à un téléphone intelligent, une tablette ou un ordinateur à la maison / au travail
(5) avoir accès à une connexion Internet à la maison ou à un forfait de données sur leur appareil

Les critères d’exclusion sont les suivants :

(1) ne pas satisfaire aux critères d’inclusion
(2) toute raison, de l’avis de l’intervieweur, qui rendrait le candidat inapproprié pour participer à une étude d’investigation impliquant un chatbot (par exemple, déficit cognitif)

RESPONSABILITÉS DU PARTICIPANT
Si vous souhaitez participer à cette étude, il vous sera demandé de lire, signer et dater ce formulaire de consentement. Pour être sûr que vous êtes éligible à participer, un membre du personnel de recherche qui vérifiera si vous pouvez participer ou non. Cette sélection pourrait avoir lieu en personne, par téléphone ou par téléconférence.

Si vous êtes sélectionné pour participer à un groupe de discussion, veuillez-vous abstenir de discuter du contenu du groupe de discussion avec les non-participants.

AVANTAGES LIÉS À L'ÉTUDE

Vous n'avez aucun avantage direct à participer à cette recherche. L'information obtenue de vous pourrait, cependant, aider à améliorer les soins d'autres patients vivant avec [condition/maladie] à l'avenir. Plus précisément, cette étude pourrait contribuer à l'utilisation d'un nouvel outil clinique pour [la population du bras de l'étude].

RISQUES LIÉS À L'ÉTUDE

Nous ne prévoyons pas de risques associés à cette étude et vous ne courez aucun risque physique direct lorsque vous participez à des groupes de discussion/entretiens ou lorsque vous conversez avec des chatbots par messagerie texte, car vous n'êtes soumis à aucune intervention pharmaceutique ou médicale invasive. En outre, aucun risque connu n'a été associé à la participation à ce type d'étude pendant la réalisation de l'étude pilote (numéro CUSM REB : 2021-7191). Cependant, il peut y avoir certains risques potentiels.

Avec le recrutement en ligne, il peut y avoir des risques de violation de la confidentialité si vous utilisez votre adresse électronique personnelle pour communiquer avec un membre de l'équipe d'étude. Pour cette raison, les chercheurs communiqueront avec vous en utilisant uniquement les adresses électroniques institutionnelles. Il vous est également conseillé de protéger les informations électroniques personnelles pertinentes.

Pour l'utilisation en ligne du chatbot, vous utiliserez vos comptes Meta personnels pour communiquer avec le chatbot. Pendant l'utilisation, vous pourrez partager des informations sur votre participation à l'étude via Meta. Il peut y avoir une violation potentielle de la sécurité de votre compte Meta MARVIN. Pour protéger vos informations personnelles sur Meta (Facebook), nous vous conseillons de 1) protéger vos informations de connexion, y compris votre adresse électronique et votre mot de passe ; 2) vous déconnecter de votre compte Meta (Facebook) après utilisation lorsque vous partagez votre appareil avec d'autres personnes ; 3) être vigilant à l'égard des logiciels malveillants et des liens suspects, même s'ils semblent provenir d'un ami ou d'une entreprise que vous connaissez. D'autres mesures de sécurité peuvent être trouvées sur le site https://www.facebook.com/help/213481848684090 pour sécuriser votre compte. Les chatbots MARVIN fourniront également des rappels appropriés (par exemple, Nous vous recommandons de ne pas partager les informations relatives à l'étude avec d'autres personnes dans des circonstances inutiles).

Le temps nécessaire pour remplir le questionnaire, participer à un entretien ou à un groupe de discussion peut être gênant et stressant. Il est également possible que vous vous sentiez mal à l'aise pour répondre à certaines questions. Si vous trouvez que les informations ou les questions posées sont sensibles, privées ou pénibles, vous n'êtes pas obligé de répondre à ces questions. L'équipe de l'étude sera disponible pour discuter de vos préoccupations et/ou pour vous orienter vers les ressources appropriées.

En raison de la nature des groupes de discussion, il est impossible de garantir une confidentialité totale,

car les autres membres du groupe connaîtront votre identité. Toutefois, tous les participants sont tenus de respecter la confidentialité de ce qui est dit dans le groupe de discussion, comme indiqué dans la section "Responsabilités des participants".

PARTICIPATION VOLONTAIRE ET DROIT DE RETRAIT

Votre participation à cette étude est volontaire. Vous pouvez donc refuser d'y participer. Vous pouvez également vous retirer du projet en cours à tout moment, sans donner de raison, en informant un membre de l'équipe de l'étude. Votre décision de ne pas participer à l'étude ou de vous en retirer n'aura aucune incidence sur la qualité des soins et des services auxquels vous avez droit par ailleurs, ni sur votre emploi ou sur les évaluations ou rapports liés à votre travail. Vous serez informé(e) en temps utile de toute information susceptible d'avoir une incidence sur votre volonté de continuer à participer à cette étude.

Le chercheur ou le comité d'éthique de la recherche peut mettre un terme à votre participation sans votre consentement. Cela peut se produire si de nouvelles découvertes ou informations indiquent que la participation n'est plus dans votre intérêt, si vous ne suivez pas les instructions de l'étude ou s'il existe des raisons administratives de mettre fin au projet.

Si vous vous retirez ou êtes retiré de l'étude, vous pouvez également demander que les données déjà collectées à votre sujet soient retirées de l'étude. Si les données ont été anonymisées ou ont toujours été anonymes (c'est-à-dire qu'elles ne contiennent aucune information permettant de vous identifier), elles continueront à être utilisées dans l'analyse de l'étude.

Néanmoins, les conversations avec les chatbots sur Meta peuvent être complètement supprimées. Informez un membre de l'équipe de recherche si tel est votre souhait. L'équipe de recherche supprimera alors toutes les données recueillies sur le compte de MARVIN et vous demandera de faire de même de votre côté. Une fois que les deux parties auront supprimé les données, Meta Messenger ne les conservera pas non plus, car il ne fournira plus le service.

Si le compte Meta que vous avez choisi d'utiliser pour participer à cette étude est désactivé par Meta conformément à leurs politiques d'utilisation, vous ne pourrez pas continuer à avoir accès au chatbot.

CONFIDENTIALITÉ

Pendant votre participation à cette étude, le médecin responsable de l'étude et l'équipe de recherche recueilleront dans un dossier d'étude les informations vous concernant nécessaires pour atteindre les objectifs scientifiques de l'étude.

En outre, la participation à cette étude implique l'utilisation de l'application Messenger fournie par Meta (Facebook). L'utilisation de Meta Messenger est obligatoire pour participer à l'étude. L'utilisation de Meta Messenger implique le partage d'informations vous concernant. Une partie de la collecte, du traitement, du stockage et de la destruction des données anonymes est effectuée par la société qui fournit l'application Meta Messenger. Ces données seront probablement sauvegardées dans une solution en nuage située à l'extérieur du Canada (par exemple, aux États-Unis d'Amérique) et pourraient être utilisées par cette entreprise pour un usage secondaire, par exemple à des fins commerciales ou de marketing. L'équipe de recherche, ou [Nom de l'institution locale] ne peuvent garantir la sécurité

(confidentialité, intégrité et disponibilité) de ces données. L'évaluation des risques pour la vie privée liés à l'utilisation des services de Meta ne fait pas partie du mandat du Comité d'éthique de la recherche du [Nom de l'institution locale]. Vous devez donc vous assurer que vous comprenez l'impact de l'utilisation du Meta Messenger sur votre vie privée. Si vous souhaitez obtenir de plus amples informations, veuillez en discuter avec l'équipe de recherche.

Toutes les données recueillies au cours de cette étude de recherche (y compris les renseignements personnels) resteront confidentielles dans les limites prévues par la loi. Vous ne serez identifié que par un numéro de code. La clé du code reliant votre nom à votre dossier d'étude sera conservée par le médecin responsable de cette étude de recherche.

Tous les enregistrements audios seront transcrits (vos paroles seront écrites) de manière dépersonnalisée (c'est-à-dire que votre nom n'apparaîtra pas dans les transcriptions). Les enregistrements audios seront ensuite détruits. Il est possible que des citations directes de vos propos soient présentées dans des publications et/ou des conférences. Toutefois, des précautions seront prises pour s'assurer qu'il ne sera pas possible de vous identifier.

L'investigateur de l'étude utilisera les informations recueillies à votre sujet à des fins de recherche, uniquement pour atteindre les objectifs de l'étude tels qu'ils sont expliqués dans le présent document d'information et de consentement. Vos informations d'étude seront conservées par le chercheur responsable de l'étude pendant 7 ans à compter de la date de publication.

Les informations de l'étude pourraient être imprimées dans des revues médicales ou partagées avec d'autres personnes lors de réunions scientifiques, mais il sera impossible de vous identifier.

Pour s'assurer que l'étude est faite correctement; votre dossier d'étude de recherche ainsi que votre dossier médical pourraient être vérifiés par une personne autorisée par:

- Un représentant du comité d'éthique de la recherche qui peut également communiquer avec vous pour vous renseigner sur votre expérience en tant que participant à la recherche;
- [Nom de l'institution locale]

Pour votre sécurité et pour être en mesure de vous joindre rapidement, votre nom de famille, votre prénom, vos coordonnées et la date à laquelle vous avez commencé et terminé l'étude seront conservés pendant un an après la fin de l'étude dans une liste distincte tenue par le chercheur responsable de l'étude ou par [Nom de l'institution locale].

Vous avez le droit de consulter votre dossier d'étude pour vérifier les renseignements recueillis à votre sujet et pour les corriger, au besoin, à condition que le chercheur de l'étude ou [Nom de l'institution locale] conserve ces renseignements. Toutefois, il se peut que vous n'ayez accès à certains renseignements qu'une fois l'étude terminée.

FINANCEMENT DE L'ÉTUDE

Cette étude sera dirigée par le Dr Bertrand Lebouché et réalisée avec un financement interne. Sachez que les médecins du site d'étude ne reçoivent aucune compensation financière directe pour vous inscrire à cette étude.

CONFLITS D'INTÉRÊTS
Les chercheurs n'ont aucun conflit d'intérêt à déclarer.

COMPENSATION
Pour le temps que vous consacrerez à cette étude, vous serez rémunéré équitablement pour chaque activité à raison de 30 $ CAD de l'heure. Si vous vous retirez de l'étude (ou si vous êtes retiré) avant sa fin, la compensation sera proportionnelle à la durée de votre participation.

PARTAGE DES RÉSULTATS DE L'ÉTUDE
Si vous le souhaitez, vous recevrez un résumé des résultats de la recherche par courrier électronique.

SI VOUS SUBISSEZ UNE BLESSURE
Si vous subissez une blessure de quelque nature à la suite d'une procédure liée à l'étude de recherche, vous recevrez tous les soins et services requis par votre état de santé sans aucun coût pour vous.

En acceptant de participer à ce projet de recherche, vous ne renoncez à aucun de vos droits et ne déchargez pas le chercheur chargé de l'étude ou l'institution de leurs responsabilités civiles et professionnelles.

COORDONNÉES DE LA PERSONNE-RESSOURCE
Si vous avez des questions ou si vous avez un problème qui, selon vous, pourrait être lié à votre participation à cette étude de recherche, ou si vous souhaitez vous retirer, vous pouvez communiquer avec le médecin de l'étude [Médecin responsable de l'institution locale, avec ses coordonnées].

Pour toute question concernant vos droits en tant que participant à la recherche participant à cette étude ou si vous avez des commentaires ou souhaitez déposer une plainte, vous pouvez communiquer avec le [Commissaire aux plaintes de l'institution locale, avec ses coordonnées].

CONTRÔLE DES ASPECTS ÉTHIQUE DE L'ÉTUDE
Le Comité d'éthique de la recherche du [Nom de l'institution locale] a donné son approbation éthique à cette étude de recherche et est responsable de sa surveillance éthique continue dans tous les établissements participants du réseau de la santé et des services sociaux du Québec.

DÉCLARATION DE CONSENTEMENT

Titre de l'étude: [Titre du bras de l'étude]
Développer ou adapter le chatbot MARVIN

J'ai examiné le formulaire de consentement éclairé. L'étude de recherche et le formulaire de consentement éclairé m'ont été expliqués. On a répondu à mes questions et on m'a laissé suffisamment de temps pour prendre une décision. Après réflexion, je consens à participer à cette étude de recherche conformément aux conditions énoncées ci-dessus, y compris l'utilisation de toutes les données personnelles recueillies.

1) Je consens à participer à l’étape 1 (Évaluation des besoins) de la procédure d’étude :

Oui ☐ Non ☐

2) Je consens à participer au comité de conception (étape 2 « Création d’une banque de questions » et étape 3 « Développement des réponses correspondantes »):

Oui ☐ Non ☐

3) Je souhaite recevoir une copie des résultats de l'étude par courriel.

Oui ☐ Non ☐ Si oui, veuillez fournir votre adresse courriel : ________________________

4) J’autorise l’équipe d’étude de recherche de cette étude à communiquer directement avec moi pour me demander si je suis intéressé à participer à d’autres recherches:

Oui ☐ Non ☐ Si oui, veuillez fournir votre adresse courriel : ________________________

Nom du participant	Signature	Date

SIGNATURE DE LA PERSONNE QUI A OBTENU LE CONSENTEMENT
Les avantages, les risques et les procédures potentiels associés à cette étude ont été expliqués en détail au participant volontaire et il a eu amplement le temps et l’occasion de poser des questions et de décider de participer ou non à cette étude.

Nom de la personne qui a obtenu le consentement	Signature	Date

ICF Objective 2 - Model (English and French versions)

[Logo of the local institution]

INFORMED CONSENT FORM

**Research Study Title:**	[Title of the study's arm]<br>Usability study
**Protocol number:**	[Local REB Protocol number]
**Researcher responsible for the research study:**	Bertrand Lebouché MD, PhD,<br>Center for Outcomes Research and Evaluation (CORE), Research Institute, McGill University Health Center (RI-MUHC)<br>Department of Family Medicine, Faculty of Medicine and Health Sciences, McGill University<br>Chronic Viral Illness Service (CVIS), Royal Victoria Hospital/MUHC- Glen site<br>1001 Decarie Blvd, Room D02.4017<br>Montreal, Quebec, H4A 3J1, Canada<br>bertrand.lebouche@mcgill.ca
**Co-Investigators/sites:**	Sofiane Achiche, Ph.D.<br>Department of Mechanical Engineering, Polytechnique Montréal<br><br>Yuanchao Ma, M.Sc.A<br>CORE, RI-MUHC<br>Department of Mechanical Engineering, Polytechnique Montréal<br><br>Rachel Therrien, M.Sc. Pharmacist<br>University of Montréal Hospital Centre (CHUM)<br><br>Marie-Pascale Pomey, M.D., Ph.D.<br>Research Centre of the University of Montréal Hospital Centre (CRCHUM)<br>Centre of Excellence on Partnership with Patients and the Public (CEPPP)<br>Department of Health Policy, Management and Evaluation, School of Public Health, University of Montreal<br><br>Esli Osmanlliu, M.D., M.Sc.

Department of Pediatrics, Montreal Children's Hospital, MUHC
CORE RI-MUHC

Kim Engler, Ph.D.
CORE RI-MUHC

Serge Vicente, Ph.D.
Department of Family Medicine, Faculty of Medicine and Health Sciences, McGill University
Department of Mathematics and Statistics, University of Montreal

Benoît Lemire, M.Sc. Pharmacist
CVIS-MUHC

David Lessard, Ph.D.
CORE RI-MUHC

Jamil Asselah, M.D.
Department of Oncology, Royal Victoria Hospital/MUHC

Sponsor: Research Institute of the McGill University Health Centre

Funding: FRSQ Sida Maladies Infectieuses
CIHR SPOR mentorship Chair in innovative clinical trials in HIV care
Unité de soutien SSA Québec
Cedar Cancer Foundation

INTRODUCTION

We are inviting you to take part in this research study because you are identified as a key stakeholder for the MARVIN chatbots project.

Before you accept to take part in this study and sign this Informed consent form, please take the time to read, understand and carefully examine the following information.

This form may contain words that you do not understand. We encourage you to ask any questions you may have of the researcher in charge of the project or of other staff involved in the research project and to ask them to explain any words or information that is not clear.

BACKGROUND

This study is part of a master protocol that aims to adapt and evaluate the MARVIN chatbot to different healthcare contexts. Chatbots are software applications that interact with users by simulating a human conversation through text or voice via smartphones or computers. Often harnessing the power of

artificial intelligence to enable natural language interpretation as well as aid decision-making, chatbots can constitute a safe tool for patients to seek verified information. The MARVIN chatbot was initially developed for people living with HIV (PWH) by healthcare providers, engineers, researchers, and people with HIV at the McGill University Health Centre. It is deployed on Meta Messenger (Facebook) and is available any time in both English and French. MARVIN is trained to converse with PWH on the following self-management aspects: 1) guidance for antiretroviral therapy (ART) medication (in regard to time management, dosing, interactions, medication reminders, etc.), 2) ART management when traveling, and 3) common HIV-related knowledge (e.g., symptoms, modes of transmission and prevention, vaccination recommendation, etc.). A pilot study was done to evaluate the global usability of MARVIN among PWH, and users reported that MARVIN was tailored to their needs and was easy to use. The success of MARVIN with PWH deepens the interest of developing other chatbots for different health conditions and for patients or healthcare professionals.

PURPOSE OF THE RESEARCH STUDY

Another chatbot has now been developed for [arm’s population]. The study objective is to make a usability study for this new Chatbot to assess how usable the chatbot is to see if it is worth adding to routine [condition/disease] care. This means learning about whether it is easy to use, useful, and satisfying to use. This usability study includes these following steps:

1. Participate in the screening and consent process
2. Ask the chatbot questions through Meta messenger
3. Complete the sociodemographic questionnaire
4. Complete the usability survey on the chatbot
5. Participate in a 2-hour focus group

DESCRIPTION OF THE RESEARCH PROCEDURES

This research study will take place at the [Glen site of the McGill University Health Centre and/or Centre hospitalier de l’Université de Montréal].

3. Duration and number of visits

Your participation in this research study can last up to 1 month.

4. Study Procedures

The entire study participation lasts 4 weeks. After consenting to participate, you will receive training to use the chatbot. Afterwards, you will be able to contact a member of the team if you need help to use the chatbot or if you have questions. See Table 1 for an overview of the study procedures.

<u>Week one</u>

You will complete a sociodemographic questionnaire online (time required: 10 minutes). It will ask you about your age, preferred language, gender/sex, sexual orientation, ethnicity, income, and education level. It will also ask about your use of mobile devices, health apps and Meta Messenger.

<u>Weeks 1 to week 3</u>

You will complete your testing of the chatbot by asking your own questions on three separate topics: [Following is the example for MARVIN HIV, to be adapted for other arms of the study]

- 10 questions and related conversations on medication (time management, difficulties with pill recognition, requirements to take medications with or without food, etc.)
- 5 questions/conversations on medication management when traveling
- 5 questions/conversations on vaccination recommendations

Once you have completed asking your 20 questions, you will then complete the study questionnaire online (that is, the usability survey). This will take about 15 minutes.

Week 4
Randomly selected participants will be invited to take part in a 2-hour semi-structured focus group. This focus group will ask participants about the chatbot's usefulness and ease of use. They will also ask about your satisfaction with it and your intention to use it in the future. Three different focus groups will be conducted with 5 participants each. These interviews will be led by a trained interviewer as a teleconference that you can connect to by the Internet or by telephone. It will be recorded so that it can be transcribed. Your identity will be protected during these interviews. In case of an online focus group/interview, you can choose to show your name and/or your video image on the screen.

Table 1. Summary of study procedures for participants.

**Study procedure**	**Facebook (Meta)**	**At entry**	**Week 1**	**Week 2**	**Week 3**	**Week 4**
Participate in the screening and consent process		X				
Ask the chatbot questions through Meta-messenger	X	X	X	X	X	
Complete the sociodemographic questionnaire		X				
Complete the usability survey on the chatbot					X	
Participate in a 2-hour focus group						X

ELIGIBILITY CRITERIA

To be eligible to participate, you must meet the following criteria:

(1) being 18 years or older
(2) being fluent in English and/or French
(3) being able to understand the requirements of study participation and provide electronic informed consent during the duration of the study
(4) having access to a smartphone, tablet, or computer at home/at workplace
(5) having access to an internet connection at home or data plan on their device
(6) accept to use a Meta Messenger-based Chatbot
(7) accept to use or create a personal Meta account
(8) accept Meta's privacy and data security policies

Exclusion criteria include:

(1) not meeting the inclusion criteria
(2) any reason, in the opinion of the investigator, which would make the candidate inappropriate

for participation in an investigative study involving a chatbot (e.g., cognitive deficit)

PARTICIPANT'S RESPONSIBILITIES

If you wish to participate in this study, you will be asked to read, sign and date this consent form. To be sure that you are eligible to participate, a member of the research personnel who will verify whether or not you are eligible to participate. This screening could take place in person, by phone or by teleconference.

If you are selected to participate in a focus group, please refrain from discussing content of focus group with non-participants.

BENEFITS ASSOCIATED WITH THE RESEARCH STUDY

There is no direct benefit to you for participating in this research. The information obtained from you could, however, help improve the care of other patients living with [condition/disease] in the future. More specifically, this study could contribute to the use of a new clinical tool for [arm's population].

RISKS ASSOCIATED WITH THE RESEARCH STUDY

We do not foresee any risks associated with this study and you are not at direct physical risk when participating in focus groups/interviews or conversing with chatbots via text messaging as you are not submitted to any pharmaceutical or invasive medical interventions. In addition, there were no known risks associated with participation in this type of study during the conduct of the pilot study (MUHC REB number: 2021-7191). However, there may be some potential risks.

With online recruitment, there may be risks of breach of confidentiality if you use your personal email address to communicate with a member of the study team. For this reason, researchers will communicate with you using institutional email addresses only. You are also advised to protect relevant personal electronic information.

For online use of the chatbot, you will use your personal Meta accounts to communicate with the chatbot. During use, you may share information about your participation in the study via Meta. There may be potential security breach of your MARVIN Meta account. To protect your personal information on Meta (Facebook), we advise you to 1) protect your login information including your email and your password; 2) log out of your Meta (Facebook) account after use when sharing your device with other people; 3) be vigilant against malicious software and suspicious links, even if they appear to come from a friend or a company you know. Additional security measures could be found at https://www.facebook.com/help/213481848684090 to keep your account secure. The MARVIN chatbots will also provide appropriate reminders (e.g., We recommend that you do not share information related to the study with others in unnecessary circumstances).

The time required to complete the questionnaire, participate in an interview or focus group may be inconvenient and stressful. It is also possible that you feel uncomfortable answering some questions. If you find the information or questions asked to be sensitive, private, or distressing, you do not have to answer those questions. The study team will be available to discuss your concerns and/or to refer you to appropriate resources.

It may be possible that the MARVIN chatbots do not know what to answer when they cannot understand the messages you send during the study. In such cases, the chatbots will respond that it cannot understand and suggest you seek help from a healthcare professional. Furthermore, it is also possible that the chatbots answer with erroneous advice. For example, the chatbot informed a patient who had missed two hours of medication to stop taking the medication completely. To mitigate this potential risk, you will be explained of the limits of the chatbots during the consent process and prior its use. You will also be reminded to report to the study team all messages you receive that you believe to be incorrect and the impact they have had. These events will be monitored on an ongoing basis and discussed regularly by the governance committee of this study.

Due to the nature of focus groups, it is impossible to guarantee complete confidentiality as other members of the group will be aware of your identity. However, all participants are instructed to keep what is said in the focus group confidential, as mentioned in the “Participant’s Responsibilities” section.

VOLUNTARY PARTICIPATION AND THE RIGHT TO WITHDRAW

Your participation in this study is voluntary. Therefore, you may refuse to participate. You may also withdraw from the ongoing project at any time, without giving any reason, by informing a member of the study team. Your decision not to participate in the study, or to withdraw from it, will have no impact on the quality of care and services to which you are otherwise entitled, or no bearing on your job or on any work-related evaluations or reports. You will be informed in a timely manner if any information becomes available that may impact your willingness to continue participating in this study.

The researcher or the Research Ethics Board may put an end to your participation without your consent. This may happen if new findings or information indicate that participation is no longer in your interest, if you do not follow study instructions, or if there are administrative reasons to terminate the project.

If you withdraw or are withdrawn from the study, you may also request that the data already collected about you be removed from the study. If the data has been anonymized or was always anonymous (i.e., does not contain any information that can be used to identify you), the data will continue to be used in the analysis of the study.

Nonetheless, conversations with chatbots on Meta can be completely deleted. Inform a member of the research team if this is your wish. The research team will therefore delete all data collected from MARVIN’s account and ask you to do the same on your side. Once both parties have deleted the data, Meta Messenger will also not store it, as it will no longer provide the service.

If the Meta account you have chosen to use to participate in this study is disabled by Meta in accordance with their user policies, you will not be able to continue having access to the chatbot.

CONFIDENTIALITY

During your participation in this study, the doctor in charge of the study and the research team will collect in a study file the information about you needed to meet the scientific objectives of the study.

The information will be collected from the sociodemographic questionnaire, the usability survey, and

the conversations you hold with the chatbot on Meta Messenger. Only information necessary for the research study will be collected.

In addition, participation in this study involves the use of Messenger application provided by Meta (Facebook). The use of Meta Messenger is mandatory to participate in the study. The use of the Meta Messenger involves the sharing of information about you. Part of the collection, processing, storage, and destruction of anonymized data is carried out by the company that provides the Meta Messenger application. This data will likely be saved in a cloud solution located outside of Canada (e.g., United States of America) and could be used by this company for a secondary use, such as business or marketing purposes. The research team, or [Name of the local institution] cannot guarantee the security (confidentiality, integrity, and availability) of this data. Assessing the risk to privacy involved in using the services of Meta is not part of the mandate of the Research Ethics Board of the [Name of the local institution]. You should therefore make sure that you understand the impact using this Meta Messenger will have on your privacy. If you would like more information, please further discuss it with the research team.

All study data collected during this research study (including personal information) will remain confidential to the extent provided by law. You will be identified by a code number only. The key to the code linking your name to your study file will be kept by the doctor in charge of this research study.

All audio-recordings will be transcribed (your words will be written down) in a de-identified fashion (i.e. your name will not appear in the transcripts). The audio-recordings will then be destroyed. It is possible that direct quotes of what you said will be presented in publications and/or conferences. However, precautions will be taken to ensure that it will not be possible to identify you.

The study investigator will use the study information collected about you for research purposes, only to reach the study goals as they are explained in this Information and Consent Document. Your study information will be kept by the investigator in charge of the study for 7 years from the date of publication.

The study information could be printed in medical journals or shared with other people at scientific meetings, but it will be impossible to identify you.

For auditing purposes, the research study files which could include documents that may identify you may be examined by a person mandated by:

- A representative of the Research Ethics Board who may also contact you to ask about your experience as research participant;
- [Name of the local institution]

All these individuals and organizations will have access to your personal data, but they adhere to a confidentiality policy.

For your safety and to be able to reach you quickly, your family name, first name, coordinates and the date you started and ended the study will be kept for one year after the study ends in a separate list kept by the investigator in charge of the study or by the [Name of the local institution].

You have the right to look at your study file to check the information gathered about you and to correct it, if necessary, as long as the study investigator or [Name of the local institution] keeps this information. However, you may only have access to certain information once the study has ended.

FUNDING OF THE RESEARCH PROJECT

This study will be led by Dr. Bertrand Lebouché and conducted with internal funding. Be aware that study site doctors receive no direct financial compensation for enrolling you in this study.

CONFLICT OF INTERESTS

The researchers have no conflict of interest to declare.

COMPENSATION

For your time participating in this study, you be compensated at 30$ CAD after you have completed all of the following: the sociodemographic questionnaire, the 20 questions for the chatbot, and the usability survey. If you are invited to participate in the focus group, you will receive additional 30$ CAD compensation after the focus group.

SHARING STUDY RESULTS

If you wish, you will receive a summary of research results by email.

SHOULD YOU SUFFER ANY HARM

Should you suffer harm of any kind following any procedure related to the research study, you will receive all the care and services required by your state of health.

By agreeing to participate in this research project, you are not waiving any of your rights nor discharging the researcher in charge of the study or the institution, of their civil and professional responsibilities.

CONTACT INFORMATION

If you have questions or if you have a problem, you think may be related to your participation in this research study, or if you would like to withdraw, you may communicate with the study doctor [Doctor in charge of the local institution, with their contact information].

For any question concerning your rights as a research participant taking part in this study or if you have comments, or wish to file a complaint, you may communicate with the [Complaints Commissioner of the local institution, with their contact information].

OVERVIEW OF ETHICAL ASPECTS OF THE RESEARCH

The Research Ethics Board of the [Name of the local institution] has given ethics approval to this research study and is responsible for its ongoing ethics oversight at all participating institutions in the health and social services network in Quebec.

DECLARATION OF CONSENT

Research Study Title: [Title of the study's arm]

Developing or adapting the MARVIN chatbot.

I have reviewed the Informed Consent form. Both the research study and the Informed Consent form were explained to me. My questions were answered, and I was given sufficient time to make a decision. After reflection, I consent to participate in this research study in accordance with the conditions stated above, including the use of all personal data collected.

If this is your first participation in the study:

5) I consent to participate in the study:

Yes ☐ No ☐

6) I wish to receive a copy of the study results by email.

Yes ☐ No ☐ If yes, please provide contact information: __________________________

7) I authorize a member of the research study to communicate with me to see if I am interested in participating in other research studies.

Yes ☐ No ☐ If yes, please provide contact information: __________________________

____

Name of participant Signature Date

If you have participated in the prior step of this study (development and adaptation of the MARVIN chatbot), you may give verbal consent to extend your participation:

____

Name of participant Date

____

Name of an impartial witness Signature Date

SIGNATURE OF PERSON OBTAINING CONSENT

I have explained the research study and the terms of this Informed Consent form to the research participant, and I answered all questions asked.

____

Name of the person obtaining consent Signature Date

[Logo de l'institution locale]

FORMULAIRE D'INFORMATION ET DE CONSENTEMENT

Titre de l'étude: [Titre du bras de l'étude]
Étude d'utilisabilité

Numéro du protocole: [Numéro du protocole local du CER]

Chercheur responsable de l'étude:
Bertrand Lebouché MD, PhD,
Centre de recherche évaluative en santé (CRES)
Institut de recherche du Centre universitaire de santé McGill (IR-CUSM)
Département de médecine de famille, Faculté de médecine et des sciences de la santé, Université McGill
Service des maladies virales chroniques (SMVC), Hôpital Royal Victoria / Site Glen - CUSM
1001 boul. Decarie, salle D02.4017
Montréal, Qu.bec, H4A 3J1, Canada
bertrand.lebouche@mcgill.ca

Co-Investigateurs/sites:
Sofiane Achiche, Ph.D.
Département de génie mécanique, Polytechnique Montréal

Yuanchao Ma, M.Sc.A
CRES, IR-CUSM
Département de génie mécanique, Polytechnique Montréal

Rachel Therrien, M.Sc. Pharmacie
Centre hospitalier de l'Université de Montréal (CHUM)

Marie-Pascale Pomey, M.D., Ph.D.
Centre de recherche du Centre hospitalier de l'Université de Montréal (CRCHUM)
Centre d'excellence sur le partenariat avec les patients et le public (CEPPP)
Département de gestion, d'évaluation et de politique de santé, École de santé publique, Université de Montréal

Esli Osmanlliu, M.D., M.Sc.

Départment de pédiatrie, Hôpital de Montréal pour enfants, CUSM
CRES IR-CUSM

Kim Engler, Ph.D.
CRES IR-CUSM

Serge Vicente, Ph.D.
Département de médecine de famille, Faculté de médecine et des sciences de la santé, Université McGill
Département de mathématiques et statistique, Université de Montréal

Benoît Lemire, M.Sc. Pharmacist
Service des maladies virales chroniques (SMVC), CUSM

David Lessard, Ph.D.
CRES, IR-CUSM

Jamil Asselah, M.D.
Département d'oncologie, Hôpital Royal Victoria/CUSM

Sponsor: Institut de recherche du Centre universitaire de santé McGill

Financement: FRSQ Sida Maladies Infectieuses
IRSC Chaire de mentorat SPORT sur les essais cliniques novateurs dans le traitement du VIH
Unité de soutien SSA Québec
Fondation de cancer Cedar

INTRODUCTION

Nous vous invitons à participer à cette étude de recherche car vous êtes identifié comme une partie prenante clé du projet de chatbots MARVIN.

Avant d'accepter de participer à ce projet et de signer ce formulaire de d'information et de consentement, veuillez prendre le temps de lire, de comprendre et d'examiner attentivement les informations suivantes.

Ce formulaire peut contenir des mots que vous ne comprenez pas. Nous vous encourageons à poser toutes les questions que vous pourriez avoir au chercheur responsable du projet ou à d'autres membres du personnel impliqués dans le projet de recherche et à leur demander d'expliquer des mots ou des informations qui ne sont pas clairs.

CONTEXTE

Cette étude s'inscrit dans un protocole maître qui vise à adapter le chatbot MARVIN à différents

contextes de soins de santé et à l'évaluer. Les chatbots sont des applications informatiques qui interagissent avec les utilisateurs en simulant une conversation humaine par messagerie texte ou par la voix via des téléphones intelligents ou des ordinateurs. En exploitant l'intelligence artificielle pour permettre l'interprétation du langage naturel ainsi que pour faciliter la prise de décision, les chatbots peuvent constituer un outil sécuritaire pour que les patients puissent chercher des informations fiables. Le chatbot MARVIN a été initialement développé pour les personnes vivant avec le VIH (PVVIH) par des professionnels de santé, des ingénieurs, des chercheurs et des PVVIH au Centre universitaire de santé McGill. Il est déployé sur Meta Messenger (Facebook) et est disponible à tout moment en anglais et en français. MARVIN est formé pour converser avec les PVVIH sur les aspects d'auto-gestion suivants : 1) des conseils sur la médication d'une thérapie antirétrovirale (ARV) (en ce qui concerne la gestion du temps, le dosage, les interactions, les rappels de médicaments, etc.;) 2) la gestion des ARV lors de voyages; et 3) les connaissances communes liées au VIH (p. ex. symptômes, modes de transmission et de prévention, recommandation de vaccination, etc.). Une étude pilote a été réalisée pour évaluer l'utilisabilité globale de MARVIN parmi les PVVIH, et les utilisateurs ont indiqué que MARVIN était adapté à leurs besoins et était facile à utiliser. Le succès de MARVIN avec PVVIH approfondit l'intérêt de développer d'autres chatbots pour différentes conditions de santé ou pour les patients ou les professionnels de la santé.

OBJECTIFS DE L'ÉTUDE

Un chatbot a maintenant été développé pour [population du bras de l'étude]. L'objectif de l'étude est de réaliser une étude d'utilisabilité pour ce nouveau chatbot afin d'évaluer son utilité et de voir s'il vaut la peine de l'ajouter aux soins de routine de [condition/maladie]. Cela signifie de savoir s'il est facile, utile et satisfaisant à utiliser. Cette étude d'utilisabilité comprend les étapes suivantes :

1. Participer à la procédure de sélection et de consentement
2. Poser des questions au chatbot par le biais de Meta Messenger.
3. Remplir le questionnaire sociodémographique
4. Remplir le questionnaire d'utilisabilité sur le chatbot.
5. Participer à un groupe de discussion de 2 heures

DESCRIPTION DES PROCÉDURES DE L'ÉTUDE

Cette étude de recherche se déroulera au [site Glen du Centre universitaire de santé McGill et/ou Centre hospitalier de l'Université de Montréal].

3. Durée

Votre participation à cette étude de recherche peut durer 1 mois.

4. Procédure de l'étude

La participation à l'étude dure 4 semaines. Après avoir accepté de participer, vous recevrez une formation pour utiliser le chatbot. Ensuite, vous pourrez contacter un membre de l'équipe si vous avez besoin d'aide pour utiliser le chatbot ou si vous avez des questions. Voir le tableau 1 pour un aperçu des procédures de l'étude.

<u>Semaine 1</u>

Vous compléterez un questionnaire sociodémographique en ligne (temps requis : 10 minutes). Il vous interrogera sur votre âge, votre langue préférée, votre genre/sexe, votre orientation sexuelle, votre

origine ethnique, vos revenus et votre niveau d'éducation. On vous interrogera également sur votre utilisation des appareils mobiles, des applications de santé et sur Meta Messenger.

Semaine 1 à 3
Vous compléterez votre test du chatbot en posant vos propres questions sur trois sujets distincts:
[Voici l'exemple pour MARVIN HIV, à adapter en fonction d'autres bras de l'étude.]

- 10 questions et conversations connexes sur les médicaments (gestion du temps, difficultés à reconnaître les pilules, obligation de prendre les médicaments avec ou sans nourriture, etc.)
- 5 questions/conversations sur la gestion des médicaments en voyage
- 5 questions/conversations sur les recommandations de vaccination

Une fois que vous aurez terminé de poser vos 20 questions, vous remplirez le questionnaire de l'étude en ligne (c'est-à-dire l'enquête d'utilisabilité). Cela vous prendra environ 15 minutes.

Semaine 4
Des participants sélectionnés au hasard seront invités à prendre part à un groupe de discussion semi-structuré de deux heures. Ce groupe de discussion interrogera les participants sur l'utilité et la facilité d'utilisation du chatbot. Ils seront également interrogés sur leur satisfaction à son égard et sur leur intention de l'utiliser à l'avenir. Trois groupes de discussion différents seront organisés avec 5 participants chacun. Ces entretiens seront menés par un intervieweur formé sous forme de téléconférence à laquelle vous pourrez vous connecter par Internet ou par téléphone. Il sera enregistré afin de pouvoir être transcrit. Votre identité sera protégée pendant ces entretiens. Dans le cas d’un groupe de discussion ou d’une entrevue en ligne, vous pouvez choisir d’afficher votre nom et/ou votre image vidéo à l’écran.

Tableau 1. Résumé des procédures de l'étude pour les participants.

**Procédures de l’étude**	**Facebook (Meta)**	**Début**	**Semaine 1**	**Semaine 2**	**Semaine 3**	**Semaine 4**
Participer au processus de sélection et de consentement		X				
Posez des questions au chatbot par le biais de Meta-messenger.	X	X	X	X	X	
Remplir le questionnaire sociodémographique		X				
Compléter le questionnaire d’utilisabilité pour le chatbot					X	
Participer à un groupe de discussion de 2 heures						X

CRITÈRES D’ADMISSIBILITÉ

Pour être admissible à participer, vous devez satisfaire aux critères suivants :

(1) être âgé de 18 ans ou plus

(2) parler couramment le français et/ou l'anglais
(3) être en mesure de comprendre les exigences de la participation à l'étude et de fournir un consentement éclairé électronique pendant la durée de l'étude
(4) avoir accès à un téléphone intelligent, une tablette ou un ordinateur à la maison / au travail
(5) avoir accès à une connexion Internet à la maison ou à un forfait de données sur leur appareil
(6) accepter d'utiliser un Chatbot basé sur Meta Messenger
(7) accepter d'utiliser ou de créer un compte personnel Meta
(8) accepter les politiques de confidentialité et de sécurité des données de Meta.

Les critères d'exclusion sont les suivants :
(1) ne pas satisfaire aux critères d'inclusion
(2) toute raison, de l'avis de l'intervieweur, qui rendrait le candidat inapproprié pour participer à une étude d'investigation impliquant un chatbot (par exemple, déficit cognitif)

RESPONSABILITÉS DU PARTICIPANT

Si vous souhaitez participer à cette étude, il vous sera demandé de lire, signer et dater ce formulaire de consentement. Pour être sûr que vous êtes éligible à participer, un membre du personnel de recherche qui vérifiera si vous pouvez participer ou non. Cette sélection pourrait avoir lieu en personne, par téléphone ou par téléconférence.

Si vous êtes sélectionné pour participer à un groupe de discussion, veuillez-vous abstenir de discuter du contenu du groupe de discussion avec les non-participants.

AVANTAGES LIÉS À L'ÉTUDE

Vous n'avez aucun avantage direct à participer à cette recherche. L'information obtenue de vous pourrait, cependant, aider à améliorer les soins d'autres patients vivant avec [condition/maladie] à l'avenir. Plus précisément, cette étude pourrait contribuer à l'utilisation d'un nouvel outil clinique pour [la population du bras de l'étude].

RISQUES LIÉS À L'ÉTUDE

Nous ne prévoyons pas de risques associés à cette étude et vous ne courez aucun risque physique direct lorsque vous participez à des groupes de discussion/entretiens ou lorsque vous conversez avec des chatbots par messagerie texte, car vous n'êtes soumis à aucune intervention pharmaceutique ou médicale invasive. En outre, aucun risque connu n'a été associé à la participation à ce type d'étude pendant la réalisation de l'étude pilote (numéro CUSM REB : 2021-7191). Cependant, il peut y avoir certains risques potentiels.

Avec le recrutement en ligne, il peut y avoir des risques de violation de la confidentialité si vous utilisez votre adresse électronique personnelle pour communiquer avec un membre de l'équipe d'étude. Pour cette raison, les chercheurs communiqueront avec vous en utilisant uniquement les adresses électroniques institutionnelles. Il vous est également conseillé de protéger les informations électroniques personnelles pertinentes.

Pour l'utilisation en ligne du chatbot, vous utiliserez vos comptes Meta personnels pour communiquer avec le chatbot. Pendant l'utilisation, vous pourrez partager des informations sur votre participation à

l'étude via Meta. Il peut y avoir une violation potentielle de la sécurité de votre compte Meta MARVIN. Pour protéger vos informations personnelles sur Meta (Facebook), nous vous conseillons de 1) protéger vos informations de connexion, y compris votre adresse électronique et votre mot de passe ; 2) vous déconnecter de votre compte Meta (Facebook) après utilisation lorsque vous partagez votre appareil avec d'autres personnes ; 3) être vigilant à l'égard des logiciels malveillants et des liens suspects, même s'ils semblent provenir d'un ami ou d'une entreprise que vous connaissez. D'autres mesures de sécurité peuvent être trouvées sur le site https://www.facebook.com/help/213481848684090 pour sécuriser votre compte. Les chatbots MARVIN fourniront également des rappels appropriés (par exemple, Nous vous recommandons de ne pas partager les informations relatives à l'étude avec d'autres personnes dans des circonstances inutiles).

Le temps nécessaire pour remplir le questionnaire, participer à un entretien ou à un groupe de discussion peut être gênant et stressant. Il est également possible que vous vous sentiez mal à l'aise pour répondre à certaines questions. Si vous trouvez que les informations ou les questions posées sont sensibles, privées ou pénibles, vous n'êtes pas obligé de répondre à ces questions. L'équipe de l'étude sera disponible pour discuter de vos préoccupations et/ou pour vous orienter vers les ressources appropriées.

Il est possible que les chatbots MARVIN ne sachent pas quoi répondre lorsqu'ils ne comprennent pas les messages que vous envoyez pendant l'étude. Dans ce cas, les chatbots répondront qu'ils ne peuvent pas comprendre et vous suggéreront de demander l'aide d'un professionnel de la santé. Par ailleurs, il est également possible que les chatbots répondent par des conseils erronés. Par exemple, le chatbot a conseillé à un patient qui avait oublié de prendre son médicament pendant deux heures d'arrêter complètement de le prendre. Pour atténuer ce risque potentiel, les limites des chatbots vous seront expliquées au cours du processus de consentement et avant leur utilisation. Il vous sera également rappelé de signaler à l'équipe de l'étude tous les messages que vous recevez et qui vous semblent incorrects, ainsi que l'impact qu'ils ont eu. Ces événements seront classés comme des événements indésirables pour cette étude. Ces événements feront l'objet d'un suivi permanent et seront discutés régulièrement par le comité de gouvernance de cette étude.

En raison de la nature des groupes de discussion, il est impossible de garantir une confidentialité totale, car les autres membres du groupe connaîtront votre identité. Toutefois, tous les participants sont tenus de respecter la confidentialité de ce qui est dit dans le groupe de discussion, comme indiqué dans la section "Responsabilités des participants".

PARTICIPATION VOLONTAIRE ET DROIT DE RETRAIT

Votre participation à cette étude est volontaire. Vous pouvez donc refuser d'y participer. Vous pouvez également vous retirer du projet en cours à tout moment, sans donner de raison, en informant un membre de l'équipe de l'étude. Votre décision de ne pas participer à l'étude ou de vous en retirer n'aura aucune incidence sur la qualité des soins et des services auxquels vous avez droit par ailleurs, ni sur votre emploi ou sur les évaluations ou rapports liés à votre travail. Vous serez informé(e) en temps utile de toute information susceptible d'avoir une incidence sur votre volonté de continuer à participer à cette étude.

Le chercheur ou le comité d'éthique de la recherche peut mettre un terme à votre participation sans

votre consentement. Cela peut se produire si de nouvelles découvertes ou informations indiquent que la participation n'est plus dans votre intérêt, si vous ne suivez pas les instructions de l'étude ou s'il existe des raisons administratives de mettre fin au projet.

Si vous vous retirez ou êtes retiré de l'étude, vous pouvez également demander que les données déjà collectées à votre sujet soient retirées de l'étude. Si les données ont été anonymisées ou ont toujours été anonymes (c'est-à-dire qu'elles ne contiennent aucune information permettant de vous identifier), elles continueront à être utilisées dans l'analyse de l'étude.

Néanmoins, les conversations avec les chatbots sur Meta peuvent être complètement supprimées. Informez un membre de l'équipe de recherche si tel est votre souhait. L'équipe de recherche supprimera alors toutes les données recueillies sur le compte de MARVIN et vous demandera de faire de même de votre côté. Une fois que les deux parties auront supprimé les données, Meta Messenger ne les conservera pas non plus, car il ne fournira plus le service.

Si le compte Meta que vous avez choisi d'utiliser pour participer à cette étude est désactivé par Meta conformément à leurs politiques d'utilisation, vous ne pourrez pas continuer à avoir accès au chatbot.

CONFIDENTIALITÉ

Pendant votre participation à cette étude, le médecin responsable de l'étude et l'équipe de recherche recueilleront dans un dossier d'étude les informations vous concernant nécessaires pour atteindre les objectifs scientifiques de l'étude.

Les informations seront collectées à partir du questionnaire sociodémographique, du questionnaire d'utilisabilité, et des conversations que vous tenez avec le chatbot sur Meta Messenger. Seules les informations nécessaires à l'étude de recherche seront collectées.

En outre, la participation à cette étude implique l'utilisation de l'application Messenger fournie par Meta (Facebook). L'utilisation de Meta Messenger est obligatoire pour participer à l'étude. L'utilisation de Meta Messenger implique le partage d'informations vous concernant. Une partie de la collecte, du traitement, du stockage et de la destruction des données anonymes est effectuée par la société qui fournit l'application Meta Messenger. Ces données seront probablement sauvegardées dans une solution en nuage située à l'extérieur du Canada (par exemple, aux États-Unis d'Amérique) et pourraient être utilisées par cette entreprise pour un usage secondaire, par exemple à des fins commerciales ou de marketing. L'équipe de recherche, ou [Nom de l'institution locale] ne peuvent garantir la sécurité (confidentialité, intégrité et disponibilité) de ces données. L'évaluation des risques pour la vie privée liés à l'utilisation des services de Meta ne fait pas partie du mandat du Comité d'éthique de la recherche du [Nom de l'institution locale]. Vous devez donc vous assurer que vous comprenez l'impact de l'utilisation du Meta Messenger sur votre vie privée. Si vous souhaitez obtenir de plus amples informations, veuillez en discuter avec l'équipe de recherche.

Toutes les données recueillies au cours de cette étude de recherche (y compris les renseignements personnels) resteront confidentielles dans les limites prévues par la loi. Vous ne serez identifié que par un numéro de code. La clé du code reliant votre nom à votre dossier d'étude sera conservée par le médecin responsable de cette étude de recherche.

Tous les enregistrements audios seront transcrits (vos paroles seront écrites) de manière dépersonnalisée (c'est-à-dire que votre nom n'apparaîtra pas dans les transcriptions). Les enregistrements audios seront ensuite détruits. Il est possible que des citations directes de vos propos soient présentées dans des publications et/ou des conférences. Toutefois, des précautions seront prises pour s'assurer qu'il ne sera pas possible de vous identifier.

L'investigateur de l'étude utilisera les informations recueillies à votre sujet à des fins de recherche, uniquement pour atteindre les objectifs de l'étude tels qu'ils sont expliqués dans le présent document d'information et de consentement. Vos informations d'étude seront conservées par le chercheur responsable de l'étude pendant 7 ans à compter de la date de publication.

Les informations de l'étude pourraient être imprimées dans des revues médicales ou partagées avec d'autres personnes lors de réunions scientifiques, mais il sera impossible de vous identifier.

Pour s'assurer que l'étude est faite correctement; votre dossier d'étude de recherche ainsi que votre dossier médical pourraient être vérifiés par une personne autorisée par:

- Un représentant du comité d'éthique de la recherche qui peut également communiquer avec vous pour vous renseigner sur votre expérience en tant que participant à la recherche;
- [Nom de l'institution locale]

Pour votre sécurité et pour être en mesure de vous joindre rapidement, votre nom de famille, votre prénom, vos coordonnées et la date à laquelle vous avez commencé et terminé l'étude seront conservés pendant un an après la fin de l'étude dans une liste distincte tenue par le chercheur responsable de l'étude ou par [Nom de l'institution locale].

Vous avez le droit de consulter votre dossier d'étude pour vérifier les renseignements recueillis à votre sujet et pour les corriger, au besoin, à condition que le chercheur de l'étude ou [Nom de l'institution locale] conserve ces renseignements. Toutefois, il se peut que vous n'ayez accès à certains renseignements qu'une fois l'étude terminée.

FINANCEMENT DE L'ÉTUDE

Cette étude sera dirigée par le Dr Bertrand Lebouché et réalisée avec un financement interne. Sachez que les médecins du site d'étude ne reçoivent aucune compensation financière directe pour vous inscrire à cette étude.

CONFLITS D'INTÉRÊTS

Les chercheurs n'ont aucun conflit d'intérêt à déclarer.

COMPENSATION

Pour le temps que vous consacrerez à cette étude, vous recevrez une compensation de 30$ CAD après avoir complété ce qui suit : le questionnaire sociodémographique, les 20 questions pour le chatbot et le questionnaire d'utilisation. Si vous êtes invité à participer au groupe de discussion, vous recevrez une compensation additionnelle de 30$ CAD après le groupe de discussion.

PARTAGE DES RÉSULTATS DE L'ÉTUDE
Si vous le souhaitez, vous recevrez un résumé des résultats de la recherche par courrier électronique.

SI VOUS SUBISSEZ UNE BLESSURE
Si vous subissez une blessure de quelque nature à la suite d'une procédure liée à l'étude de recherche, vous recevrez tous les soins et services requis par votre état de santé sans aucun coût pour vous.

En acceptant de participer à ce projet de recherche, vous ne renoncez à aucun de vos droits et ne déchargez pas le chercheur chargé de l'étude ou l'institution de leurs responsabilités civiles et professionnelles.

COORDONNÉES DE LA PERSONNE-RESSOURCE
Si vous avez des questions ou si vous avez un problème qui, selon vous, pourrait être lié à votre participation à cette étude de recherche, ou si vous souhaitez vous retirer, vous pouvez communiquer avec le médecin de l'étude [Médecin responsable de l'institution locale, avec ses coordonnées].

Pour toute question concernant vos droits en tant que participant à la recherche participant à cette étude ou si vous avez des commentaires ou souhaitez déposer une plainte, vous pouvez communiquer avec le [Commissaire aux plaintes de l'institution locale, avec ses coordonnées].

CONTRÔLE DES ASPECTS ÉTHIQUE DE L'ÉTUDE
Le Comité d'éthique de la recherche du [Nom de l'institution locale] a donné son approbation éthique à cette étude de recherche et est responsable de sa surveillance éthique continue dans tous les établissements participants du réseau de la santé et des services sociaux du Québec.

DÉCLARATION DE CONSENTEMENT

Titre de l'étude: [Titre du bras de l'étude]
Étude d'utilisabilité

J'ai examiné le formulaire de consentement éclairé. L'étude de recherche et le formulaire de consentement éclairé m'ont été expliqués. On a répondu à mes questions et on m'a laissé suffisamment de temps pour prendre une décision. Après réflexion, je consens à participer à cette étude de recherche conformément aux conditions énoncées ci-dessus, y compris l'utilisation de toutes les données personnelles recueillies.

<u>Si c'est votre première participation à l'étude :</u>
5) Je consens à participer à l'étude :

Oui ☐ Non ☐

6) Je souhaite recevoir une copie des résultats de l'étude par courriel.

Oui ☐ Non ☐ Si oui, veuillez fournir votre adresse courriel : ________________________

7) J'autorise l'équipe d'étude de recherche de cette étude à communiquer directement avec moi pour me demander si je suis intéressé à participer à d'autres recherches:

Oui ☐ Non ☐ Si oui, veuillez fournir votre adresse courriel : ________________________

Nom du participant	Signature	Date

<u>Si vous avez participé à l'étape précédente de cette étude (développement et adaptation du chatbot MARVIN), vous pouvez donner votre consentement verbal pour prolonger votre participation:</u>

Nom du participant		Date

Nom d'un témoin impartial	Signature	Date

SIGNATURE DE LA PERSONNE QUI A OBTENU LE CONSENTEMENT

Les avantages, les risques et les procédures potentiels associés à cette étude ont été expliqués en détail au participant volontaire et il a eu amplement le temps et l'occasion de poser des questions et de décider de participer ou non à cette étude.

Nom de la personne qui a obtenu le consentement	Signature	Date

ICF Objective 3 - Model (English and French versions)

[Logo of the local institution]

INFORMED CONSENT FORM

**Research Study Title:**	[Title of the study's arm]<br>Implementation study
**Protocol number:**	[Local REB Protocol number]
**Researcher responsible for the research study:**	Bertrand Lebouché MD, PhD,<br>Center for Outcomes Research and Evaluation (CORE), Research Institute, McGill University Health Center (RI-MUHC)<br>Department of Family Medicine, Faculty of Medicine and Health Sciences, McGill University<br>Chronic Viral Illness Service (CVIS), Royal Victoria Hospital/MUHC- Glen site<br>1001 Decarie Blvd, Room D02.4017<br>Montreal, Quebec, H4A 3J1, Canada<br>bertrand.lebouche@mcgill.ca
**Co-Investigators/sites:**	Sofiane Achiche, Ph.D.<br>Department of Mechanical Engineering, Polytechnique Montréal<br><br>Yuanchao Ma, M.Sc.A<br>CORE, RI-MUHC<br>Department of Mechanical Engineering, Polytechnique Montréal<br><br>Rachel Therrien, M.Sc. Pharmacist<br>University of Montréal Hospital Centre (CHUM)<br><br>Marie-Pascale Pomey, M.D., Ph.D.<br>Research Centre of the University of Montréal Hospital Centre (CRCHUM)<br>Centre of Excellence on Partnership with Patients and the Public (CEPPP)<br>Department of Health Policy, Management and Evaluation, School of Public Health, University of Montreal

Esli Osmanlliu, M.D., M.Sc.
Department of Pediatrics, Montreal Children's Hospital, MUHC
CORE RI-MUHC

Kim Engler, Ph.D.
CORE RI-MUHC

Serge Vicente, Ph.D.
Department of Family Medicine, Faculty of Medicine and Health Sciences, McGill University
Department of Mathematics and Statistics, University of Montreal

Benoît Lemire, M.Sc. Pharmacist
CVIS-MUHC

David Lessard, Ph.D.
CORE RI-MUHC

Jamil Asselah, M.D.
Department of Oncology, Royal Victoria Hospital/MUHC

Sponsor: Research Institute of the McGill University Health Centre

Funding: FRSQ Sida Maladies Infectieuses
CIHR SPOR mentorship Chair in innovative clinical trials in HIV care
Unité de soutien SSA Québec
Cedar Cancer Foundation

INTRODUCTION

We are inviting you to take part in this research study because you are identified as a key stakeholder for the MARVIN chatbots project.

Before you accept to take part in this study and sign this Informed consent form, please take the time to read, understand and carefully examine the following information.

This form may contain words that you do not understand. We encourage you to ask any questions you may have of the researcher in charge of the project or of other staff involved in the research project and to ask them to explain any words or information that is not clear.

BACKGROUND

This study is part of a master protocol that aims to adapt and evaluate the MARVIN chatbot to different healthcare contexts. Chatbots are software applications that interact with users by simulating a human conversation through text or voice via smartphones or computers. Often harnessing the power of artificial intelligence to enable natural language interpretation as well as aid decision-making, chatbots

can constitute a safe tool for patients to seek verified information. The MARVIN chatbot was initially developed for people living with HIV (PWH) by healthcare providers, engineers, researchers, and people with HIV at the McGill University Health Centre. It is deployed on Meta Messenger (Facebook) and is available any time in both English and French. MARVIN is trained to converse with PWH on the following self-management aspects: 1) guidance for antiretroviral therapy (ART) medication (in regard to time management, dosing, interactions, medication reminders, etc.), 2) ART management when traveling, and 3) common HIV-related knowledge (e.g., symptoms, modes of transmission and prevention, vaccination recommendation, etc.). A pilot study was done to evaluate the global usability of MARVIN among PWH, and users reported that MARVIN was tailored to their needs and was easy to use. The success of MARVIN with PWH deepens the interest of developing other chatbots for different health conditions and for patients or healthcare professionals.

PURPOSE OF THE RESEARCH STUDY

The acceptability and usability of the chatbot has been demonstrated for [arm's population]. The study objective is to conduct a research study for this new Chatbot to assess its implementation outcomes (i.e., uptake, fidelity, appropriateness, etc.) to see if it is worth adding to routine [condition/disease] care. This implementation study includes these following steps:

1. Participate in the screening and consent process
2. Complete the sociodemographic questionnaire received through the chatbot
3. Ask the chatbot questions through Meta messenger
4. Complete the implementation outcomes survey received through the chatbot every two weeks [time interval to be adapted based on condition/disease]
5. Answer an open-ended question to provide feedback on the overall experience with chatbot periodically

DESCRIPTION OF THE RESEARCH PROCEDURES

This research study will take place at the [Glen site of the McGill University Health Centre and/or Centre hospitalier de l'Université de Montréal].

5. Duration and number of visits

Your participation in this research study can last up to 12 months.

6. Study Procedures

The entire study participation lasts 12 months, and you will receive outcome measures every two months [time interval to be adapted based on condition/disease]. After consenting to participate, you can start using the chatbot. You will be able to contact a member of the team if you need help or if you have questions. See Table 1 for an overview of the study procedures.

<u>At entry</u>

You will complete a sociodemographic questionnaire online (time required: 10 minutes). It will ask you about your age, preferred language, gender/sex, sexual orientation, ethnicity, income, and education level. It will also ask about your use of mobile devices, health apps and Meta Messenger.

<u>During the study period</u>

You can use the chatbot as you wish. No additional specific actions are required to test the chatbot.

Every two months, you will be asked to fill in the implementation outcome questionnaire online. You will receive a relevant URL (i.e., RedCap, Google Form) directly through the chatbot. This will take about 15 minutes.

You will also be asked to answer the open-ended question through the chatbot about your experience in terms of overall satisfaction and suggestions for continuous improvement.

Table 1. Summary of study procedures for participants.

Study procedure	Facebook (Meta)	At entry	Month 1-2	Month 3-4	Month 5-6	Month 7-8	Month 9-10	Month 11-12
Participate in the screening and consent process	X	X						
Complete the sociodemographic questionnaire		X						
Ask the chatbot questions through Meta-messenger	X	X	X	X	X	X	X	X
Receive measurement tools every two months			X	X	X	X	X	X
Answer an open-ended question to provide feedback on the overall experience with chatbot			X	X	X	X	X	X

ELIGIBILITY CRITERIA
To be eligible to participate, you must meet the following criteria:

(1) being 18 years or older
(2) being fluent in English and/or French
(3) being able to understand the requirements of study participation and provide electronic informed consent during the duration of the study
(4) having access to a smartphone, tablet, or computer at home/at workplace
(5) having access to an internet connection at home or data plan on their device
(6) accept to use a Meta Messenger-based Chatbot
(7) accept to use or create a personal Meta account
(8) accept Meta’s privacy and data security policies

Exclusion criteria include:

(1) not meeting the inclusion criteria
(2) any reason, in the opinion of the investigator, which would make the candidate inappropriate for participation in an investigative study involving a chatbot (e.g., cognitive deficit)

PARTICIPANT'S RESPONSIBILITIES
If you wish to participate in this study, you will be asked to read, sign and date this consent form. Your answers to the eligibility screening questions and the electronically signed consent form will be saved in a separate encrypted database in the cloud server of the chatbot and synchronized to the registration log in RI-MUHC's internal server for record purposes.

If you are selected to participate in a focus group, please refrain from discussing content of focus group with non-participants.

BENEFITS ASSOCIATED WITH THE RESEARCH STUDY
There is no direct benefit to you for participating in this research. The information obtained from you could, however, help improve the care of other patients living with [condition/disease] in the future. More specifically, this study could contribute to the use of a new clinical tool for [arm's population].

RISKS ASSOCIATED WITH THE RESEARCH STUDY
We do not foresee any risks associated with this study and you are not at direct physical risk when participating in focus groups/interviews or conversing with chatbots via text messaging as you are not submitted to any pharmaceutical or invasive medical interventions. In addition, there were no known risks associated with participation in this type of study during the conduct of the pilot study (MUHC REB number: 2021-7191). However, there may be some potential risks.

With online recruitment, there may be risks of breach of confidentiality if you use your personal email address to communicate with a member of the study team. For this reason, researchers will communicate with you using institutional email addresses only. You are also advised to protect relevant personal electronic information.

For online use of the chatbot, you will use your personal Meta accounts to communicate with the chatbot. During use, you may share information about your participation in the study via Meta. There may be potential security breach of your MARVIN Meta account. To protect your personal information on Meta (Facebook), we advise you to 1) protect your login information including your email and your password; 2) log out of your Meta (Facebook) account after use when sharing your device with other people; 3) be vigilant against malicious software and suspicious links, even if they appear to come from a friend or a company you know. Additional security measures could be found at https://www.facebook.com/help/213481848684090 to keep your account secure. The MARVIN chatbots will also provide appropriate reminders (e.g., We recommend that you do not share information related to the study with others in unnecessary circumstances).

The time required to complete the questionnaire, participate in an interview or focus group may be inconvenient and stressful. It is also possible that you feel uncomfortable answering some questions. If you find the information or questions asked to be sensitive, private, or distressing, you do not have to answer those questions. The study team will be available to discuss your concerns and/or to refer you

to appropriate resources.

It may be possible that the MARVIN chatbots do not know what to answer when they cannot understand the messages you send during the study. In such cases, the chatbots will respond that it cannot understand and suggest you seek help from a healthcare professional. Furthermore, it is also possible that the chatbots answer with erroneous advice. For example, the chatbot informed a patient who had missed two hours of medication to stop taking the medication completely. To mitigate this potential risk, you will be explained of the limits of the chatbots during the consent process and prior its use. You will also be reminded to report to the study team all messages you receive that you believe to be incorrect and the impact they have had. These events will be monitored on an ongoing basis and discussed regularly by the governance committee of this study.

Due to the nature of focus groups, it is impossible to guarantee complete confidentiality as other members of the group will be aware of your identity. However, all participants are instructed to keep what is said in the focus group confidential, as mentioned in the “Participant’s Responsibilities” section.

VOLUNTARY PARTICIPATION AND THE RIGHT TO WITHDRAW

Your participation in this study is voluntary. Therefore, you may refuse to participate. You may also withdraw from the ongoing project at any time, without giving any reason, by informing a member of the study team. Your decision not to participate in the study, or to withdraw from it, will have no impact on the quality of care and services to which you are otherwise entitled, or no bearing on your job or on any work-related evaluations or reports. You will be informed in a timely manner if any information becomes available that may impact your willingness to continue participating in this study.

The researcher or the Research Ethics Board may put an end to your participation without your consent. This may happen if new findings or information indicate that participation is no longer in your interest, if you do not follow study instructions, or if there are administrative reasons to terminate the project.

If you withdraw or are withdrawn from the study, you may also request that the data already collected about you be removed from the study. If the data has been anonymized or was always anonymous (i.e., does not contain any information that can be used to identify you), the data will continue to be used in the analysis of the study.

Nonetheless, conversations with chatbots on Meta can be completely deleted. Inform a member of the research team if this is your wish. The research team will therefore delete all data collected from MARVIN’s account and ask you to do the same on your side. Once both parties have deleted the data, Meta Messenger will also not store it, as it will no longer provide the service.

If the Meta account you have chosen to use to participate in this study is disabled by Meta in accordance with their user policies, you will not be able to continue having access to the chatbot.

CONFIDENTIALITY

During your participation in this study, the doctor in charge of the study and the research team will collect in a study file the information about you needed to meet the scientific objectives of the study.

The information will be collected from the sociodemographic questionnaire, the implementation outcomes questionnaire, and the conversations you hold with the chatbot on Meta Messenger. Only information necessary for the research study will be collected.

In addition, participation in this study involves the use of Messenger application provided by Meta (Facebook). The use of Meta Messenger is mandatory to participate in the study. The use of the Meta Messenger involves the sharing of information about you. Part of the collection, processing, storage, and destruction of anonymized data is carried out by the company that provides the Meta Messenger application. This data will likely be saved in a cloud solution located outside of Canada (e.g., United States of America) and could be used by this company for a secondary use, such as business or marketing purposes. The research team, or [Name of the local institution] cannot guarantee the security (confidentiality, integrity, and availability) of this data. Assessing the risk to privacy involved in using the services of Meta is not part of the mandate of the Research Ethics Board of the [Name of the local institution]. You should therefore make sure that you understand the impact using this Meta Messenger will have on your privacy. If you would like more information, please further discuss it with the research team.

All study data collected during this research study (including personal information) will remain confidential to the extent provided by law. You will be identified by a code number only. The key to the code linking your name to your study file will be kept by the doctor in charge of this research study.

All audio-recordings will be transcribed (your words will be written down) in a de-identified fashion (i.e. your name will not appear in the transcripts). The audio-recordings will then be destroyed. It is possible that direct quotes of what you said will be presented in publications and/or conferences. However, precautions will be taken to ensure that it will not be possible to identify you.

The study investigator will use the study information collected about you for research purposes, only to reach the study goals as they are explained in this Information and Consent Document. Your study information will be kept by the investigator in charge of the study for 7 years from the date of publication.

The study information could be printed in medical journals or shared with other people at scientific meetings, but it will be impossible to identify you.

For auditing purposes, the research study files which could include documents that may identify you may be examined by a person mandated by:

- A representative of the Research Ethics Board who may also contact you to ask about your experience as research participant;
- [Name of the local institution]

All these individuals and organizations will have access to your personal data, but they adhere to a confidentiality policy.

For your safety and to be able to reach you quickly, your family name, first name, coordinates and the

date you started and ended the study will be kept for one year after the study ends in a separate list kept by the investigator in charge of the study or by the [Name of the local institution].

You have the right to look at your study file to check the information gathered about you and to correct it, if necessary, as long as the study investigator or [Name of the local institution] keeps this information. However, you may only have access to certain information once the study has ended.

FUNDING OF THE RESEARCH PROJECT
This study will be led by Dr. Bertrand Lebouché and conducted with internal funding. Be aware that study site doctors receive no direct financial compensation for enrolling you in this study.

CONFLICT OF INTERESTS
The researchers have no conflict of interest to declare.

COMPENSATION
For your time participating in this study, you be compensated at 60$ CAD after you have completed all of the following: the sociodemographic questionnaire, the 6 implementation outcomes questionnaires and associated open-minded questions for the chatbot. If you are invited to participate in the focus group, you will receive additional 30$ CAD after the focus group.

SHARING STUDY RESULTS
If you wish, you will receive a summary of research results by email.

SHOULD YOU SUFFER ANY HARM
Should you suffer harm of any kind following any procedure related to the research study, you will receive all the care and services required by your state of health.

By agreeing to participate in this research project, you are not waiving any of your rights nor discharging the researcher in charge of the study or the institution, of their civil and professional responsibilities.

CONTACT INFORMATION
If you have questions or if you have a problem you think may be related to your participation in this research study, or if you would like to withdraw, you may communicate with the study doctor [Doctor in charge of the local institution, with their contact information].

For any question concerning your rights as a research participant taking part in this study or if you have comments, or wish to file a complaint, you may communicate with the [Complaints Commissioner of the local institution, with their contact information].

OVERVIEW OF ETHICAL ASPECTS OF THE RESEARCH
The Research Ethics Board of the [Name of the local institution] has given ethics approval to this research study and is responsible for its ongoing ethics oversight at all participating institutions in the health and social services network in Quebec.

DECLARATION OF CONSENT

Research Study Title: [Title of the study's arm]
Implementation study.

I have reviewed the Informed Consent form. Both the research study and the Informed Consent form were explained to me. My questions were answered, and I was given sufficient time to make a decision. After reflection, I consent to participate in this research study in accordance with the conditions stated above, including the use of all personal data collected.

8) I consent to participate in the study:

Yes ☐ No ☐

9) I wish to receive a copy of the study results by email.

Yes ☐ No ☐ If yes, please provide contact information: __________________________

10) I authorize a member of the research study to communicate with me to see if I am interested in participating in other research studies.

Yes ☐ No ☐ If yes, please provide contact information: __________________________

____

Name of participant Signature Date

[Logo de l'institution locale]

FORMULAIRE D'INFORMATION ET DE CONSENTEMENT

Titre de l'étude: [Titre du bras de l'étude]
Étude d'implémentation

Numéro du protocole: [Numéro du protocole local du CER]

Chercheur responsable de l'étude:
Bertrand Lebouché MD, PhD,
Centre de recherche évaluative en santé (CRES)
Institut de recherche du Centre universitaire de santé McGill (IR-CUSM)
Département de médecine de famille, Faculté de médecine et des sciences de la santé, Université McGill
Service des maladies virales chroniques (SMVC), Hôpital Royal Victoria / Site Glen - CUSM
1001 boul. Decarie, salle D02.4017
Montréal, Qu.bec, H4A 3J1, Canada
bertrand.lebouche@mcgill.ca

Co-Investigateurs/sites:
Sofiane Achiche, Ph.D.
Département de génie mécanique, Polytechnique Montréal

Yuanchao Ma, M.Sc.A
CRES, IR-CUSM
Département de génie mécanique, Polytechnique Montréal

Rachel Therrien, M.Sc. Pharmacie
Centre hospitalier de l'Université de Montréal (CHUM)

Marie-Pascale Pomey, M.D., Ph.D.
Centre de recherche du Centre hospitalier de l'Université de Montréal (CRCHUM)
Centre d'excellence sur le partenariat avec les patients et le public (CEPPP)
Département de gestion, d'évaluation et de politique de santé, École de santé publique, Université de Montréal

Esli Osmanlliu, M.D., M.Sc.
Départment de pédiatrie, Hôpital de Montréal pour enfants, CUSM
CRES IR-CUSM

Kim Engler, Ph.D.
CRES IR-CUSM

Serge Vicente, Ph.D.
Département de médecine de famille, Faculté de médecine et des sciences de la santé, Université McGill
Département de mathématiques et statistique, Université de Montréal

Benoît Lemire, M.Sc. Pharmacist
Service des maladies virales chroniques (SMVC), CUSM

David Lessard, Ph.D.
CRES, IR-CUSM

Jamil Asselah, M.D.
Département d'oncologie, Hôpital Royal Victoria/CUSM

Sponsor: Institut de recherche du Centre universitaire de santé McGill

Financement: FRSQ Sida Maladies Infectieuses
IRSC Chaire de mentorat SPORT sur les essais cliniques novateurs dans le traitement du VIH
Unité de soutien SSA Québec
Fondation de cancer Cedar

INTRODUCTION

Nous vous invitons à participer à cette étude de recherche car vous êtes identifié comme une partie prenante clé du projet de chatbots MARVIN.

Avant d'accepter de participer à ce projet et de signer ce formulaire de d'information et de consentement, veuillez prendre le temps de lire, de comprendre et d'examiner attentivement les informations suivantes.

Ce formulaire peut contenir des mots que vous ne comprenez pas. Nous vous encourageons à poser toutes les questions que vous pourriez avoir au chercheur responsable du projet ou à d'autres membres du personnel impliqués dans le projet de recherche et à leur demander d'expliquer des mots ou des informations qui ne sont pas clairs.

CONTEXTE

Cette étude s'inscrit dans un protocole maître qui vise à adapter le chatbot MARVIN à différents contextes de soins de santé et à l'évaluer. Les chatbots sont des applications informatiques qui

interagissent avec les utilisateurs en simulant une conversation humaine par messagerie texte ou par la voix via des téléphones intelligents ou des ordinateurs. En exploitant l'intelligence artificielle pour permettre l'interprétation du langage naturel ainsi que pour faciliter la prise de décision, les chatbots peuvent constituer un outil sécuritaire pour que les patients puissent chercher des informations fiables. Le chatbot MARVIN a été initialement développé pour les personnes vivant avec le VIH (PVVIH) par des professionnels de santé, des ingénieurs, des chercheurs et des PVVIH au Centre universitaire de santé McGill. Il est déployé sur Meta Messenger (Facebook) et est disponible à tout moment en anglais et en français. MARVIN est formé pour converser avec les PVVIH sur les aspects d'auto-gestion suivants : 1) des conseils sur la médication d'une thérapie antirétrovirale (ARV) (en ce qui concerne la gestion du temps, le dosage, les interactions, les rappels de médicaments, etc.;) 2) la gestion des ARV lors de voyages; et 3) les connaissances communes liées au VIH (p. ex. symptômes, modes de transmission et de prévention, recommandation de vaccination, etc.). Une étude pilote a été réalisée pour évaluer l'utilisabilité globale de MARVIN parmi les PVVIH, et les utilisateurs ont indiqué que MARVIN était adapté à leurs besoins et était facile à utiliser. Le succès de MARVIN avec PVVIH approfondit l'intérêt de développer d'autres chatbots pour différentes conditions de santé ou pour les patients ou les professionnels de la santé.

OBJECTIFS DE L'ÉTUDE

L'acceptabilité et l'utilisabilité du chatbot ont maintenant été démontrées pour [population du bras de l'étude]. L'objectif de l'étude est de mener une étude de recherche pour ce nouveau chatbot afin d'évaluer les résultats de son implémentation (i.e., l'adoption, la fidélité, la pertinence, etc.) pour voir s'il vaut la peine de l'ajouter aux soin de routine [condition/maladie]. Cette étude d'implémentation comprend les étapes suivantes :

6. Participer à la procédure de sélection et de consentement
7. Remplir le questionnaire sociodémographique reçu par l'intermédiaire du chatbot
8. Poser des questions au chatbot par l'intermédiaire de Meta Messenger.
9. Répondre aux mesures d'impact de l'implémentation reçues par l'intermédiaire du chatbot toutes les deux semaines [intervalle de temps à adapter en fonction de l'état ou de la maladie].
10. Répondre à une question ouverte pour fournir un retour d'information sur l'expérience globale avec le chatbot périodiquement.

DESCRIPTION DES PROCÉDURES DE L'ÉTUDE

Cette étude de recherche se déroulera au [site Glen du Centre universitaire de santé McGill et/ou Centre hospitalier de l'Université de Montréal].

5. Durée

Votre participation à cette étude de recherche peut durer 12 mois.

6. Procédure de l'étude

La participation à l'étude dure 12 mois, vous allez recevoir des outils de mesure tous les deux mois [intervalle de temps à adapter en fonction de l'état ou de la maladie]. Après avoir accepté de participer, vous pouvez commencer à utiliser le chatbot. Vous pourrez contacter un membre de l'équipe si vous avez besoin d'aide ou si vous avez des questions. Voir le tableau 1 pour une vue d'ensemble des procédures de l'étude.

Au début de l’étude

Vous allez remplir un questionnaire sociodémographique en ligne (temps requis : 10 minutes). Il vous interrogera sur votre âge, votre langue préférée, votre genre/sexe, votre orientation sexuelle, votre origine ethnique, vos revenus et votre niveau d'éducation. Il vous interrogera également sur votre utilisation des appareils mobiles, des applications de santé et de Meta Messenger.

Pendant la période d'étude

Vous pouvez utiliser le chatbot comme vous le souhaitez. Aucune action spécifique supplémentaire n'est requise pour tester le chatbot.

Tous les deux mois, il vous sera demandé de remplir en ligne le mesure d’impact de l’implémentation. Vous recevrez une URL correspondante (i.e., RedCap, Google Form) directement par l'intermédiaire du chatbot. Cela prendra environ 15 minutes.

Il vous sera également demandé de répondre à une question ouverte par l'intermédiaire du chatbot sur votre expérience en termes de satisfaction globale et de suggestions d'amélioration continue.

Tableau 1. Résumé des procédures de l'étude pour les participants.

**Procédures de l’étude**	**Facebook (Meta)**	**Début**	**Mois 1-2**	**Mois 3-4**	**Mois 5-6**	**Mois 7-8**	**Mois 9-10**	**Mois 11-12**
Participer au processus de sélection et de consentement	X	X						
Remplir le questionnaire sociodémographique		X						
Poser des questions au chatbot via Meta-messenger	X	X	X	X	X	X	X	X
Recevoir des outils de mesure tous les deux mois			X	X	X	X	X	X
Répondre à une question ouverte pour donner son avis sur l'expérience globale avec le chatbot.			X	X	X	X	X	X

CRITÈRES D’ADMISSIBILITÉ

Pour être admissible à participer, vous devez satisfaire aux critères suivants :

(1) être âgé de 18 ans ou plus

(2) parler couramment le français et/ou l’anglais

(3) être en mesure de comprendre les exigences de la participation à l’étude et de fournir un

consentement éclairé électronique pendant la durée de l'étude
(4) avoir accès à un téléphone intelligent, une tablette ou un ordinateur à la maison / au travail
(5) avoir accès à une connexion Internet à la maison ou à un forfait de données sur leur appareil
(6) accepter d'utiliser un Chatbot basé sur Meta Messenger
(7) accepter d'utiliser ou de créer un compte personnel Meta
(8) accepter les politiques de confidentialité et de sécurité des données de Meta.

Les critères d'exclusion sont les suivants :
(1) ne pas satisfaire aux critères d'inclusion
(2) toute raison, de l'avis de l'intervieweur, qui rendrait le candidat inapproprié pour participer à une étude d'investigation impliquant un chatbot (par exemple, déficit cognitif)

RESPONSABILITÉS DU PARTICIPANT
Si vous souhaitez participer à cette étude, il vous sera demandé de lire, signer et dater ce formulaire de consentement. Vos réponses aux questions d'éligibilité et le formulaire de consentement signé électroniquement seront enregistrés dans une base de données cryptée distincte sur le serveur en nuage du chatbot et synchronisés avec le journal d'enregistrement sur le serveur interne du IR-CUSM à des fins d'archivage.

Si vous êtes sélectionné pour participer à un groupe de discussion, veuillez-vous abstenir de discuter du contenu du groupe de discussion avec les non-participants.

AVANTAGES LIÉS À L'ÉTUDE
Vous n'avez aucun avantage direct à participer à cette recherche. L'information obtenue de vous pourrait, cependant, aider à améliorer les soins d'autres patients vivant avec [condition/maladie] à l'avenir. Plus précisément, cette étude pourrait contribuer à l'utilisation d'un nouvel outil clinique pour [la population du bras de l'étude].

RISQUES LIÉS À L'ÉTUDE
Nous ne prévoyons pas de risques associés à cette étude et vous ne courez aucun risque physique direct lorsque vous participez à des groupes de discussion/entretiens ou lorsque vous conversez avec des chatbots par messagerie texte, car vous n'êtes soumis à aucune intervention pharmaceutique ou médicale invasive. En outre, aucun risque connu n'a été associé à la participation à ce type d'étude pendant la réalisation de l'étude pilote (numéro CUSM REB : 2021-7191). Cependant, il peut y avoir certains risques potentiels.

Avec le recrutement en ligne, il peut y avoir des risques de violation de la confidentialité si vous utilisez votre adresse électronique personnelle pour communiquer avec un membre de l'équipe d'étude. Pour cette raison, les chercheurs communiqueront avec vous en utilisant uniquement les adresses électroniques institutionnelles. Il vous est également conseillé de protéger les informations électroniques personnelles pertinentes.

Pour l'utilisation en ligne du chatbot, vous utiliserez vos comptes Meta personnels pour communiquer avec le chatbot. Pendant l'utilisation, vous pourrez partager des informations sur votre participation à l'étude via Meta. Il peut y avoir une violation potentielle de la sécurité de votre compte Meta MARVIN.

Pour protéger vos informations personnelles sur Meta (Facebook), nous vous conseillons de 1) protéger vos informations de connexion, y compris votre adresse électronique et votre mot de passe ; 2) vous déconnecter de votre compte Meta (Facebook) après utilisation lorsque vous partagez votre appareil avec d'autres personnes ; 3) être vigilant à l'égard des logiciels malveillants et des liens suspects, même s'ils semblent provenir d'un ami ou d'une entreprise que vous connaissez. D'autres mesures de sécurité peuvent être trouvées sur le site https://www.facebook.com/help/213481848684090 pour sécuriser votre compte. Les chatbots MARVIN fourniront également des rappels appropriés (par exemple, Nous vous recommandons de ne pas partager les informations relatives à l'étude avec d'autres personnes dans des circonstances inutiles).

Le temps nécessaire pour remplir le questionnaire, participer à un entretien ou à un groupe de discussion peut être gênant et stressant. Il est également possible que vous vous sentiez mal à l'aise pour répondre à certaines questions. Si vous trouvez que les informations ou les questions posées sont sensibles, privées ou pénibles, vous n'êtes pas obligé de répondre à ces questions. L'équipe de l'étude sera disponible pour discuter de vos préoccupations et/ou pour vous orienter vers les ressources appropriées.

Il est possible que les chatbots MARVIN ne sachent pas quoi répondre lorsqu'ils ne comprennent pas les messages que vous envoyez pendant l'étude. Dans ce cas, les chatbots répondront qu'ils ne peuvent pas comprendre et vous suggéreront de demander l'aide d'un professionnel de la santé. Par ailleurs, il est également possible que les chatbots répondent par des conseils erronés. Par exemple, le chatbot a conseillé à un patient qui avait oublié de prendre son médicament pendant deux heures d'arrêter complètement de le prendre. Pour atténuer ce risque potentiel, les limites des chatbots vous seront expliquées au cours du processus de consentement et avant leur utilisation. Il vous sera également rappelé de signaler à l'équipe de l'étude tous les messages que vous recevez et qui vous semblent incorrects, ainsi que l'impact qu'ils ont eu. Ces événements seront classés comme des événements indésirables pour cette étude. Ces événements feront l'objet d'un suivi permanent et seront discutés régulièrement par le comité de gouvernance de cette étude.

En raison de la nature des groupes de discussion, il est impossible de garantir une confidentialité totale, car les autres membres du groupe connaîtront votre identité. Toutefois, tous les participants sont tenus de respecter la confidentialité de ce qui est dit dans le groupe de discussion, comme indiqué dans la section "Responsabilités des participants".

PARTICIPATION VOLONTAIRE ET DROIT DE RETRAIT

Votre participation à cette étude est volontaire. Vous pouvez donc refuser d'y participer. Vous pouvez également vous retirer du projet en cours à tout moment, sans donner de raison, en informant un membre de l'équipe de l'étude. Votre décision de ne pas participer à l'étude ou de vous en retirer n'aura aucune incidence sur la qualité des soins et des services auxquels vous avez droit par ailleurs, ni sur votre emploi ou sur les évaluations ou rapports liés à votre travail. Vous serez informé(e) en temps utile de toute information susceptible d'avoir une incidence sur votre volonté de continuer à participer à cette étude.

Le chercheur ou le comité d'éthique de la recherche peut mettre un terme à votre participation sans votre consentement. Cela peut se produire si de nouvelles découvertes ou informations indiquent que

la participation n'est plus dans votre intérêt, si vous ne suivez pas les instructions de l'étude ou s'il existe des raisons administratives de mettre fin au projet.

Si vous vous retirez ou êtes retiré de l'étude, vous pouvez également demander que les données déjà collectées à votre sujet soient retirées de l'étude. Si les données ont été anonymisées ou ont toujours été anonymes (c'est-à-dire qu'elles ne contiennent aucune information permettant de vous identifier), elles continueront à être utilisées dans l'analyse de l'étude.

Néanmoins, les conversations avec les chatbots sur Meta peuvent être complètement supprimées. Informez un membre de l'équipe de recherche si tel est votre souhait. L'équipe de recherche supprimera alors toutes les données recueillies sur le compte de MARVIN et vous demandera de faire de même de votre côté. Une fois que les deux parties auront supprimé les données, Meta Messenger ne les conservera pas non plus, car il ne fournira plus le service.

Si le compte Meta que vous avez choisi d'utiliser pour participer à cette étude est désactivé par Meta conformément à leurs politiques d'utilisation, vous ne pourrez pas continuer à avoir accès au chatbot.

CONFIDENTIALITÉ

Pendant votre participation à cette étude, le médecin responsable de l'étude et l'équipe de recherche recueilleront dans un dossier d'étude les informations vous concernant nécessaires pour atteindre les objectifs scientifiques de l'étude.

Les informations seront collectées à partir du questionnaire sociodémographique, du questionnaire de l'implémentation, et des conversations que vous tenez avec le chatbot sur Meta Messenger. Seules les informations nécessaires à l'étude de recherche seront collectées.

En outre, la participation à cette étude implique l'utilisation de l'application Messenger fournie par Meta (Facebook). L'utilisation de Meta Messenger est obligatoire pour participer à l'étude. L'utilisation de Meta Messenger implique le partage d'informations vous concernant. Une partie de la collecte, du traitement, du stockage et de la destruction des données anonymes est effectuée par la société qui fournit l'application Meta Messenger. Ces données seront probablement sauvegardées dans une solution en nuage située à l'extérieur du Canada (par exemple, aux États-Unis d'Amérique) et pourraient être utilisées par cette entreprise pour un usage secondaire, par exemple à des fins commerciales ou de marketing. L'équipe de recherche, ou [Nom de l'institution locale] ne peuvent garantir la sécurité (confidentialité, intégrité et disponibilité) de ces données. L'évaluation des risques pour la vie privée liés à l'utilisation des services de Meta ne fait pas partie du mandat du Comité d'éthique de la recherche du [Nom de l'institution locale]. Vous devez donc vous assurer que vous comprenez l'impact de l'utilisation du Meta Messenger sur votre vie privée. Si vous souhaitez obtenir de plus amples informations, veuillez en discuter avec l'équipe de recherche.

Toutes les données recueillies au cours de cette étude de recherche (y compris les renseignements personnels et les échantillons) resteront confidentielles dans les limites prévues par la loi. Vous ne serez identifié que par un numéro de code. La clé du code reliant votre nom à votre dossier d'étude sera conservée par le médecin responsable de cette étude de recherche.

Tous les enregistrements audios seront transcrits (vos paroles seront écrites) de manière dépersonnalisée (c'est-à-dire que votre nom n'apparaîtra pas dans les transcriptions). Les enregistrements audios seront ensuite détruits. Il est possible que des citations directes de vos propos soient présentées dans des publications et/ou des conférences. Toutefois, des précautions seront prises pour s'assurer qu'il ne sera pas possible de vous identifier.

L'investigateur de l'étude utilisera les informations recueillies à votre sujet à des fins de recherche, uniquement pour atteindre les objectifs de l'étude tels qu'ils sont expliqués dans le présent document d'information et de consentement. Vos informations d'étude seront conservées par le chercheur responsable de l'étude pendant 7 ans à compter de la date de publication.

Les informations de l'étude pourraient être imprimées dans des revues médicales ou partagées avec d'autres personnes lors de réunions scientifiques, mais il sera impossible de vous identifier.

Pour s'assurer que l'étude est faite correctement; votre dossier d'étude de recherche ainsi que votre dossier médical pourraient être vérifiés par une personne autorisée par:

- Un représentant du comité d'éthique de la recherche qui peut également communiquer avec vous pour vous renseigner sur votre expérience en tant que participant à la recherche;
- [Nom de l'institution locale]

Pour votre sécurité et pour être en mesure de vous joindre rapidement, votre nom de famille, votre prénom, vos coordonnées et la date à laquelle vous avez commencé et terminé l'étude seront conservés pendant un an après la fin de l'étude dans une liste distincte tenue par le chercheur responsable de l'étude ou par [Nom de l'institution locale].

Vous avez le droit de consulter votre dossier d'étude pour vérifier les renseignements recueillis à votre sujet et pour les corriger, au besoin, à condition que le chercheur de l'étude ou [Nom de l'institution locale] conserve ces renseignements. Toutefois, il se peut que vous n'ayez accès à certains renseignements qu'une fois l'étude terminée.

FINANCEMENT DE L'ÉTUDE

Cette étude sera dirigée par le Dr Bertrand Lebouché et réalisée avec un financement interne. Sachez que les médecins du site d'étude ne reçoivent aucune compensation financière directe pour vous inscrire à cette étude.

CONFLITS D'INTÉRÊTS

Les chercheurs n'ont aucun conflit d'intérêt à déclarer.

COMPENSATION

Pour votre temps de participation à cette étude, vous recevrez une compensation de 60$ CAD après avoir rempli tous les éléments suivants : le questionnaire sociodémographique, les 6 mesures d'impact de l'implémentation et les questions d'ouverture d'esprit associées au chatbot. Si vous êtes invité à participer au groupe de discussion, vous recevrez 30$ CAD supplémentaires après le groupe de discussion.

PARTAGE DES RÉSULTATS DE L'ÉTUDE
Si vous le souhaitez, vous recevrez un résumé des résultats de la recherche par courrier électronique.

SI VOUS SUBISSEZ UNE BLESSURE
Si vous subissez une blessure de quelque nature à la suite d'une procédure liée à l'étude de recherche, vous recevrez tous les soins et services requis par votre état de santé sans aucun coût pour vous.

En acceptant de participer à ce projet de recherche, vous ne renoncez à aucun de vos droits et ne déchargez pas le chercheur chargé de l'étude ou l'institution de leurs responsabilités civiles et professionnelles.

COORDONNÉES DE LA PERSONNE-RESSOURCE
Si vous avez des questions ou si vous avez un problème qui, selon vous, pourrait être lié à votre participation à cette étude de recherche, ou si vous souhaitez vous retirer, vous pouvez communiquer avec le médecin de l'étude [Médecin responsable de l'institution locale, avec ses coordonnées].

Pour toute question concernant vos droits en tant que participant à la recherche participant à cette étude ou si vous avez des commentaires ou souhaitez déposer une plainte, vous pouvez communiquer avec le [Commissaire aux plaintes de l'institution locale, avec ses coordonnées].

CONTRÔLE DES ASPECTS ÉTHIQUE DE L'ÉTUDE
Le Comité d'éthique de la recherche du [Nom de l'institution locale] a donné son approbation éthique à cette étude de recherche et est responsable de sa surveillance éthique continue dans tous les établissements participants du réseau de la santé et des services sociaux du Québec.

DÉCLARATION DE CONSENTEMENT

Titre de l'étude: [Titre du bras de l'étude]
Étude d'utilisabilité

J'ai examiné le formulaire de consentement éclairé. L'étude de recherche et le formulaire de consentement éclairé m'ont été expliqués. On a répondu à mes questions et on m'a laissé suffisamment de temps pour prendre une décision. Après réflexion, je consens à participer à cette étude de recherche conformément aux conditions énoncées ci-dessus, y compris l'utilisation de toutes les données personnelles recueillies.

8) Je consens à participer à l'étude :

Oui ☐ Non ☐

9) Je souhaite recevoir une copie des résultats de l'étude par courriel.

Oui ☐ Non ☐ Si oui, veuillez fournir votre adresse courriel : ________________________

10) J'autorise l'équipe d'étude de recherche de cette étude à communiquer directement avec moi pour me demander si je suis intéressé à participer à d'autres recherches:

Oui ☐ Non ☐ Si oui, veuillez fournir votre adresse courriel : ________________________

____

Nom du participant Signature Date
