# Supplementary material for: Adapting and Evaluating an AI-Based Chatbot Through Patient and Stakeholder Engagement to Provide Information for Different Health Conditions: Master Protocol for an Adaptive Platform Trial (the MARVIN Chatbots Study)
Source: JMIR Res Protoc. 2024 Feb 13;13:e54668. doi: 10.2196/54668 (PMC10900097; doi:10.2196/54668)
Supplement: Multimedia Appendix 7 [file resprot_v13i1e54668_app7.pdf]

Table 1. Summary of study procedures for participants.

**Objective 1 (development)**									
Study procedure	Interactions with Facebook	At entry	Weeks 1-4	Week 6	Week 8	Week 10	Week 12	Week 14	Week 16
Screening and consent process		✓							
User needs assessment—2-hour focus groups			✓						
Knowledge database creation—2-hour co-construction workshops				✓		✓		✓	
Test, validation, and continuous improvement—2-hour co-construction workshops	✓				✓		✓		✓

**Objective 2 (usability)**						
Study procedure	Facebook	At entry	Week 1	Week 2	Week 3	Week 4
Screening and consent process		✓				
Ask the chatbot questions through Messenger	✓	✓	✓	✓	✓	
Sociodemographic questionnaire		✓				

Usability survey					✓	
2-hour focus groups						✓

Objective 3 (implementation)

Study procedure	Facebook	At entry	Months 1-2	Months 3-4	Months 5-6	Months 7-8	Months 9-10	Months 11-12
Screening and consent process	✓	✓						
Sociodemographic questionnaire		✓						
Ask the chatbot questions through Messenger	✓	✓	✓	✓	✓	✓	✓	✓
Receive measurement tools every 2 months			✓	✓	✓	✓	✓	✓
Answer 3 open-ended questions to provide feedback on the overall experience with the chatbot			✓	✓	✓	✓	✓	✓

Objective 4 (partnership evaluation)

Study procedure	Objective 1	Objective 2	Objective 3
2-hour focus groups	✓	✓	✓
