# Supplementary material for: Adapting and Evaluating an AI-Based Chatbot Through Patient and Stakeholder Engagement to Provide Information for Different Health Conditions: Master Protocol for an Adaptive Platform Trial (the MARVIN Chatbots Study)
Source: JMIR Res Protoc. 2024 Feb 13;13:e54668. doi: 10.2196/54668 (PMC10900097; doi:10.2196/54668)
Supplement: Multimedia Appendix 8 [file resprot_v13i1e54668_app8.pdf]

Appendix 7.1 - Adapted Mobile App Rating Scale (A-MARS)

Adapted Mobile App Rating Scale (A-MARS)
APP / ETOOL NAME: The MARVIN Chatbots
SECTION A
Engagement – fun, interesting, customisable, interactive, has prompts (e.g. sends alerts, messages, reminders, feedback, enables sharing)

1. **Engagement**: Is the app/e-tool engaging for the user? Do you feel engaged enough to complete the e-tool program or use the app on multiple occasions? Does it have components that make you want to use it more than similar apps/e-tools?
   1 Dull, not engaging, not encouraged to start using app/e-tool
   2 Mostly boring, would start program but never finish OR would download app and only use once or twice
   3 OK, engaging enough to us app for a brief time (<5 min), would finish up to half an e-tool program
   4 Moderately engaging, would use app/e-tool for some time; most likely complete the full program
   5 Highly engaging, would stimulate repeat use of app/e-tool or would use for 5–10 min, OR would finish the full program, complete additional programs and/or return to use program again and engaged in tools/strategies given/learnt

2. **Interest:** Is the app/e-tool interesting to use? Does it present information in an interesting way compared to other apps/e-tools or offline/ traditional tools?
   1 Not interesting at all,
   2 Mostly uninteresting
   3 OK, neither interesting nor uninteresting; appears the same as offline/traditional tools/similar apps
   4 Moderately interesting; would engage user for some time, somewhat more interesting than offline/traditional tools/similar apps
   5 Very interesting, would engage user in repeat use, more engaging than traditional/offline tools/similar apps

3. **Customisation:** Does the app/e-tool need to be customised to make it more user friendly for you to use? Can you change settings such as sound, content, notifications, email/SMS reminders, display more to your liking?
   1 App/e-tool is not user friendly; has no customisation options or requires setting to be input every time
   2 App/e-tool allows little customisation; App/e-tool could be improved with more customisation options
   3 Basic customisation to function adequately and/or can use app/e-tool without customization
   4 Allows numerous options for customisation and/or easy to use app/e-tool; customisation somewhat unnecessary
   5 Does not need any customisation for me to use the app/e-tool effectively; Allows complete tailoring the user’s characteristics/preferences, remembers all settings

4. **Interactivity/Interoperability:** Does it allow user input, provide feedback, contain prompts (reminders, sharing options, notifications, etc.)? Does the app/e-tool adapt based on user input? Does it allow exchange of data with other apps, e-tools or wearable devices (if applicable)?
   1 No interactive and features and/or no response to user input; does not adapt based off user information; has no function for exchange of data with other apps, e-tools or wearables (if applicable)
   2 Some, but not enough interactive and/or interoperability features which limits app/e-tool functions; some adaptability
   3 Basic interactive features to function adequately; has some capacity for exchanging data with other apps, e-tools or wearables (if applicable)
   4 Offers a variety of interactive features, feedback and user input options, app/e-tool adapts somewhat to user input; with some effort can exchange data with multiple different apps, e-tools and wearables (if applicable)
   5 Very high level of responsiveness through interactive features, feedback and user input options; app/e-tool adapts as user inputs; allows easy exchange of data with apps, e-tools or wearable devices (if applicable)

5. **Target group:** Is the content (visuals, language, design) appropriate for the target audience?
   1 Completely inappropriate, unclear or confusing
   2 Mostly inappropriate, unclear or confusing

3 Acceptable but not specifically designed for the target audience. May be inappropriate/ unclear/confusing at times
4 Designed for the target audience, with minor issues 5 Designed specifically for the target audience, no issues found

A. Engagement mean score = ___________

SECTION B
Functionality –app/e-tool functioning, easy to learn, navigation, flow logic, and intuitive design of app/e-tool

6. Performance: How accurately/fast does the app/e-tool run (functions) and do all components with the app/e-tool (buttons/menus) work? Are there any error messages, glitches, crashes?
1 App/e-tool is broken; no/insufficient/inaccurate response (e.g. crashes/bugs/broken features, etc.)
2 Some functions work, but lagging or contains major technical problems
3 App/e-tool works overall. Some technical problems need fixing, or is slow at times
4 Mostly functional with minor/negligible problems
5 Perfect/timely response; no technical bugs found, or contains a 'loading time left' indicator (if relevant)

7. Ease of use: How easy is it to learn how to use the app/e-tool; how clear are the menu labels, icons and instructions? Is the sign-up process quick and/or simple? Are there relevant help buttons/FAQ's?
1 No/limited instructions; menu labels, icons are confusing; complicated; sign up process is complicated with no help buttons/FAQ's
2 Takes a lot of time or effort, sign up process is somewhat complicated and/or asks for too much information and/or offers little help
3 Takes some time or effort
4 Easy to learn (or has clear instructions); sign up process relatively simple; some help/FAQ's
5 Able to use app/e-tool immediately; intuitive; simple (no instructions needed); relevant support is obvious and helpful

8. Navigation: Does moving between screens make sense; Is it easy to move from one section of the app/e-tool to another? Does the app/e-tool provide all necessary links between screens?
1 No logical connection between screens at all/navigation is difficult
2 Understandable after a lot of time/effort
3 Understandable after some time/effort
4 Easy to understand/navigate
5 Perfectly logical, easy, clear and intuitive screen flow throughout, and/or has shortcuts

9. Design: Are there intuitive popup boxes, videos, animations, audio clips, flash images etc within the e-tool or are there consistent taps/swipes, pinches/scrolls within the app/e-tool? Are these relevant/accurate/make sense and in theme with the rest of the app/e-tool?
1 Completely confusing/inconsistent, information lacks relevance or is inaccurate/unnecessary
2 Often confusing/inconsistent, information of little relevance or contains some unnecessary/incorrect
3 Okay, some confusing and/or unnecessary information or some inconsistencies
4 Mostly intuitive, with negligible problems with majority of information is accurate/necessary
5 Perfectly consistent and intuitive, information is accurate/necessary

B. Functionality mean score = ___________

SECTION C
Aesthetics – graphic design, overall visual appeal, colour scheme, and stylistic consistency

10. Layout: Is arrangement and size of buttons, icons, menus and content on the screen appropriate?
1 Very bad design, cluttered, some options impossible to select, locate, see or read
2 Bad design, random, unclear, some options difficult to select/locate/see/read
3 Satisfactory, few problems with selecting/locating/seeing/reading items
4 Mostly clear, able to select/locate/see/read items
5 Professional, simple, clear, orderly, logically organised

11. Graphics: How high is the quality/resolution of graphics used for buttons, icons, menus and content?

1 Graphics appear amateur, very poor visual design - disproportionate, stylistically inconsistent
2 Low quality/low resolution graphics; low quality visual design – disproportionate
3 Moderate quality graphics and visual design (generally consistent in style)
4 High quality/resolution graphics and visual design – mostly proportionate, consistent in style
5 Very high quality/resolution graphics and visual design - proportionate, consistent in style throughout

12. **Visual appeal:** How good does the app/e-tool look?
1 Ugly, unpleasant to look at, poorly designed, clashing, mismatched colours
2 Bad – poorly designed, bad use of colour, visually boring
3 OK – average, neither pleasant, nor unpleasant
4 Pleasant – seamless graphics – consistent and professionally designed
5 Beautiful – very attractive, memorable, stands out; use of colour enhances app/e-tool features/menus

C. Aesthetics mean score = ___________

SECTION D

Information – Contains high quality information (e.g. text, feedback, measures, references) from a credible source

13. **Goals:** Does app/e-tool have specific, measurable and achievable goals (are these goals specified/obvious within the app/e-tool)?
N/A Description does not list goals, or app/e-tool goals are irrelevant to research goal (e.g. using a game for educational purposes)
1 App/e-tool has no chance of achieving its stated goals
2 Description lists some goals, but app/e-tool has very little chance of achieving them
3 OK. App/e-tool has clear goals, which may be achievable.
4 App/e-tool has clearly specified goals, which are measurable and achievable
5 App/e-tool has specific and measurable goals, which are highly likely to be achieved

14. **Quality of information:** Is the content within the app/e-tool correct (including description in app store – if an app)? Is app/e-tool up to date with current research, well written, and relevant to the goal/topic of the app/e-tool?
N/A There is no information within the app/e-tool
1 Irrelevant/inappropriate/incoherent/incorrect
2 Poor. Barely relevant/appropriate/coherent/may be incorrect
3 Moderately relevant/appropriate/coherent/and appears correct
4 Relevant/appropriate/coherent/correct
5 Highly relevant, appropriate, coherent, and correct

15. **Quantity of information:** Is the information within the app/e-tool comprehensive and/or relevant but concise?
N/A There is no information within the app/e-tool
1 Minimal or overwhelming
2 Insufficient or possibly overwhelming
3 OK but not comprehensive or concise
4 Offers a broad range of information, has some gaps or unnecessary detail; or has no links to more information and resources
5 Comprehensive and concise; contains links to more information and resources

16. **Visual information:** Is visual explanation of concepts – through charts/graphs/images/videos, etc. – clear, logical, correct?
N/A There is no visual information within the app/e-tool (e.g. it only contains audio, or text)
1 Completely unclear/confusing/wrong or necessary but missing
2 Mostly unclear/confusing/wrong
3 OK but often unclear/confusing/wrong
4 Mostly clear/logical/correct with negligible issues
5 Perfectly clear/logical/correct

17. Credibility of source: does the information within the app/e-tool seem to come from a credible source?
1 Source identified but legitimacy/trustworthiness of source is questionable (e.g. commercial business with vested interest)
2 Appears to come from a legitimate source, but it cannot be verified (e.g. has no webpage)
3 Developed by small NGO/institution (hospital/centre, etc.) /specialised commercial business, funding body
4 Developed by government, university or as above but larger in scale
5 Developed using nationally competitive government or research funding (e.g. Australian Research Council, NHMRC)

18. Evidence base: Has the app/e-tool been trialled/tested; must be verified by evidence (in published scientific literature)?
N/A It has not been trialled/tested
1 The evidence suggests the app/e-tool does not work
2 App/e-tool has been trialled (e.g., acceptability, usability, satisfaction ratings) and has partially positive outcomes in studies that are not randomised controlled trials (RCTs), or there is little or no contradictory evidence.
3 App/e-tool has been trialled (e.g., acceptability, usability, satisfaction ratings) and has positive outcomes in studies that are not RCTs, and there is no contradictory evidence.
4 App/e-tool has been trialled and outcome tested in 1-2 RCTs indicating positive results
5 App/e-tool has been trialled and outcome tested in > 3 high quality RCTs indicating positive results

D. Information mean score = ___________*
*****Exclude questions rated as "N/A" from the mean score calculation

---

SECTION E
App/e-tool subjective quality rating

19. Would you recommend this app/e-tool to people who might benefit from it?
1 Not at all - I would not recommend this app/e-tool to anyone
2 There are very few people I would recommend this app/e-tool to
3 Maybe - There are several people whom I would recommend it to
4 There are many people I would recommend this app/e-tool to
5 Definitely - I would recommend this app/e-tool to everyone

20. How many times do you think you would use this app/e-tool in the next 12 months if it was relevant to you?
1 None
2 1-2
3 3-10
4 10-50
5 >50

21. Would you pay for this app/e-tool?
1 No
3 Maybe
5 Yes

22. What is your overall star rating of the app/e-tool?
1 ★ One of the worst apps/e-tools I've used
2 ★★
3 ★★★Average
4 ★★★★
5 ★★★★★ One of the best apps/e-tools I've used

E. Subjective mean score = ___________

SECTION F
Supplement Health Related App/e-tools: Questions to consider when using a health-related app/e-tool

23. **Additional resources available.** Does the app/e-tool provide up to date relevant offline/online resources to support the information presented?
    1 No – provides no further resources
    2 Provides few online OR offline resources
    3 Somewhat – provides some offline and/or online resources, may be outdated
    4 Provides adequate online and/or offline resources
    5 Yes – provides abundant and up to date offline and online resources

24. **Strategies:** Does the app/e-tool recommend strategies that are non-tech based and linked to the problems you have reported?
    1 No – none
    2
    3 Somewhat – some information; may be too much, or too little resources
    4
    5 Yes – adequate/plentiful but not overbearing

25. **Solutions:** Does it offer multiple solutions for one issue?
    1 No – offers one solution to address one issue/health symptom
    2
    3 Some – offers some solutions for the one issue/health symptom; offers solutions but they indirectly address the issue
    4
    5 Yes – offers various related solutions to directly address the issue

26. **Multiple health issues/symptoms:** Does it address more than one symptom or health issue?
    1 No – addresses one symptom/health issue only
    2
    3 Some – addresses some symptoms/health issues; or considers many but only partly address them
    4
    5 Yes – considers multiple symptoms/health issues and related ones, and sufficiently addresses them

27. **Real time tracking:** Can you use the app/e-tool in real time, as you're experiencing a health issue?
    1 No – the app/e-tool is mainly useful for prevention or recovery
    2
    3 The app/e-tool is useful for prevention, management and/or recovery of the health issue(s)
    4
    5 Yes – the app/e-tool is useful for prevention, management and recovery of the health issue(s)

28. **Access to help:** Easy/obvious to access health related help when needed?
    1 No – Difficult to navigate or find related health information when needed
    2 Can find needed information after a lot of time/effort
    3 Can find needed information after some time/effort
    4 Easy to understand/navigate needed information
    5 Perfectly logical, easy, clear and intuitive screen flow throughout, and/or has shortcuts to needed health information. Offline options are available.

F. Health-related information mean score = ___________

Scoring App/e-tool quality scores:
SECTION
A: Engagement Mean Score = _________________________
B: Functionality Mean Score = __________________________
C: Aesthetics Mean Score = __________________________
D: Information Mean Score = ___________________________

Quality mean Score = ___________________________
E. Subjective quality Score = ________________________
F. Health-related quality Score = ________________________

Appendix 7.2 - Sociodemographic questionnaire for the usability study and chatbot outcomes

1. Preferred language:
    - ○ English
    - ○ French
    - ○ Other: __________

2. How old are you?
    - ○ _________

3. What is your gender identity? Select all that applies.
    - ▢ Man
    - ▢ Woman
    - ▢ Non-binary
    - ▢ I identify as __________

4. With respect to your sexual orientation, how do you currently identify? Select all that apply.
    - ▢ Heterosexual/Straight
    - ▢ Lesbian
    - ▢ Gay
    - ▢ Queer
    - ▢ Bisexual
    - ▢ Two-spirited
    - ▢ Questioning
    - ▢ I identify as _________

5. What single or multiple ethnic group(s) or family background(s) do you identify with? Select all that apply.
    - ▢ Aboriginal or Indigenous
    - ▢ English Canadian
    - ▢ French Canadian
    - ▢ French
    - ▢ British
    - ▢ Other Eastern/Western European
    - ▢ East Asian
    - ▢ South Asian
    - ▢ West Asian
    - ▢ Arab or North African
    - ▢ Latin American
    - ▢ African
    - ▢ Black
    - ▢ Caribbean
    - ▢ Pacific
    - ▢ Mixed race/ethnicity
    - ▢ I identify as _________

6. What is the highest level of education you have completed?
    - ○ No formal education
    - ○ Primary
    - ○ Secondary (High school)
    - ○ Professional degree/College
    - ○ CEGEP/Technical degree
    - ○ University
    - ○ Other:

7. Annual income ($CAD)

- Less than $10,000
- $10,000 - $19,999
- $20,000 - $39,999
- $40,000 - $59,999
- $60,000 - $79,999
- $80,000 - $99,999
- Greater than $100,000
- Other:

8. How long ago were you diagnosed with [condition/disease]? / How long ago have you started working with [condition/disease]?
    - ____ years and/or ____ months

9. Which of the following (mobile) device do you use most frequently:
    - Android smartphone
    - Apple iPhone
    - Tablet
    - Computer/laptop
    - Other: ______

10. How frequently do you use your (mobile) device?
    - Several times a day
    - Once a day
    - Several times per week
    - Several times per month

11. What is your level of agreement with the following statement: I am confident that I can effectively use a (mobile) device or applications related to health (e.g. patient portal, lifestyle and diet information, activity tracker).
    - Strongly disagree
    - Disagree
    - Neutral
    - Agree
    - Strongly Agree

12. To what extent do you use applications related to health on your (mobile) device(s)?
    - Never
    - Very little
    - Sometimes
    - Frequently
    - Very frequently

13. How many years have you been using Meta (Facebook) Messenger regularly?
    - Less than 2 years
    - 2-4 years
    - 4-6 years
    - 6-8 years
    - 8-10 years
    - More than 10 years

Appendix 7.3 - Usability survey for the usability study

UMUX-Lite (global usability)
Likert scale 1-7: 1 = strongly disagree, 7 = strongly agree

1. The chatbot's capabilities meet my requirements.
2. The chatbot is easy to use.

Perceived usefulness (effectiveness) - Modified
Likert scale 1-7: 1 = strongly disagree, 7 = strongly agree

3. Using the chatbot can improve my patient care and management. / Using the chatbot can improve my quality of work.
4. Using the chatbot can enhance my effectiveness in [condition/disease] self-care and management. / Using the chatbot can enhance my effectiveness in my work.
5. Using the chatbot can make my [condition/disease] self-care and management easier. / Using the chatbot can make my work easier.
6. I would find the chatbot useful for my [condition/disease] self-care and management. / I would find the chatbot useful for my work.

Perceived ease of use (efficiency)
Likert scale 1-7: 1 = very difficult, 7 = very easy

7. Overall, how difficult or easy did you find using the chatbot?

Attitude towards using (satisfaction)
Scale 0-10: 0 = not likely, 10 = very likely

8. How likely are you to recommend the chatbot to a friend / How likely are you to recommend the chatbot to a colleague?

Behavioural intention (intention to use)
Likert scale 1-7: 1 = strongly disagree, 7 = strongly agree

9. Assuming I have access to the chatbot, I intend to use it.
10. Given that I have access to the chatbot, I predict that I would use it.

AES -Modified (global usability)
Likert scale 1-5

11. How easy was the chatbot for you to use? 1 = very difficult, 5 = very easy
12. How understandable were the chatbot's answers to your questions? 1 = difficult to understand, 5 = easy to understand
13. How much did you enjoy using the chatbot? 1 = not at all, 5 = very much
14. How helpful was the chatbot in answering your questions? 1 = very unhelpful, 5 = very helpful
15. Was the amount of time it took to use the chatbot acceptable? 1 = very unacceptable, 5 = very acceptable
16. How would you rate your overall satisfaction with the chatbot? 1 = very dissatisfied, 5 = very satisfied

Appendix 7.4 - Semi-structured interview guide for the usability study

1. What led you to enroll in this study? (general interest/motivation)

2. How easy or hard was it for you to learn to use the chatbot? (perceived ease of use)
    a. • Prompt: What made the chatbot easy to use?

    b. • Prompt: What made the chatbot hard to use?

3. How helpful did you find your conversations with the chatbot? (perceived usefulness)
    a. • Prompt: What was most helpful about the chatbot?

    b. • Prompt: What was least helpful?

4. How satisfied are you overall with the chatbot? (attitude towards use)

5. How likely do you think the chatbot could provide information that could disturb or harm users? (security/safety)
    a. • Prompt: Did the chatbot provided unwanted contact, actions or content?
    b. • Prompt: Did the chatbot provided inappropriate responses?

6. To what extent would you use the chatbot in the future?

7. How would you improve the chatbot? (future directions)
    a. • Prompt: How could the chatbot be more helpful?

    b. • Prompt: How could the chatbot be more user-friendly?

    c. • Prompt: What topics could be added to the chatbot?

Appendix 7.5 - Chatbot outcomes questionnaires

UMUX-Lite (global usability)
Likert scale 1-7: 1 = strongly disagree, 7 = strongly agree

1. The chatbot's capabilities meet my requirements.
2. The chatbot is easy to use.

AES -Modified (acceptability)
Likert scale 1-5

3. How easy was the chatbot for you to use? 1 = very difficult, 5 = very easy
4. How understandable were the chatbot's answers to your questions? 1 = difficult to understand, 5 = easy to understand
5. How much did you enjoy using the chatbot? 1 = not at all, 5 = very much
6. How helpful was the chatbot in answering your questions? 1 = very unhelpful, 5 = very helpful
7. Was the amount of time it took to use the chatbot acceptable? 1 = very unacceptable, 5 = very acceptable
8. How would you rate your overall satisfaction with the chatbot? 1 = very dissatisfied, 5 = very satisfied

Compatibility subscale - Modified (appropriateness - for healthcare professionals)
Likert scale 1-7: 1 = Extremely disagree, 7 = Extremely agree

9. Using a chatbot is compatible with all aspects of my work.
10. I think that using a chatbot fits well with the way I like to work.
11. Using a chatbot fits into my work style.

IAM (appropriateness - for patients)
Likert scale 1-5: 1 = Completely disagree, 5 = Completely agree

12. The chatbot seems fitting for my self-care and management.
13. The chatbot seems suitable for my self-care and management.
14. The chatbot seems applicable for my self-care and management.
15. The chatbot seems like a good match for my self-care and management.
